# Design and Synthesis of 6‐*O*‐Phosphorylated Heparan Sulfate Oligosaccharides to Inhibit Amyloid β Aggregation

**DOI:** 10.1002/cbic.202200191

**Published:** 2022-06-16

**Authors:** Kenji Uchimura, Kazuchika Nishitsuji, Li‐Ting Chiu, Takashi Ohgita, Hiroyuki Saito, Fabrice Allain, Veeranjaneyulu Gannedi, Chi‐Huey Wong, Shang‐Cheng Hung

**Affiliations:** ^1^ Univ. Lille, CNRS UMR 8576 – UGSF - Unité de Glycobiologie Structurale et Fonctionnelle 59000 Lille France; ^2^ Department of Biochemistry Wakayama Medical University 811–1 Kimiidera Wakayama 641-8509 Japan; ^3^ Genomics Research Center Academia Sinica, 128, Section 2 Academia Road Taipei 11529 Taiwan; ^4^ Department of Biophysical Chemistry Kyoto Pharmaceutical University, 5 Misasagi-Nakauchi-cho Yamashina-ku Kyoto 607-8414 Japan; ^5^ Department of Chemistry The Scripps Research Institute 10550 North Torrey Pines Road BCC 338 La Jolla CA 92037 USA; ^6^ Department of Applied Science National Taitung University 369, Section 2 University Road Taitung 95092 Taiwan

**Keywords:** amyloid, atomic force microscopy, chemical synthesis, glycosaminoglycan, heparan sulfate

## Abstract

Dysregulation of amyloidogenic proteins and their abnormal processing and deposition in tissues cause systemic and localized amyloidosis. Formation of amyloid β (Aβ) fibrils that deposit as amyloid plaques in Alzheimer's disease (AD) brains is an earliest pathological hallmark. The polysulfated heparan sulfate (HS)/heparin (HP) is one of the non‐protein components of Aβ deposits that not only modulates Aβ aggregation, but also acts as a receptor for Aβ fibrils to mediate their cytotoxicity. Interfering with the interaction between HS/HP and Aβ could be a therapeutic strategy to arrest amyloidosis. Here we have synthesized the 6‐*O*‐phosphorylated HS/HP oligosaccharides and reported their competitive effects on the inhibition of HP‐mediated Aβ fibril formation *in vitro* using a thioflavin T fluorescence assay and a tapping mode atomic force microscopy.

## Graphical abstract

**A 6‐*O*‐phosphorylated** heparan sulfate tetrasaccharide was rationally designed and synthesized to inhibit heparin‐induced amyloid fibril formation of amyloid β (Aβ) *in vitro*. Its competitive effect was confirmed by a thioflavin T fluorescence assay and a tapping mode atomic force microscopy.

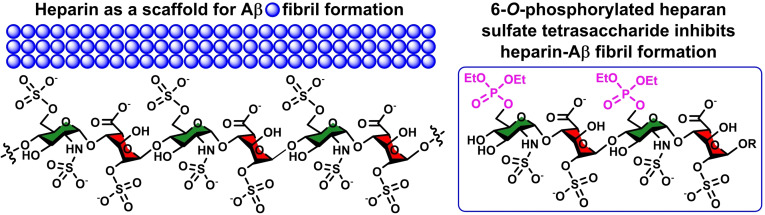

## Introduction

**Figure 1:**
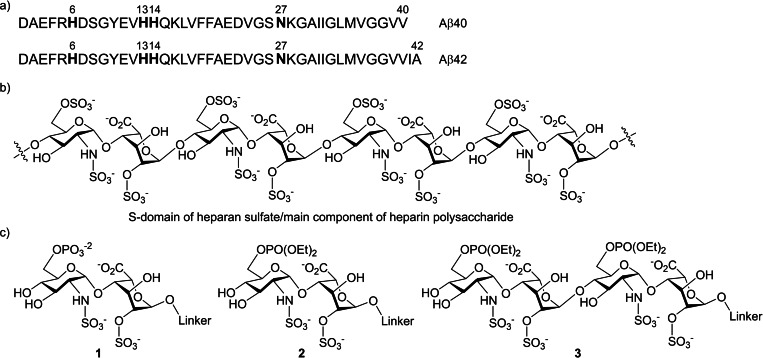
Structures of a) Aβ 40 and Aβ 42, b) S‐domain of heparan sulfate/major component of heparin polysaccharide, and c) 6‐*O*‐phosphorylated heparan sulfate analogues.

Amyloid plaques are fibrillar peptide aggregates that cause pathogenesis of a plethora of diseases, such as Alzheimer's disease (AD).^[1]^ AD is a neurodegenerative disorder characterized by a progressive loss of memory and dementia symptom among the elderly. Globally, it has become a major threat to human health and the number of AD patients is dramatically increasing year by year.^[2]^ Amyloid β (Aβ) peptides, the major components of amyloid fibrils that deposit as amyloid plaques in AD brains, are peptides of 38–43 amino acids proteolytically produced from the amyloid precursor protein.^[3]^ Because deposition of Aβ fibrils is an early pathological event of AD, the concept that Aβ accumulation is one of the major causes of AD has been widely accepted.^[4]^ Extracellular deposition of cytotoxic Aβ40 and Aβ42 (Figure 1, a) plaques in neurofibrillary tangles is the hallmark of AD.^[5]^ Various species of Aβ peptides can aggregate and form amyloid themselves; however, numbers of macromolecules have been reported to affect Aβ aggregation.^[6]^ Among these, the cell surface glycosaminoglycans (GAGs), which are linear, long and polysulfated polysaccharide chains have been identified as the non‐protein components of amyloid deposits *in vivo*,^[7]^ and have been implicated in the pathology of many protein aggregation diseases including AD.^[8]^ Heparan sulfate (HS) is a sulfated GAG consisting of 40–160 disaccharide units which are composed of a 2‐*O*‐sulfated or unsulfated uronic acid [D‐glucuronic acid (GlcA) or L‐iduronic acid (IdoA)] and a unsulfated, or 3‐*O*‐, 6‐*O*‐ or 3,6‐di‐*O*‐sulfated D‐glucosamine derivative [*N*‐acetylglucosamine (GlcNAc), *N*‐sulfated glucosamine (GlcNS), or unsubstituted glucosamine (GlcNH_2_)].^[9]^ The sulfation modifications of HS are enzymatic and strictly regulated, generating the structural diversity of HS chains and specific binding sites for their ligands. The highly sulfated S‐domain (Figure 1, b) of HS and its structurally related major component of heparin (HP) are composed of multiple trisulfated disaccharides, [IdoA(2‐OSO_3_)‐GlcNSO_3_(6‐OSO_3_)‐]_n_,^[10]^ which act as selective docking sites for interactions with various growth factors.^[11]^ In addition, the HS S‐domains and HP have been reported to act as scaffolds and are accumulated with amyloid deposits to form the cytotoxic amyloid fibrils in tissues of patients with AD, ATTR transthyretin amyloidosis, and p53‐mutated cancer.^[12]^ The atomic details of the interactions of a fully ^13^C,^15^N‐labeled HS/HP octasaccharide with Aβ 3Q‐fibrils have been elucidated, showing ionic interactions between the sulfate groups and the N‐terminal imidazo groups of H6 and H13/H14 as well as hydrogen bonding with N27 (Figure 1, a).^[13]^ The human extracellular sulfatases, SULFs, which selectively liberate 6‐*O*‐sulfates within the HS S‐domains and HP, could lessen the “amyloidosis‐promoting” functions. Furthermore, over‐expression of heparanase which catalyzes the cleavage of polymeric HS/HP molecules into short oligosaccharide chains to compete with HS/HP for interaction with Aβ peptides has been shown to reduce Aβ peptide aggregation and provide beneficial effects in amyloidosis in transgenic mouse models.^[14]^ These results have revealed that the short glycans from HS/HP with minimal 6‐*O*‐sulfation may serve as potential therapeutic agents to interfere with the interactions between HS/HP and amyloidogenic proteins.

Here we reported the synthesis of 6‐*O*‐phosphorylated HS/HP di‐ and tetrasaccharides 1–3 (Figure 1, c) and their inhibitory effects on Aβ42 fibril formation using a thioflavin T fluorescence assay and a trapping mode atomic force microscopy (AFM) analysis.

## Results and Discussion

**Scheme 1:**
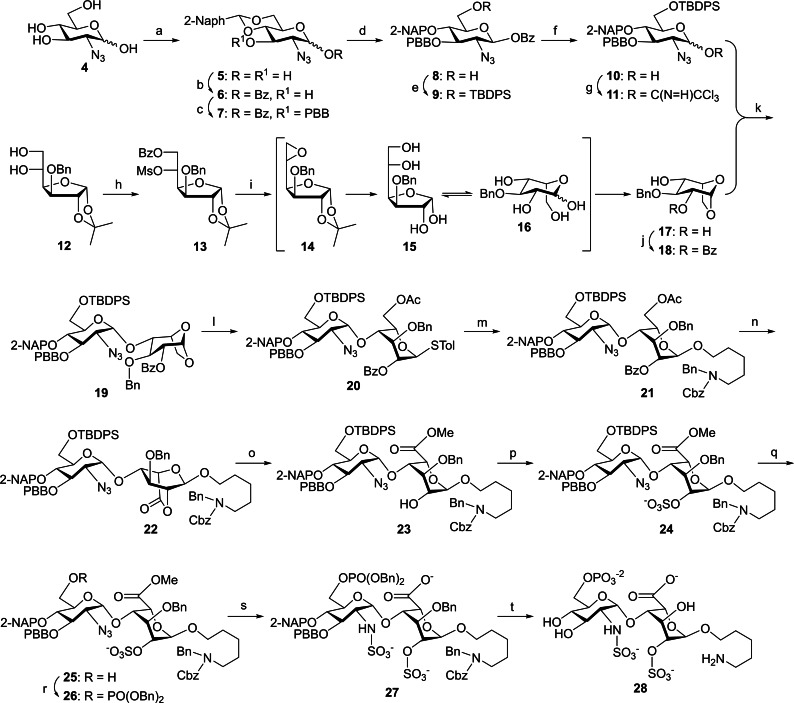
Synthesis of the 6’‐*O*‐phosphorylated HS/HP disaccharide **28**: a) cat. CSA, 2‐NaphCH(OMe)_2_, DMF, rt, 79 %; b) Bz_2_O, Et_3_N, CH_2_Cl_2_, RT, 16 h, 80 %; c) Ag_2_O, p‐bromobenzyl bromide, CH_2_Cl_2_, MS 4 Å, rt, 3 d, 86 %; d) BH_3_/THF, Cu(OTf)_2_, 0 °C, 6 h, 84 %; e) TBDPSCl, Et_3_N, DMAP, CH_2_Cl_2_, 0 °C to rt, 24 h, 93 %; f) sat. NH_3_, MeOH/THF=3/7, 0 °C, 24 h, 86 %; g) K_2_CO_3_, CCl_3_CN, CH_2_Cl_2_, 0 °C to rt, 24 h, 97 %; h) BzCl, Pyr, CH_2_Cl_2_, 0 °C; MsCl, 0 °C to rt, 81 % in one‐pot; i) 1. ^t^BuOK, ^t^BuOH, CH_2_Cl_2_, 0 °C; 2. 3 M H_2_SO_4(aq)_, 1,4‐dioxane, 120 °C, 1 d, 61 % in one‐pot; j) BzCl, Pyr, CH_2_Cl_2_, 0 °C, 85 %; k) TMSOTf, MS 4 Å, CH_2_Cl_2_, −78 to 0 °C, 2 h, 84 %; l) 1. Cu(OTf)_2_, Ac_2_O, 0 °C to rt, 3 h; 2. TMSSTol, ZnI_2_, CH_2_Cl_2_, rt, 2 h, 77 % in two steps; m) NIS, TfOH, HO(CH_2_)_5_N(Bn)Cbz, MS 3 Å, CH_2_Cl_2_ /CH_3_CN=1/2, −78 to −40 °C, 3 h, 77 %; n) 1. NaOMe, CH_2_Cl_2_, MeOH, rt, 18 h; 2. TEMPO, BAIB, CH_2_Cl_2_/H_2_O=2/1, rt, 16 h, 67 % in two steps; o) MeOH, Et_3_N, CH_2_Cl_2_, 40 °C, 18 h, 83 %; p) SO_3_/Et_3_N, DMF, 60 °C, 18 h, 75 %; q) HF/Pyr, Pyr, THF, 0 °C to rt, 3 d, 60 %; r) 1. dibenzyl *N*,*N*‐diisopropylphosphoramidite, 1H‐tetrazole, DMF/CH_2_Cl_2_=1/9; 2. mCPBA, 18 h, 65 %; s) 1. LiOH(aq), H_2_O_2_, THF, 37 °C, 18 h; 2. PMe_3_/THF, THF, NaOH_(aq)_, rt, 14 h; 3. SO_3_/Pyr, Et_3_N, NaOH_(aq)_, MeOH, rt, 18 h, 56 % in three steps; t) 20 % Pd(OH)_2_/C, H_2(g)_ balloon, MeOH, phosphate buffer (pH=7), rt, 3 d,78 %.

The preparation of HS/HP oligosaccharides bearing different chain lengths and sulfation patterns has attracted great attentions from the chemistry community. Chemical^[15]^ and chemo‐enzymatic^[16]^ synthesis are currently the common methods used to synthesize the structurally well‐defined saccharides. Scheme 1 illustrates our efficient route for the synthesis of the 6’‐*O*‐phosphorylated HS/HP disaccharide **28**. The orthogonally protected glycosyl donor **11** was prepared from 2‐azido‐2‐deoxy‐D‐glucosamine **4** ^[17]^ in 7 steps. Reaction of the 1,3,4,6‐tetraol **4** with 2‐NaphCH(OMe)_2_ in the presence of camphorsulfonic acid as catalyst furnished the 4,6‐*O*‐naphthylidene compound **5** in 79 % yield. Regioselective benzoylation of the 1,3‐diol **5** with benzoic anhydride and triethylamine at the 1‐O position yielded the desired 1‐benzoate **6** (80 %), which was subjected to *p*‐bromobenzylation (*p*‐BrBnBr, Ag_2_O) at 3‐O affording the expected ether product **7** (86 %). Cu(OTf)_2_‐catalyzed regioselective borane‐reductive ring opening of compound **7** at 6‐O gave the 6‐alcohol **8** (84 %),^[18]^ which underwent the TBDPS‐protection (TBDPSCl, Et_3_N, DMAP) and the 6‐*O*‐silyl ether **9** was obtained in 93 % yield. Debenzoylation of **9** with ammonia provided the 1‐alcohol **10** (86 %), which was transformed into the corresponding trichloroacetimidate **11** (K_2_CO_3_, CCl_3_CN, 97 %) as a donor for further glycosylation.

For the synthesis of the 1,6‐anhydro‐β‐L‐idopyranosyl acceptor **18** (Scheme 1), the 5,6‐diol **12**, which was generated from commercially available diacetone glucose via a combination of traditional Williamson 3‐*O*‐benzylation and acidic hydrolysis of the 5,6‐*O*‐isopropylidene group, underwent regioselective benzoylation at the less hindered 6‐O position followed by mesylation at 5‐O and gave the 6‐OBz‐5‐OMs product **13** in 81 % yield in a one‐pot manner. Conversion of **13** into the 1,6‐anhydro‐β‐L‐ido sugar **17** (61 %) was successfully carried out via consecutive treatment of ^t^BuOK and 3 M H_2_SO_4(aq)_ in the same reaction flask in accordance with our previous report.^[15a]^ The 6‐*O*‐benzoyl group of **13** was cleaved by ^t^BuOK to generate the corresponding alkoxide, which underwent intramolecular cyclization to give an epoxide **14** with the L‐ido configuration. Acidic hydrolysis of the epoxide ring and the 1,2‐isopropylidene group yielded the L‐idofuranose **15**, which was equilibrated to the L‐idopyranose **16** followed by elimination of a water to furnish the 1,6‐anhydro derivative **17** in one‐pot. The 2,4‐diol **17** was regioselectively benzoylated at 2‐O (BzCl, pyridine) and the expected 4‐alcohol **18** was isolated in 85 % yield. Coupling of both **11** and **18** in the presence of TMSOTf as catalyst afforded the desired *α*‐linked disaccharide **19** (84 %), which was subjected to Cu(OTf)_2_‐catalyzed acetolysis and ZnI_2_‐promoted addition of TMSSTol providing the corresponding thioglycoside **20** in 77 % yield. Coupling of **20** with the linker HO(CH_2_)_5_N(Bn)Cbz using NIS/TfOH yielded the *α*‐form disaccharide **21** (77 %) influenced by the neighboring group participation of the 2‐*O*‐benzoyl group. Removal of the acyl groups in **21** via Zemplén transesterification gave the 2,6‐diol intermediate, which was oxidized by TEMPO/BAIB to give the lactone **22** in 67 % yield in two steps. Ring opening of the lactone **22** under methanolysis conditions furnished the 2‐alcohol **23** (83 %), which underwent 2‐*O*‐sulfonation (SO_3_/Et_3_N) to provide the sulfate **24** in 75 % yield. The TBDPS group was deprotected by HF/pyridene, and the 6’‐alcohol **25** (60 %) was obtained for further phosphorylation.

Reaction of compound **25** with dibenzyl *N*,*N*‐diisopropylphosphoramidite was activated by 1*H*‐tetrazole in a mixture of DMF/CH_2_Cl_2_ (1/9) to yield the phosphite intermediate, which was oxidized by *m*‐CPBA to afford the phosphate **26** (65 % in two steps). Saponification of the methyl ester **26** with LiOH and H_2_O_2_ followed by azido reduction with PMe_3_/THF and NaOH led to the corresponding amine, which was treated with SO_3_/pyridene to furnish the sulfate derivative **27** (56 % yield in three steps). Finally, cleavage of all Bn, 2‐NAP, Cbz, and PBB groups in **27** under hydrogenolysis conditions with H_2_, Pd(OH)_2_/C in methanol and phosphate buffer at for 3 days successfully gave the target phosphate **28** in 78 % yield.

**Scheme 2:**
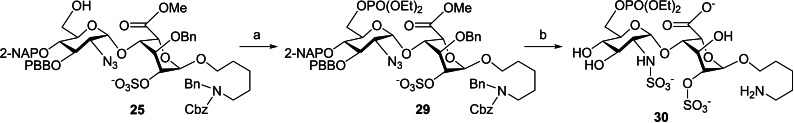
Synthesis of diethylphosphonato heparin disaccharide **30**: a) ClPO(OEt)_2_, Pyr, rt, 2 h, 89 %; b) 1. LiOH_(aq)_, H_2_O_2_, THF, 37 °C, 18 h; 2. PMe_3_/THF, THF, NaOH_(aq)_, rt, 14 h; 3. SO_3_/Pyr, Et_3_N, NaOH_(aq)_, MeOH, rt, 18 h; 4. 20 % Pd(OH)_2_/C, H_2(g)_ balloon, MeOH, phosphate buffer (pH=7), rt, 3 d, 41 %, in four steps.

Our approach to the synthesis of the diethyl phosphate **30** is depicted in Scheme 2. Treatment of the 6’‐alcohol **25** with diethyl chlorophosphate in pyridine provided the protected phosphate **29** (89 %). Transformation of **29** into the expected final molecule **30** was carried out by a four‐step procedure in 41 % overall yield, including 1) hydrolysis of the methyl ester under basic conditions (LiOH and H_2_O_2_); 2) Staudinger reaction of the azido group (PMe_3_/THF and NaOH); 3) *N*‐sulfonation with SO_3_/pyridine; and 4) global deprotection of all benzyl‐type groups by hydrogenolysis.

**Scheme 3:**
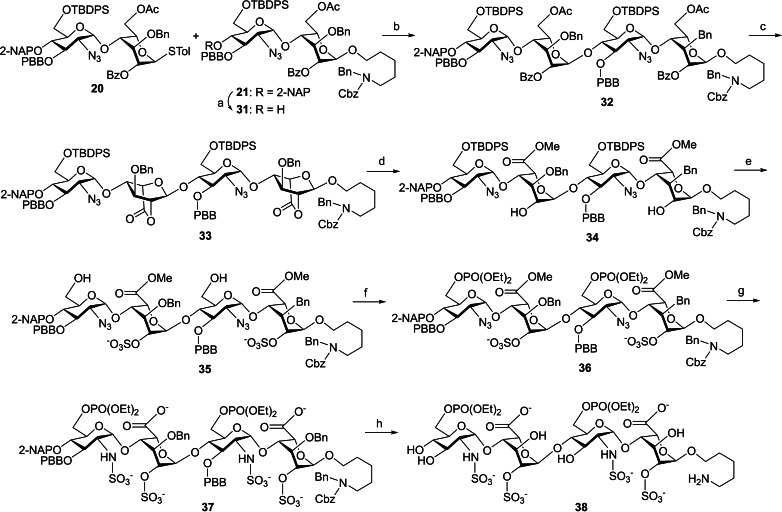
Synthesis of diethylphosphonato heparin tetrasaccharide **38**: a) DDQ, H_2_O, CH_2_Cl_2_, rt, 4 h, 75 %; b) NIS, TfOH, MS 3 Å, CH_2_Cl_2_, −78 to −20 °C, 2 h, 86 %; c) 1. NaOMe, CH_2_Cl_2_, MeOH, rt, 18 h; 2. TEMPO, BAIB, CH_2_Cl_2_/H_2_O=2/1, rt, 16 h, 70 % in two steps; d) Et_3_N, MeOH, CH_2_Cl_2_, 40 °C, 18 h, 97 %; e) 1. SO_3_/Et_3_N, DMF, 60 °C, 18 h, 75 %; 2. HF/Pyr, Pyr, THF, 0 °C to rt, 3 d, 76 %; f) ClPO(OEt)_2_, Pyr, rt, 2 h, 90 %; g) 1. LiOH_(aq)_, H_2_O_2_, THF, 37 °C, 18 h; 2. PMe_3_/THF, THF, NaOH_(aq)_, rt, 14 h; 3. SO_3_/Pyr, Et_3_N, NaOH_(aq)_, MeOH, rt, 18 h, 36 % in three steps; h) 20 % Pd(OH)_2_/C, H_2(g)_ balloon, MeOH, phosphate buffer (pH=7), rt, 3 d, 62 %.

With compounds **20** and **21** in hand, we continued to prepare the 6‐*O*‐phosphorylated tetrasaccharide **38** employing a convergent strategy for backbone assembly (Scheme 3). The disaccharide acceptor **31**, which was generated in 75 % yield by DDQ‐cleavage of the 2‐NAP group in **21**, was coupled with the thioglycoside donor **20** via a combination of NIS and TfOH as activators to furnish the desired *α*‐linked tetrasaccharide **32** (86 %). The stereochemistry of **32** was determined by HSQC and TOCSY spectra (please see supporting information), and the newly formed *α*‐glycosidic bond was stereocontrolled by the neighboring group effect. All acetyl and benzoyl groups in **32** were removed under Zemplén's conditions, and the tetraol intermediate underwent TEMPO/BAIB oxidation to yield the dilactone **33** (70 % in two steps) through HMBC spectral confirmation for the formation of bicyclo[2.2.2] rings (please see supporting information). The dilactone **33** was opened with MeOH and Et_3_N to give the 2,2”‐diol **34** in 97 % yield. Reaction of **34** with SO_3_/Et_3_N afforded the 2,2”‐disulfate (75 %), which was subjected upon deprotection of both TBDPS groups, leading to the 6’,6’”‐diol **35** in 76 % yield. Compound **35** was further treated with diethyl chlorophosphite under basic conditions, and the corresponding diphosphate **36** was obtained in good yield (90 %). Basic hydrolysis of the methyl ester **36** to the dicarboxylate, conversion of both azido groups into the amino groups, and introduction of the sulfonate groups at the 2’‐N and 2’”‐N positions furnished the *N*,*O*‐tetrasulfate **37** (36 % overall yield in three steps), which was fully deprotected under hydrogenolysis conditions to provide the final target molecule **38** (62 %).

**Figure 2:**
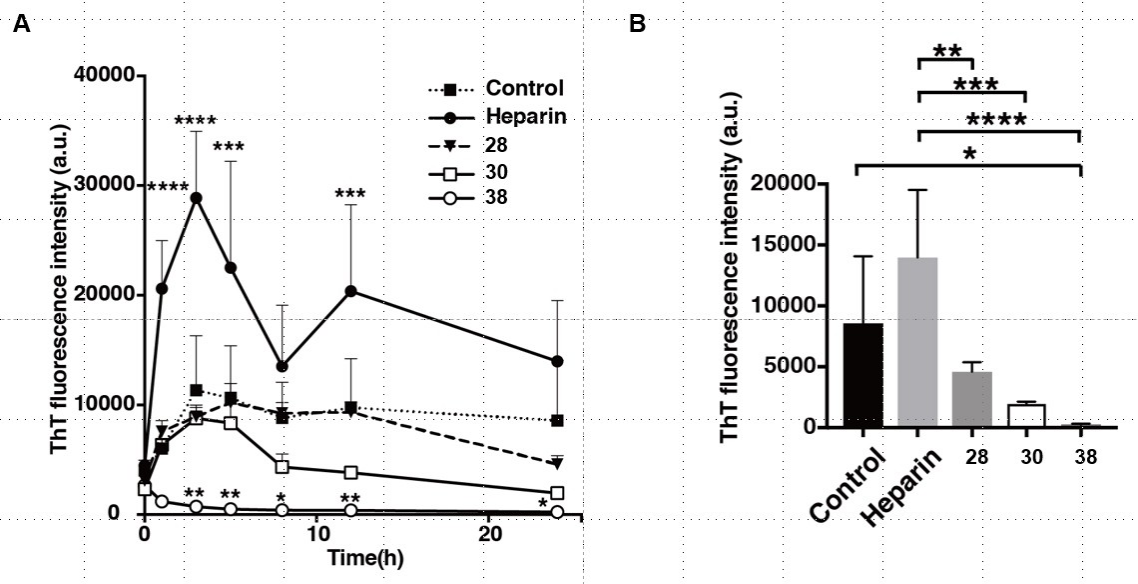
Effects of 6‐O‐phosphorylated heparan sulfate/heparin oligosaccharide derivatives on amyloid β fibril formation. (A,B) Fluorescence‐based Thioflavin T (ThT) binding assay of Amyloid β (Aβ) in the absence (Control) or presence of heparin, or HS/heparin oligosaccharide derivative. Aβ (10 μM) was incubated without or with heparin (40 μg/mL) or HS/heparin derivative (100 μM) in the mixture of ThT (10 μM) at 37 °C. ThT fluorescence intensity was measured for 24 h. Ordinary two‐way ANOVA with time of incubation and compound as factors revealed significant effects on Aβ aggregation as shown by ThT fluorescence intensity (time of incubation: F_6, 70_=10.47, P<0.0001; compound: F_4, 70_=71.58, P<0.0001; interaction between time and compound: F_24, 70_=2.924, P=0.0003). (A) Post‐hoc Dunnett's test (vs. Control) showed a significant change in Aβ aggregation, starting at 1 h post‐incubation and thereafter until 12 h post‐incubation in heparin‐mixed fractions, and starting at 3 h post‐incubation and thereafter until 24 h post‐incubation in **38**‐mixed fractions. (B) Post‐hoc Tukey's range test showed significant change in **28**‐, **30**, and **38**‐mixed fractions compared with heparin‐mixed fractions. Data are means±s.e. (n=3). *P<0.05, **P<0.01, ***P<0.001, ****P<0.0001.

To study the effect of Aβ fibrilization in the absence (Control) or presence of HP (Heparin sodium is the biopolymer used in the assays, which was manufactured from porcine intestinal mucosa, is commercially available. Its strength, quality and purity met the specifications (Anti‐Factor IIa activity: 204 USP units/mg, Anti‐Factor Xa activity: 195 units/mg, Anti‐clotting activity: 197 IU/mg, Bacterial endotoxins: <0.0015 EU/u.) or the 6‐*O*‐phosphorylated HS/HP analogues **28**, **30**, and **38**, an assay was established to measure the thioflavin T (ThT) fluorescence for 24 hours (Figure 2).^[19]^ Aβ (10 μM) was incubated without or with HP (40 μg/mL) or **28**, **30**, and **38** (100 μM) in the mixture of ThT (10 μM) at 37 °C. The ordinary two‐way ANOVA with time of incubation and compound as factors revealed significant effects on Aβ aggregation as shown by the ThT fluorescence intensity (time of incubation: F_6, 70_=10.47, P<0.0001; compound: F_4, 70_=71.58, P<0.0001; interaction between time and compound: F_24, 70_ =2.924, P=0.0003). In accordance with the literature reports, we found that HP enhanced Aβ fibril formation with a 2‐ to 3‐fold increase in intensity as compared to the control (Figure 2, A).^[20]^ The disaccharides **28** and **30** exhibited effects comparable to the control on the lag phase and elongation phase of Aβ aggregation in the 6 h incubation period and less fluorescence intensities in the stationary phase after 24 h incubation. Compared to that of heparin, **28** and **30** showed 33 % and 14 % effects on Aβ aggregations, respectively (Figure 2, B). The results suggested that the diethyl phosphate group at the 6’‐C position of **30** had a better inhibition of Aβ fibrillization than the phosphate group in **28**. Interestingly, the tetrasaccharide derivative **38** bearing two diethyl phosphate groups at 6’‐C and 6’” exhibited the intensities much less than the control over the lag, elongation and stationary phases of Aβ aggregation with 3 to 6 % of control in the period of 3–24 h and 2 % of heparin till 24 h (Figure 2, A & B). The results have revealed that compound **38** might trap the native Aβ monomers, and interfere with their conversion to amyloidogenic monomers (Figure 2A). Here we propose that the mechanism of action of the compounds is the disaggregation of the initial oligomers due to their short lengths and the prevention of oligomerization generated by longer chains of HS. However, it would be valuable to study the exact mechanisms of these derivatives in future research, as this would provide insight into fibril formation.

**Figure 3:**
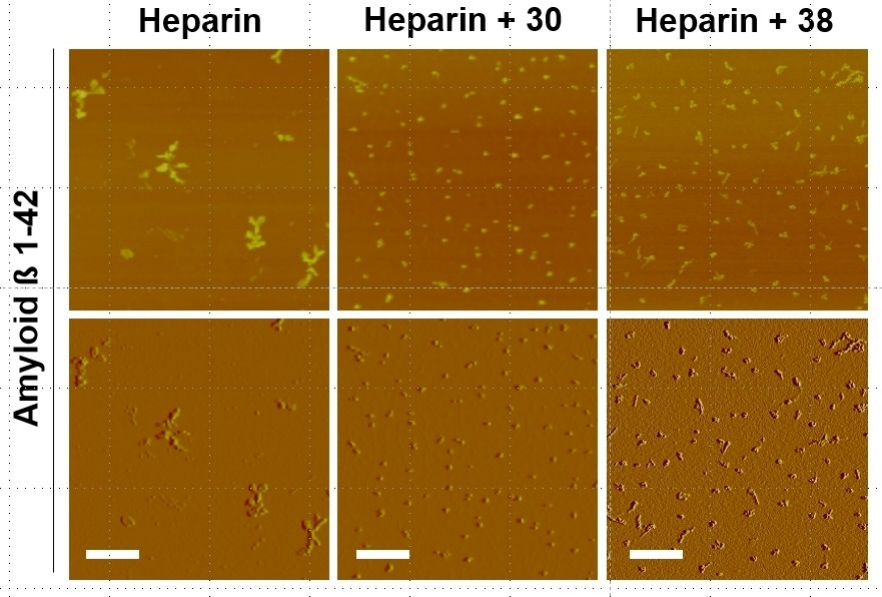
Morphological analysis of amyloid β fibrils assembled in the presence of heparin and the chemically synthesized heparan sulfate/heparin derivatives. Atomic force microscopy images in tapping mode high (z range: 100.00 nm, *top*) and amplitude in deflection (z range: 1.00 V for Heparin and Heparin+**30**, 0.25 V for Heparin+**38**, *bottom*) of Aβ assemblies are shown. These assemblies were prepared in the presence of heparin (40 μg/mL) alone, or co‐presence of heparin (40 μg/mL) and **30** or **38** HS/heparin derivative (100 μM). Represent images of two independent experiments are shown. Scale bar: 1 μm.

Morphological analyses of Aβ aggregations in the presence of HP, HP+**30**, and HP+**38** by tapping mode atomic force microscopy (AFM) are depicted in Figure 3. When Aβ and HP alone were mixed, Aβ fibrils were formed in a short and twisted morphology. In the study of Aβ, HP and the disaccharide **30**, which didn't affect the lag phase of Aβ aggregation, the Aβ assemblies resembled small and spherical oligomers that are proposed to be “on‐pathway” intermediates for fibril formation. ^[21]^ It is known that “on‐pathway” intermediates can further elongate to form mature fibrils. Since the assembly of mature fibrils was not observed, it was suggested that disaccharide **30** might interfere with the elongation process in the co‐presence of HP, possibly by masking the GAG‐binding site of the spherical conformers. In the co‐existence of HP and the tetrasaccharide **38**, the Aβ assemblies were smaller and less‐elongated than those with heparin alone (Figure 3). The tetrasaccharide **38** could have an ability to insulate the GAG‐binding site of Aβ monomers, and compete with HP and sequestered Aβ monomers with native or misfolded conformations and/or small oligomers *in vivo*, but did not act as a scaffold for Aβ fibril formation due to its short length.

## Conclusion

In summary, we have successfully synthesized 6‐O‐phosphorylated HS/HP saccharides **28**, **30**, and **38**. The analogues **30** and **38** have inhibitory effects on Aβ fibril formation in the presence of HP as measured by the ThT fluorescence intensities and studied by atomic force microscopy. Because the transition of an unstructured state to a *β*‐sheet structure upon aggregation can create sulfated GAG‐binding sites, compounds **30** and **38** may bind to these conformers and interfere with the subsequent elongation process, but are unable to serve as scaffolds due to their short saccharide chain lengths. The new findings that the small sulfated and phosphorylated glycans could interfere with the interaction between HS/HP and Aβ provide significant information for the understanding of amyloid plaque formation and development of new therapeutics to ameliorate the associated diseases.

## Conflict of interest

The authors declare no conflict of interest.

1

## Supporting information

As a service to our authors and readers, this journal provides supporting information supplied by the authors. Such materials are peer reviewed and may be re‐organized for online delivery, but are not copy‐edited or typeset. Technical support issues arising from supporting information (other than missing files) should be addressed to the authors.

Supporting Information

## Data Availability

The data that support the findings of this study are available from the corresponding author upon reasonable request.
