# Supplementary material for: Design and Synthesis of 6‐O‐Phosphorylated Heparan Sulfate Oligosaccharides to Inhibit Amyloid β Aggregation
Source: Chembiochem. 2022 Jun 16;23(15):e202200191. doi: 10.1002/cbic.202200191 (PMC9401075; doi:10.1002/cbic.202200191)
Supplement: Supplementary file 1 — Supporting Information [file CBIC-23-0-s001.pdf]

ChemBioChem

Supporting Information

Design and Synthesis of 6-*O*-Phosphorylated Heparan Sulfate Oligosaccharides to Inhibit Amyloid β Aggregation

Kenji Uchimura,* Kazuchika Nishitsuji[+], Li-Ting Chiu[+], Takashi Ohgita, Hiroyuki Saito, Fabrice Allain, Veeranjaneyulu Gannedi, Chi-Huey Wong,* and Shang-Cheng Hung*

Table of Contents

1. General Procedures …………………………………..……………………………… S02
2. Experimental Procedures
Compound **19** ………………………………………………………..…… ………. S03
Compound **20** ………………………………………………………..…… ………. S04
Compound **21** ………………………………………………………..…… ………. S05
Compound **22** ………………………………………………………..…… ………. S06
Compound **23** ………………………………………………………..…… ………. S07
Compound **24** ………………………………………………………..…… ………. S08
Compound **25** ………………………………………………………..…… ………. S09
Compound **26** ………………………………………………………..…… ………. S10
Compound **27** ………………………………………………………..…… ………. S11
Compound **28** ………………………………………………………..…… ………. S13
Compound **29** ………………………………………………………..…… ………. S13
Compound **30** ………………………………………………………..…… ………. S14
Compound **31** ………………………………………………………..…… ………. S16
Compound **32** ………………………………………………………..…… ………. S17
Compound **33** ………………………………………………………..…… ………. S18
Compound **34** ………………………………………………………..…… ………. S20
Compound **35** ………………………………………………………..…… ………. S21
Compound **36** ………………………………………………………..…… ………. S22
Compound **37** ………………………………………………………..…… ………. S23
Compound **38** ………………………………………………………..…… ………. S25
3. Biological Procedures ……………………………………………..…………………… S25
4. References ……………………………………………………………………...……….. S26
5. NMR spectra………………………………….....…………………...…………….... S27

1. General Procedures

All reactions were performed under an atmosphere of nitrogen/argon in flame-dried glassware. Solvents were distilled in the standard way, and commercial reagents were used without any purification unless otherwise stated. Anhydrous solvents like CH_2Cl_2, Et_2O, DMF, and Et_3N were dried in a standard way. TLC was performed on glass plates pre-coated with Silica Gel 60 F_{254} (0.25 mm, E. Merck). TLC plates were visualized by exposure to ultraviolet light (UV-254 nm) and/or detection was executed by spraying with a solution of $Ce(NH_4)_2(NO_3)_6$, $(NH_4)_6Mo_7O_{24}$, and H_2SO_4 in water and subsequent heating on a hot plate. Specific rotations were taken at ambient conditions and reported in $10^{-1}\cdot deg\cdot cm^2\cdot g^{-1}$; the sample concentrations are in $g\cdot dL^{-1}$. Flash column chromatography was carried out on Silica Gel 60 (230–400 mesh, E. Merck) or MP. ^1H NMR spectra were recorded on 600 MHz instrument at ambient temperature. Data were recorded as follows: chemical shift in ppm from the solvent resonance employed as the internal standard ($CDCl_3$ at 7.26 ppm, and CD_3OD at 3.31 ppm and 4.78 ppm), multiplicity (s = singlet, d = doublet; t = triplet; q = quartet; st = septet; m = multiplet; br = broad), coupling constant (Hz), integration. ^{13}C NMR spectra were measured on 150 MHz spectrometer. Chemical shifts were recorded in ppm from the solvent resonance employed as the internal standard ($CDCl_3$ at 77.23 ppm, and CD_3OD at 49.3 ppm). Mass spectra were obtained with ESI Finnign LCQ mass spectrometer (Thermo Finnigan), performed at Genomics Research Center. HPLC analysis were performed using an HPLC system (HP-2100, Agilent), consisting of a quaternary gradient pump (Agilent 1100), conductivity detector (PROD, AERS500, 4 mm, Thermal), reagent-free controller (PROD. RFC10) and autosampler (Agilent 1260 infinity) system with and acetonitrile-water mobile phase and UV detection. Prior to all the glycosylation, the starting materials were dried under high vacuum overnight in a desiccator.

2. Experimental Procedures

Compound 19.

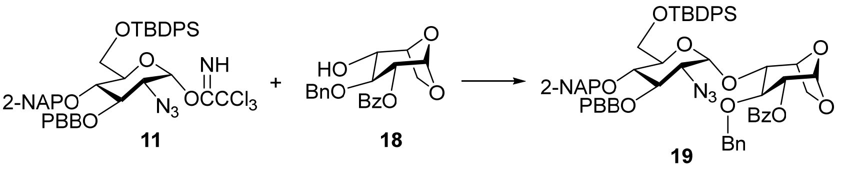

A mixture of the D-glucosamine-derived imidate **11**[1] (20.0 g, 22.3 mmol), the 1,6-anhydro-β-L-idopyranosyl 4-alcohol **18**[2] (11.9 g, 33.4 mmol) and freshly dried 4 Å MS (40.0 g) in dichloromethane (400 mL) was stirred at room temperature for 0.5 h under nitrogen atmosphere. The reaction flask was cooled to –78 °C, and trimethylsilyl trifluoromethanesulfonate (600 μL, 3.35 mmol) was added to the solution. The mixture was gradually warm up to 0 °C, and the resulting solution was kept stirring for 2 h. Triethylamine (2.0 mL) was added to quench the reaction, and the whole mixture was filtered through a pad of celite followed by wash with dichloromethane. The filtrate was concentrated *in vacuo* to furnish a residue, which was purified by flash column chromatography (EtOAc/Hex = 1/18) to yield the disaccharide **19** (20.5 g, 84 %). ^{1}H NMR (600 MHz, CDCl3) δ 8.09-8.05 (m, 2H, Ar-H), 7.85-7.83 (m, 1H, Ar-H), 7.76 (d, *J* = 7.9 Hz, 2H, Ar-H), 7.70-7.66 (m, 4H, Ar-H), 7.60-7.58 (m, 2H, Ar-H), 7.52-7.50 (m, 2H, Ar-H), 7.48-7.41 (m, 6H, Ar-H), 7.39-7.33 (m, 4H, Ar-H), 7.39-7.22 (m, 9H, Ar-H), 5.53 (d, *J* = 1.8 Hz, 1H, H-1), 5.30 (d, *J* = 3.8 Hz, 1H, H-1'), 5.09 (dd, *J* = 8.1, 1.8 Hz, 1H, H-2), 4.95-4.87 (m, 4H, $ArCH_2$), 4.79 (t, *J* = 10.6 Hz, 2H, $ArCH_2$), 4.59 (t, *J* = 4.6 Hz, 1H, H-5), 4.15 (d, *J* = 7.7 Hz, 1H, H-6a), 4.08 (t, *J* = 8.1 Hz, 1H, H-3), 4.04-3.97 (m, 2H, H-4, H-3'), 3.93-3.89 (m, 2H, H-6'), 3.77-3.67 (m, 3H, H-4', H-5', H-6b), 3.43 (dd, J = 10.4, 3.7 Hz, 1H, H-2'), 1.09 (s, 9H, TBDPS). ^{13}C NMR (150 MHz, CDCl3) δ 165.9 (C), 138.1 (C), 136.9 (C), 135.9 (CH), 135.8 (CH), 135.0 (C), 133.6 (C), 133.4 (C), 133.3 (C), 133.2 (CH), 132.9 (C), 131.8 (CH), 130.04 (CH), 130.0 (CH), 129.8 (CH), 129.6 (CH), 128.64 (CH), 128.6 (CH), 128.5 (CH), 128.1 (CH), 128.05 (CH), 127.97 (CH), 127.93 (CH), 127.9 (CH), 126.9 (CH), 126.5 (CH), 126.4 (CH), 125.9 (CH), 122.1(C), 99.7 (CH), 99.4 (CH), 80.2 (CH), 79.5 (CH), 79.0 (CH), 78.4 (CH), 77.2 (CH), 75.7 (CH_2), 75.1 (CH_2), 74.8 (CH_2), 74.4 (CH), 72.9 (CH), 65.8 (CH_2), 63.7 (CH), 62.7 (CH_2), 26.9 (CH_3), 19.4 (C). HRMS m/z (ESI, MNa^+) calcd. for $C_{60}H_{60}N_3O_{10}NaSiBr$, 1112.3129; found, 1112.3123.

Compound 20.

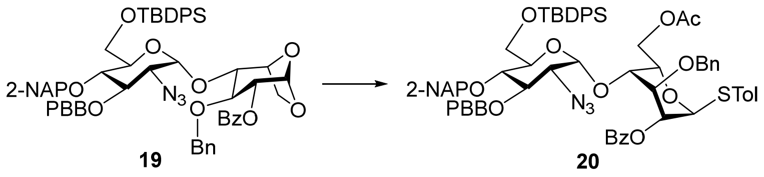

To a solution of compound **19** (17.0 g, 15.5 mmol) in acetic anhydride (51.0 mL) was added copper(II) triflate (57 mg, 157 µmol) at 0 ºC under nitrogen atmosphere. After stirring overnight, the reaction was quenched by sat. $NaHCO_{3(aq)}$, and the mixture was extracted with ethyl acetate (3 x 250 mL). The combined organic layer was washed with brine, dried over $MgSO_4$, filtered, and concentrated *in vacuo*. The residue was purified by flash column chromatography on silica gel (EtOAc/Hex = 1/4) to afford the desired 1,6-diacetate (18.6 g, 15.6 mmol). This crude compound was dissolved in dichloromethane (190 mL) at room temperature under nitrogen atmosphere. TMSSTol (18.0 mL, 77.9 mmol) and ZnI_2 (11.9 g, 46.7 mmol) were sequentially added to the mixture, and the resulting solution was kept stirring for 2 h. The mixture was filtered through a pad of celite to remove ZnI_2 followed by wash with dichloromethane. The organic layer was neutralized with sat. $NaHCO_{3(aq)}$, dried over anhydrous $MgSO_4$, filtered, and concentrated *in vacuo*. The crude residue was purified by flash column chromatography (EtOAc/Hex = 1/18) on silica gel to afford the thioglycoside **20** (15.1 g, 77% in two steps). ^{1}H NMR (600 MHz, $CDCl_3$) δ 8.15-8.13 (m, 2H, Ar-H), 7.87-7.86 (m, 1H, Ar-H), 7.79-7.77 (m, 2H, Ar-H), 7.70-7.65 (m, 4H, Ar-H), 7.59 (s, 1H, Ar-H), 7.54-7.50 (m, 4H, Ar-H), 7.48-7.46 (m, 2H, Ar-H), 7.43-7.25 (m, 17H, Ar-H), 7.13 (d, *J* = 7.9 Hz, 2H, Ar-H), 6.94 (d, *J* = 8.5 Hz, 2H, Ar-H), 5.54 (s, 1H, H-1), 5.40 (t, *J* = 2.5 Hz, 1H, H-2), 5.01 (d, *J* = 11.6 Hz, 1H, $ArCH_2$), 4.92-4.90 (m, 1H, H-5), 4.87-4.83 (m, 2H, $ArCH_2$), 4.78 (d, *J* = 11.6 Hz, 1H, $ArCH_2$), 4.62 (d, *J* = 3.6 Hz, 1H, H-1'), 4.40 (dd, *J* = 11.5, 8.2 Hz, 1H, H-6a), 4.32 (d, *J* = 10.8 Hz, 1H, $ArCH_2$), 4.18-4.15 (m, 2H, H-3, H-6), 4.03-3.98 (m, 2H, H-6a', $ArCH_2$), 3.91 (d, *J* = 11.7 Hz, 1H, H-6b'), 3.79-3.77 (m, 2H, H-4', H-5'), 3.65-3.61 (m, 2H, H-4, H-3'), 3.33 (dd, *J* = 10.3, 3.6 Hz, 1H, H-2'), 2.35 (s, 3H, $ArCH_3$), 1.95 (s, 3H, $COCH_3$), 1.08 (d, *J* = 2.2 Hz, 9H, TBDPS). ^{13}C NMR (150 MHz, $CDCl_3$) δ 170.39 (C), 165.71 (C), 137.77(C), 137.34 (C), 136.65 (C), 135.88 (CH), 135.59 (CH), 135.42 (CH), 133.44 (CH), 133.25 (CH), 133.15 (CH), 133.0 (CH), 132.9 (CH), 132.4 (CH), 131.7 (CH), 131.5 (CH), 129.9 (CH), 129.8 (CH), 129.7 (CH), 129.7 (CH), 129.7 (CH), 129.6 (CH), 128.5 (CH), 128.5 (CH), 128.3 (CH), 128.2 (CH), 128.1 (CH), 127.9 (CH), 127.7 (CH), 127.6 (CH), 126.3 (CH), 126.2 (CH), 126.0 (CH), 125.7

(CH), 121.7 (CH), 98.9 (CH), 86.3 (CH), 80.7 (CH), 77.8(CH), 75.1 (CH_2), 74.2 (CH_2), 73.1 (CH), 72.8 (CH_2), 71.8 (CH), 69.7 (CH), 66.3 (CH), 64.0 (CH), 63.2 (CH_2), 62.2 (CH_2), 26.9 (CH_3), 21.1 (CH_3), 20.74 (CH_3); HRMS m/z (ESI, MNa^+) calcd. for $C_{69}H_{70}BrN_3O_{11}SSiNa$, 1278.3576; found, 1278.3698.

Compound 21.

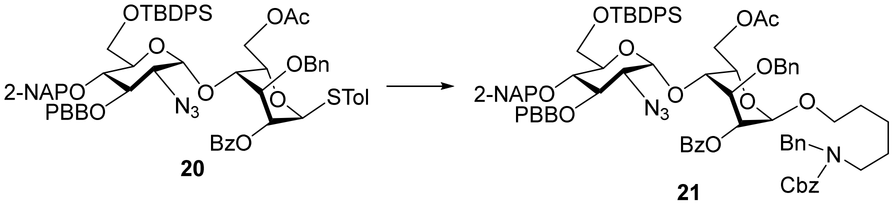

A mixture of the thioglycoside **20** (5.0 g, 3.98 mmol), $HO(CH_2)_5N(Bn)Cbz$ (1.4 g, 4.37 mmol) freshly activated 3 Å MS (6.0 g), dichloromethane (33 mL) and acetonitrile (66 mL) was stirred at room temperature for 30 min under nitrogen atmosphere. The reaction flask was then cooled down to –78 °C, *N*-iodosuccinimide (1.07 g, 4.75 mmol) was added to the mixture, and the resulting solution was kept stirring at the same temperature for 0.5 h. Triflic acid (140 μL, 1.59 mmol) was added to the solution, and the mixture was gradually warm up to –40 °C for 3 h. The reaction was quenched by 10% $Na_2S_2O_3$ followed by neutralization with sat. $NaHCO_3$. The crude mixture was filtered through a pad of celite and washed with CH_2Cl_2. The organic layer was washed with water, dried over anhydrous $MgSO_4$, filtered, and concentrated *in vacuo*. The crude residue was purified by flash column chromatography (EtOAc/Hex = 1/9 → 1/5) on silica gel to give the product **21** (4.5 g, 77%). ^{1}H NMR (600 MHz, $CDCl_3$) δ 8.18-8.17 (m, 2H, Ar-H), 7.89-7.87 (m, 1H, Ar-H), 7.82-7.80 (m, 2H, Ar-H), 7.73-7.69 (m, 4H, Ar-H), 7.64 (bs, 1H, Ar-H), 7.55-7.53 (m, 2H, Ar-H), 7.45-7.27 (m, 26H, Ar-H), 7.19 (s, 1H, Ar-H), 7.05 (d, *J* = 8.2 Hz, 2H, Ar-H), 5.23-5.18 (m, 3H, H-2, $ArCH_2$) 5.0-4.88 (m, 4H, H-1, $ArCH_2$), 4.79-4.76 (m, 2H, H-1', $ArCH_2$), 4.53-4.48 (m, 3H, $ArCH_2$), 4.46-4.43 (m, 1H, H-6a), 4.40 (bs, 1H, H-5), 4.23 (d, *J* = 9.8 Hz, 1H, $ArCH_2$), 4.17 (dd, *J* = 4.5, 11.1 Hz, 1H, H-6b), 4.13-4.12 (m, 1H, H-3), 4.08-4.06 (m, 1H, H-6'a), 3.94 (d, *J* = 11.4 Hz, 1H, H-6'b), 3.87-3.72 (m, 5H, H-3', H-4', H-5', H-4, linker CH_2), 3.47-3.41 (m, 1H, linker CH_2), 3.37 (dd, *J* = 10.1, 3.4 Hz, 1H, H-2'), 3.28-3.18 (m, 2H, linker CH_2), 2.00-1.95 (m, 3H, OAc), 1.66-1.53 (m, 4H, linker CH_2), 1.36-1.31 (m, 2H, linker CH_2), 1.11 (s, 9H, TBDPS); ^{13}C NMR

(150 MHz, $CDCl_3$) δ 170.4 (C), 165.7 (C), 156.7 (C), 156.2 (C), 138.0 (C), 137.8 (C), 136.7 (C), 135.9 (CH), 135.6 (CH), 135.4 (C),133.5 (C), 133.3 (C), 133.0 (CH), 132.9 (C), 132.87 (C), 131.5 (CH), 129.9 (CH), 129.8 (CH), 129.7 (CH), 129.6 (CH), 128.6 (CH), 128.5 (CH), 128.4 (CH), 128.3 (CH), 128.1 (CH), 128.0 (CH), 127.9 (CH), 127.8 (CH), 127.7 (CH), 127.2 (CH), 127.1 (CH), 127.0 (CH), 126.4 (CH), 126.2 (CH), 126.1 (CH), 125.7 (CH), 125.5 (CH), 121.6 (C), 98.4 (CH), 98.2 (CH), 80.7 (CH), 80.0 (CH), 77.9 (CH), 75.2 (CH_2), 74.4 (CH), 73.1 (CH_2), 73.0 (CH), 72.5 (CH), 72.3 (CH_2), 69.1 (CH), 68.0 (CH), 67.2 (CH_2), 67.1 (CH_2), 65.6 (CH), 64.0 (CH), 62.3 (CH_2), 62.1 (CH_2), 50.5 (CH_2), 50.2 (CH_2), 47.2 (CH_2), 46.2 (CH_2), 29.1 (CH_2), 26.9 (CH_3), 23.5 (CH_2) 19.4 (C); HRMS m/z (ESI, MNa^+) calcd for $C_{82}H_{87}N_4O_{14}SiBrNa^+$ 1481.5069, found 1481.5057.

Compound 22.

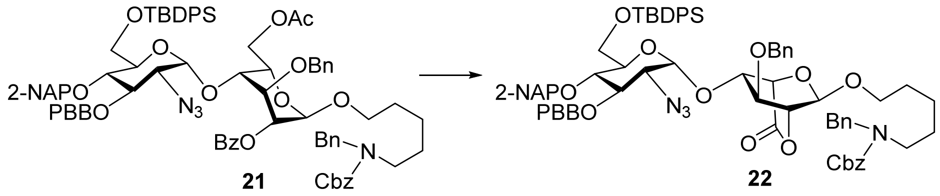

To a solution of compound **21** (4.4 g, 3.0 mmol) in a 1/1 ratio of methanol/dichloromethane (20 mL) was added sodium methoxide (24 mg, 0.45 mmol) at 0 °C under nitrogen atmosphere. The ice bath was removed, and the resulting solution was kept stirring at room temperature for 18 h. The reaction mixture was neutralized by DOWEX 50 (H^+ exchange resin), filtered, and concentrated *in vacuo*. The crude 2,6-diol compound (3.8 g, 2.9 mmol) was dissolved in a 2/1 ratio of dichloromethane/water (45 mL) at room temperature. (2,2,6,6-Tetramethylpiperidin-1-yl)oxyl (TEMPO, 91 mg, 0.58 mmol) and bis(acetoxy)iodobenzene (2.3 g, 7.1 mmol) were consecutively added to the solution, and the mixture was allowed to stir at the same temperature for 16 h. The reaction was quenched by 10% $Na_2S_2O_{3(aq)}$, and the mixture was diluted with dichloromethane. The organic layer was washed with water, dried over anhydrous $MgSO_4$, filtered, and concentrated *in vacuo*. The crude residue was purified by flash column chromatography (EtOAc/Hex = 1/20 → 1/5) on silica gel to furnish the lactone **22** (2.6 g, 67% in two steps). ^1H NMR (600 MHz, $CDCl_3$) δ 7.86-7.85 (m, 1H, Ar-H), 7.80-7.73 (m, 4H, Ar-H), 7.69-7.65 (m, 3H, Ar-H), 7.53-7.50 (m, 2H, Ar-H), 7.46-7.45 (m, 2H, Ar-H), 7.43-7.28 (m, 21H, Ar-H), 7.22-7.19

(m, 3H, Ar-H), 5.23-5.20 (m, 2H, ArCH2) 5.13-5.05 (m, 2H, H-1, H-1'), 4.98-4.82 (m, 4H, ArCH2), 4.76-4.70 (m, 2H, ArCH2), 4.60-4.50 (m, 3H, H-2, ArCH2), 4.42 (d, *J* = 3.1 Hz, 1H, H-5), 4.27-4.24 (m, 1H, H-4), 4.04-4.00 (m, 2H, H-6'), 3.97-3.91 (m, 2H, H-3, H-3'), 3.87-3.83 (m, 3H, H-4', H-5', linker CH2), 3.50-3.37 (m, 2H, H-2', linker), 3.29.-3.20 (m, 2H, linker), 1.67-1.52 (m, 4H, linker CH2), 1.38-1.30 (m, 2H, linker CH2), 1.11 (s, 9H, TBDPS); ^{13}C NMR (150 MHz, $CDCl_3$) δ 167.6 (C),156.6 (C), 156.1 (C), 137.8 (C), 137.2 (C), 136.7 (C), 135.8 (CH), 135.5 (CH), 135.2 (C), 133.3 (C), 133.1 (C),132.9 (C), 132.8 (C), 131.4 (CH), 129.6 (CH), 129.5 (CH), 128.6 (CH), 128.4 (CH), 128.3 (CH), 128.1 (CH), 127.9 (CH), 127.8 (CH), 127.7 (CH), 127.6 (CH), 127.4 (CH), 127.2 (CH), 127.0 (CH), 126.0 (CH), 125.8 (CH), 125.4 (CH), 121.7 (C), 99.7 (CH), 96.8 (CH), 80.3 (CH), 79.6 (CH), 79.4 (CH), 78.0 (CH), 74.9 (CH2), 74.5 (CH2), 72.6 (CH), 72.1(CH), 72.0 (CH2), 69.4 (CH2), 69.3 (CH), 67.1 (CH2), 63.2 (CH), 62.1 (CH2), 50.4 (CH2), 50.1 (CH2), 47.0 (CH2), 46.0 (CH2), 29.0 (CH2), 26.8 (CH3), 19.2 (C); HRMS m/z (ESI, M+Na$^+$) calcd for $C_{73}H_{77}N_4O_{12}SiBrNa^+$ 1331.4388, found 1331.4397.

Compound 23.

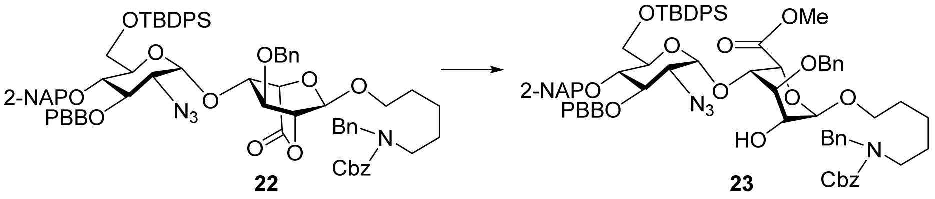

Compound **22** (10 g, 7.63 mmol) was dissolved in a 2/1 ration of dichloromethane/methanol (150 mL), triethylamine (6.5 mL) was added to the solution, and the mixture was stirred at 40 °C for 18 h. The reaction flask was cooled down to room temperature, and the resulting solution was concentrated *in vacuo*. The crude residue was purified by flash column chromatography (EtOAc/Hex = 1/9 → 1/1) on silica gel to provide the 2-alcohol **23** (8.5 g, 83 %). ^{1}H NMR (600 MHz, CD_3OD) δ 7.76-7.75 (m, 1H, Ar-H), 7.66-7.64 (m, 2H, Ar-H), 7.59 (d, *J* = 6.9 Hz, 2H, Ar-H), 7.54 (d, *J* = 7.2 Hz, 2H, Ar-H), 7.5 (s, 1H, Ar-H), 7.42-7.40 (m, 2H, Ar-H), 7.34-7.33 (m, 2H, Ar-H), 7.30-7.21 (m, 23H, Ar-H), 7.06 (s, 1H, Ar-H), 5.13 (d, *J* = 3.4 Hz, 1H, H-1'), 5.04(d, *J* = 18.3 Hz, 2H, ArCH2), 4.89-4.86 (m, 2H, H-1, ArCH2), 4.76-4.71 (m, 6H, ArCH2), 4.59 (d, *J* = 11.1 Hz, 1H, ArCH2), 4.37 (s, 2H, H-2, H-5), 4.04 (m, 1H, H-3), 3.88 (t, *J* = 5.1 Hz, 1H, H-4),

3.82-3.78 (m, 3H, H-3', H-6'), 3.65-3.60 (m, 6H, H-4', H-5' OCH_3, linker CH_2), 3.45-3.38 (m, 2H, H-2', linker CH_2), 3.15-3.11 (m, 2H, linker CH_2), 1.54-1.45 (m, 4H, linker CH_2), 1.271.22 (m, 2H, linker CH_2), 0.95 (s, 9H, TBDPS); ^{13}C NMR (150 MHz, CD_3OD) δ 171.7 (C), 139.8 (C), 138.8 (C), 137.1 (CH), 136.9 (CH), 134.8 (C), 134.7 (C), 134.6 (C), 134.5 (C), 132.6 (C), 131.0 (CH), 129.7 (CH), 129.5 (CH), 129.4 (CH), 129.1 (CH), 129.0 (CH), 128.9 (CH), 128.5 (CH), 127.4 (CH), 127.2 (CH), 126.8 (CH), 122.7 (C), 102.9 (CH), 97.8 (CH), 81.8 (CH), 79.6 (CH), 79.5 (CH), 77.0 (CH), 76.2 (CH_2), 75.5 (CH_2), 74.3 (CH_2), 74.2 (CH), 74.0 (CH_2), 70.8 (CH), 70.7 (CH_2), 69.9 (CH), 68.5 (CH), 65.2 (CH), 64.2 (CH_2), 52.9 (CH), 27.6 (CH_3), 20.3 (C); HRMS m/z (ESI, M+Na$^+$) calcd for $C_{74}H_{81}N_4O_{13}SiBrNa^+$ 1363.4650, found 1363.4653.

Compound 24.

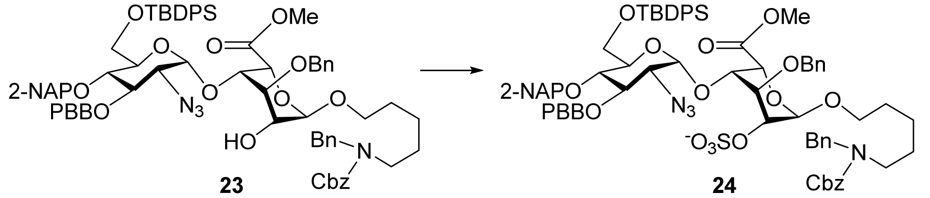

To a solution of compound **23** (10.5 g, 7.82 mmol) in *N*,*N*-dimethylformamide (100 mL) was added sulfur trioxide/triethylamine (8.5 g, 46.9 mmol) at room temperature. The reaction flask was heated to 60 °C, and the mixture was kept stirring at the same temperature for 18 h. The reaction flask was cooled down to room temperature, water (100 mL) and sodium bicarbonate (20.0 g) were consecutively added to the solution, and the mixture was continuously stirred for 3 h. The resulting mixture was concentrated *in vacuo*, and the crude mass was extracted by dichloromethane (2 x 150 mL). The combined organic layer was washed with sat. $NaHCO_{3(aq)}$, dried over anhydrous $MgSO_4$, filtered, and concentrated *in vacuo*. The crude residue was purified by flash column chromatography (MeOH/$CHCl_3$ = 1/20 → 1/15 → 1/10 → 1/5) on silica gel to yield the 2-sulfate **24** (8.3 g, 75%). ^{1}H NMR (600 MHz, CD_3OD) δ 7.72-7.71 (m, 1H, Ar-H), 7.63-7.61 (m, 4H, Ar-H), 7.54 (d, *J* = 7.6 Hz, 2H, Ar-H), 7.48 (s, 1H, Ar-H), 7.37-7.35 (m, 2H, Ar-H), 7.29-7.28 (m, 2H, Ar-H), 7.24-7.16 (m, 23H, Ar-H), 7.04-7.02 (m, 1H, Ar-H), 5.17 (d, *J* = 3.0 Hz, 1H, H-1'), 5.09 (s, 1H, H-1), 5.01(d, *J*= 25.5 Hz, 2H, $ArCH_2$), 4.84-4.81 (m, 2H, $ArCH_2$), 4.75-4.72 (m, 4H, H-5, $ArCH_2$), 4.51-4.48 (m, 2H, H-2, $ArCH_2$), 4.34-4.31 (m, 3H, H-3, $ArCH_2$), 4.11 (s, 1H, H-

4),3.92-3.90 (m, 1H, H-5'), 3.86-3.82 (m, 2H, H-6'), 3.71-3.69 (m, 2H, H-3', H-4'), 3.62-3.55 (m, 4H, linker, CO_2Me), 3.41-3.36 (m, 1H, linker CH_2),3.25-3.24 (m, 1H, H-2'), 3.08-3.05 (m, 2H, linker CH_2), 1.51-1.40 (m, 4H, linker CH_2), 1.24-1.20 (m, 1H, linker CH_2), 1.13-1.10 (m, 1H, linker CH_2), 0.96 (s, 9H, TBDPS); ^{13}C NMR (150 MHz, CD_3OD) δ 171.7 (C), 158.5 (C), 157.9 (C), 139.5 (C), 139.1 (C), 138.2 (C), 137.2 (C), 137.1 (CH), 136.8 (CH), 134.79 (C), 134.72 (C), 134.5 (C), 134.4 (C), 132.5 (CH), 131.08 (CH), 131.05 (CH), 131.0 (CH), 129.7 (CH), 129.6 (CH), 129.3 (CH),129.2 (CH), 129.1 (CH), 129.0 (CH), 128.9 (CH), 128.7 (CH), 128.5 (CH), 128.4 (CH), 127.3 (CH), 127.2 (CH), 127.1 (CH), 126.7 (CH), 122.5 (C), 100.9 (CH), 97.5 (CH), 81.4 (CH), 79.6 (CH), 79.5 (CH), 76.0 (CH_2), 75.3 (CH_2), 74.1 (CH), 73.19 (CH_2), 73.12 (CH), 72.5 (CH), 72.4 (CH), 69.5 (CH_2), 69.4 (CH_2), 68.7 (CH), 68.5 (CH_2), 68.4 (CH_2), 65.2 (CH), 64.1 (CH_2), 52.9 (CH), 51.6 (CH_2), 51.4 (CH_2), 48.5 (CH_2), 48.0 (CH_2), 47.6 (CH), 30.3 (CH_2),29.0 (CH_2), 28.6 (CH_2), 27.6 (CH_3), 24.6 (CH), 24.6 (CH), 20.2 (C), 9.4 (CH_3); HRMS m/z (ESI, $(M-H)^-$) calcd for $C_{74}H_{80}N_4O_{16}SiSBr^-$ 1419.4243, found 1419.4247.

Compound 25.

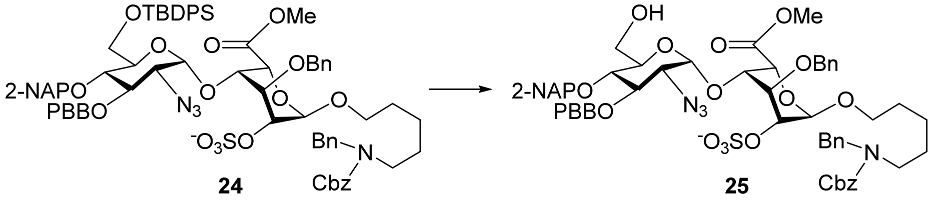

A 7/3 ratio of hydrogen fluoride/pyridine (2 mL) was slowly added to a solution of compound **24** (2.0 g, 1.4 mmol) in tetrahydrofuran (8 ml) and pyridine (8 ml) at 0 °C under nitrogen atmosphere. The reaction solution was stirred for 3 d, silica gel (10 g) was added to quench the reaction, and the mixture was kept stirring for 1 h. The whole mixture was filtered, and the solid was washed with a 1/1 ratio of chloroform/methanol (20 mL). The filtrate was concentrated *in vacuo*, and the crude residue was purified by flash column chromatography (MeOH/$CHCl_3$ = 1/20 → 1/15 → 1/10 → 1/4) on silica gel to furnish the 6'-alcohol **25** (1.0 g, 60%). ^{1}H NMR (600 MHz, CD_3OD) δ 7.84-7.79 (m, 3H, Ar-H), 7.72 (m, 1H, Ar-H), 7.49-7.46 (m, 2H, Ar-H), 7.32-7.15 (m, 22H, Ar-H), 5.19-5.12 (m, 4H, H-1, H-1', $ArCH_2$), 4.92-4.88 (m, 2H, $ArCH_2$), 4.84-4.79 (m, 4H, H-5, $ArCH_2$), 4.64 (d, J = 11.4 Hz, 1H, $ArCH_2$), 4.54 (s, 1H, H-2), 4.47-4.56 (m, 2H, $ArCH_2$),

4.36 (bs, 1H, H-3), 4.18 (bs, 1H, H-3'), 4.03-4.00 (m, 1H, H-4), 3.83-3.74 (m, 6H, H-5', H-6', CO_2Me), 3.72-3.63 (m, 2H, H-4', linker CH_2), 3.51-3.44 (m, 1H, linker CH_2), 3.26-3.25 (m, 1H, H-2'), 3.21 (bs, 2H, linker CH_2), 1.53-1.46 (m, 4H, linker CH_2), 1.36-1.30 (m, 2H, linker CH_2); ^{13}C NMR (150 MHz, CD_3OD) δ 171.8 (C), 158.5 (C), 157.9 (C), 139.5 (C), 139.2 (C), 138.1 (C), 137.4 (C), 134.8 (C), 134.4 (C), 132.4 (CH), 130.8 (CH), 129.6 (CH), 129.5 (CH), 129.3 (CH), 129.2 (CH), 129.2 (CH), 129.1 (CH) 128.9 (CH), 128.7 (CH) 128.4 (CH), 128.3 (CH), 127.2 (C), 127.23 (C), 127.0 (C), 126.8(C), 122.3 (C), 101.0 (CH), 98.2 (CH), 81.1 (CH), 79.2 (CH), 75.7 (CH_2), 75.2 (CH_2), 73.9 (CH), 73.7 (CH), 73.1 (CH), 73.0 (CH_2), 72.6 (CH), 72.6 (CH), 69.4 (CH_2), 69.3 (CH_2), 68.6 (CH), 68.5 (CH_2), 68.3 (CH_2), 65.0 (CH), 61.6 (CH_2), 52.9 (CH), 51.5 (CH_2), 51.3 (CH_2), 48.4 (CH), 48..0 (CH_2), 47.6 (CH), 30.2 (CH_3),29.0 (CH_2), 28.5 (CH_2), 24.6 (CH_2), 24.5 (CH_2), 9.3 (CH_3); HRMS m/z (ESI, M-H+2Na$^+$) calcd for $C_{58}H_{62}N_4O_{16}Na_2SBr^+$ 1227.2860, found 1227.2854.

Compound 26.

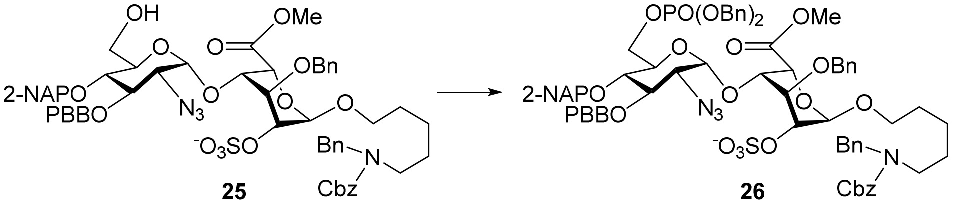

Compound **25** (300 mg, 0.25 mmol) was dissolved in a 1/9 ratio of *N*,*N*-dimethylformamide/ dichloromethane (20 mL) at room temperature under nitrogen atmosphere. $(BnO)_2PN(i\text{-}Pr)_2$ (0.54 mL, 1.56 mmol) and 1H-tetrazole (180 mg, 2.5 mmol) were sequentially added to the solution, and the mixture was continuously stirred for 2 h. The reaction flask was immersed in an ice bath, *m*-chloroperbenzoic acid (360 mg, 2.0 mmol) was added to the reaction solution, and the whole mixture was kept stirring for another 18 h. The reaction was quenched by 10% $Na_2S_2O_3$ (20 mL) and neutralized by sat. $NaHCO_{3(aq)}$ (20 mL). The aqueous layer was extracted with dichloromethane (2 x 20 mL), and the combined organic layers were washed with water (20 mL), dried over anhydrous $MgSO_4$, filtered, and concentrated *in vacuo*. The residue was purified by flash column chromatography ($MeOH/CHCl_3$ = 1/10 → 1/7 → 1/5) on silica gel to afford the dibenzyl phosphate **26** (238 mg, 65%). ^1H NMR (600 MHz, CD_3OD) δ 7.70-7.66 (m, 1H, Ar-H),

7.64-7.61 (m, 2H, Ar-H), 7.52 (s, 1H, Ar-H), 7.36-7.31(m, 2H, Ar-H), 7.27-7.24 (m, 2H, Ar-H), 7.24-7.21 (m, 4H, Ar-H), 7.18-7.13 (m, 15H, Ar-H), 7.12-7.05 (m, 8H, Ar-H), 7.04-7.00 (m, 1H, Ar-H), 5.09-5.04 (m, 1H, H-1), 5.04-4.97 (m, 2H, $ArCH_2$), 4.95 (d, *J* = 2.8 Hz, 1H, H-1'), 4.87 (d, *J* = 8.5Hz, 4H, $ArCH_2$), 4.77-4.74 (m, 1H, $ArCH_2$), 4.71-4.63 (m, 4H, H-5, $ArCH_2$), 4.60 (d, *J* = 11.5 Hz, 1H, ArCH2), 4.46 (d, *J* = 11.5Hz, 1H, ArCH2), 4.41 (s, 1H, H-2), 4.32(d, *J* = 19.2 Hz, 2H, $ArCH_2$), 4.20 (s, 1H, H-3), 4.19-4.14 (m, 1H, H-6'a), 4.11-4.06 (m, 1H, H-6'b), 4.01 (s, 1H, H-4), 3.89 (t, *J* = 19 Hz, 1H, H-3'), 3.77 (d, *J* = 9.8 Hz, 1H, H-5'), 3.61-3.48 (m, 4H, linker CH_2, CO_2CH_3), 3.42-3.27 (m, 2H, H-4', linker CH_2), 3.10 (dd, *J* = 10.2, 3.2 Hz, 1H, H-2'), 3.08-2.98 (m, 2H, linker CH_2), 1.48-1.29 (m, 4H, linker CH_2), 1.20-1.07 (m, 2H, linker CH_2); ^{13}C NMR (150 MHz, CD_3OD) δ 171.8(C), 158.4(C), 157.9(C), 139.4(C), 139.2(C), 139.1(C), 138.1(C), 137.1(C), 137.1(C), 137.1(C), 137.1(C), 137.0(C), 134.7(C), 134.4(C), 132.4(CH), 130.8(CH), 129.7(CH), 129.6(CH), 129.5(CH), 129.3(CH), 129.2(CH), 129.1(CH), 129.0(CH), 129.0(CH), 128.8(CH), 128.7(CH), 128.4(CH), 128.3(CH), 127.3(CH), 127.2(CH), 127.0(CH), 126.8(CH), 122.3(C), 101.0(CH), 98.5(CH), 81.2(CH), 78.5(CH), 75.6(CH_2), 75.3(CH_2), 74.0(CH), 73.6(CH), 72.9(CH_2), 72.0(CH), 72.0(CH), 70.9(CH_2), 70.8(CH_2), 70.8(CH_2), 69.3(CH_2), 68.5(CH_2), 68.3(CH_2), 68.2(CH_2), 67.7(CH_2), 67.7(CH_2),64.9(CH), 53.1(CH_3), 51.5(CH_2), 51.3(CH_2), 48.4(CH_2), 48.4(CH_2), 47.5(CH_2), 30.2(CH_2), 29.0(CH_2), 28.5(CH), 24.6(CH_2), 24.5(CH_2), 19.4(CH_3); HRMS m/z (ESI, $M+Na^+$) calcd for $C_{72}H_{75}N_4O_{19}Na_2PSBr$ 1487.3463, found 1487.3469.

Compound 27.

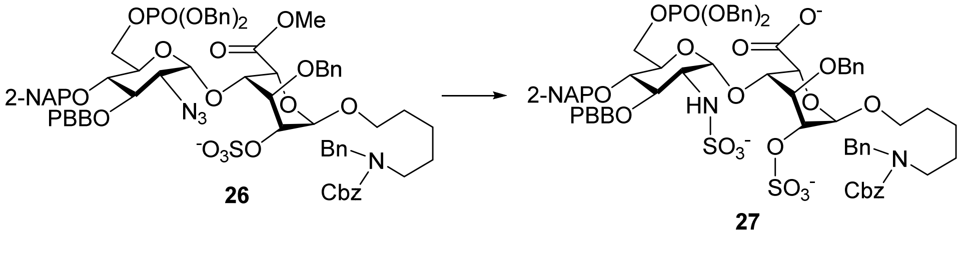

To a solution of compound **26** (293 mg, 0.2 mmol) in tetrahydrofuran (2.5 mL) were consecutively added a 1.0 N solution of lithium hydroxide in water (5.0 mL) and a 37% solution of hydrogen peroxide in water (1 mL) at room temperature. The mixture was stirred at 37 °C for 18 h, then the reaction flask was cooled down to room temperature. The reaction was quenched and neutralized

with acetic acid till pH 7, and the resulting mixture was concentrated *in vacuo*. The crude compound was dissolved in tetrahydrofuran (5 mL) at room temperature, a 0.1 N sodium hydroxide aqueous solution (0.2 mL) and a 1 M solution of trimethylphosphine in tetrahydrofuran (0.2 mL, 0.2 mmol) were sequentially added into the reaction flask, and the mixture was continuously stirred for 14 h. The reaction was quenched by 0.1 N $HCl_{(aq)}$ (0.2 mL), the mixture was concentrated *in vacuo*, and the residue was purified by flash column chromatography ($MeOH/CHCl_3$ = 1/9 → 1/4) on silica gel followed by Na^+ ion exchange column (AG 50W-X8 cation exchange resin) using methanol as eluent to obtain the crude 2'-amino product. This amine compound was dissolved in methanol (3 mL) at room temperature. A solution of 0.1 N sodium hydroxide aqueous solution (2.8 mL, 0.28 mmol), triethylamine (2.8 mL, 20 mmol) and sulfur trioxide/pyridine complex (318 mg, 2 mmol) were consecutively added to the solution, and the reaction flask was kept stirring for 18 h. The mixture was concentrated under the reduced pressure, and the crude residue was purified by flash column chromatography ($MeOH/CHCl_3$ = 1/20 → 1/15 → 1/10 → 1/5) on silica gel to provide the *N*-sulfate **27** (174 mg, 56% in 3 steps). ^1H NMR (600 MHz, CD_3OD) δ 7.70-7.68 (m, 1H, Ar-H), 7.65-7.61 (m, 2H, Ar-H), 7.49 (s, 1H, Ar-H), 7.37-7.32 (m, 2H, Ar-H), 7.28-7.08 (m, 29H, Ar-H), 7.08-7.03 (m, 2H, Ar-H), 5.31(s, 1H, H-1'), 5.19 (s, 1H, H-1), 5.06-5.00 (m, 3H, $ArCH_2$), 4.98-4.86 (m, 4H, $ArCH_2$), 4.73-4.71 (m, 1H, $ArCH_2$), 4.66-4.55 (m, 5H, H-5, H-6', $ArCH_2$), 4.45 (s, 1H, H-2), 4.37-4.23 (m, 5H, H-3, $ArCH_2$), 4.14 (s, 1H, H-4), 3.94 (d, *J* = 9.4 Hz, 1H, H-5'), 3.71 (t, *J* = 7.9 Hz, 1H, H-4'), 3.59-3.51(m, 1H, linker CH_2), 3.49-3.43 (m, 2H, H-2', H-3'), 3.39-3.27 (m, 1H, linker CH_2), 3.06-2.96 (m, 1H, linker CH_2), 1.47-1.30 (m, 4H, linker CH_2), 1.10-1.05 (m, 2H, linker CH_2); ^{13}C NMR (150 MHz, CD_3OD) δ 175.8(C), 158.5(C), 157.9(C), 140.2(C), 139.9(C), 139.3(C), 139.2(C), 138.2(C), 137.4(C), 137.2(C), 137.1(C), 134.7(C), 134.4(C), 132.2(CH), 131.2(CH), 129.8(CH), 129.7(CH), 129.6(CH), 129.5(CH), 129.2(CH), 129.2(CH), 129.0(CH), 129.0(CH), 128.8(CH), 128.7(CH), 128.5(CH), 128.3(CH), 127.5(CH), 127.3(CH), 127.2(CH), 127.1(CH), 126.9(CH), 122.0(C), 100.7(CH), 100.4(CH), 81.7(CH), 78.2(CH), 76.6(CH), 75.8(CH_2), 75.7(CH_2), 75.4(CH_2), 72.9(CH_2), 72.2465, 71.1(CH_2), 71.0(CH_2), 70.9(CH_2), 70.8(CH), 69.1(CH_2), 69.0(CH_2), 68.5(CH_2), 68.4(CH_2), 68.1(CH_2), 68.0(CH_2), 60.3(CH), 55.2(CH), 51.5(CH), 51.3(CH), 49.6(CH), 49.5(CH), 48.6(CH), 48.5(CH), 48.0(CH), 47.9(CH), 47.8(CH), 47.6(CH), 44.4(CH), 31.9(CH), 30.4(CH_2), 30.2(CH_2), 29.1(CH_2), 28.6(CH_2), 27.5(CH), 24.6(CH_2), 21.4(CH), 14.3(CH), 14.0(CH), 9.3(CH_3); HRMS m/z (ESI, $(M-3Na+2H)^-$) calcd for $C_{71}H_{75}N_2O_{22}PS_2Br^-$ 1481.3174, found 1481.3149.

Compound 28.

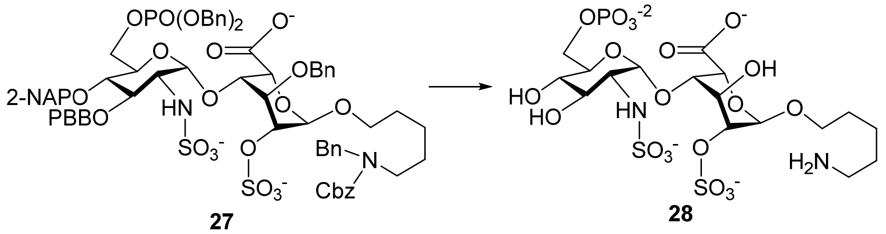

A mixture of compound **27** (10.3 mg, 6.7 μmol) and Degussa type 20% $Pd(OH)_2/C$ (78 mg) in methanol (2 mL) and pH 7 phosphate buffer (0.15 mL) was purged with nitrogen for 10 min at room temperature. The reaction flask was then equipped with a hydrogen balloon. After stirring for 3 d, the whole mixture was filtered through a pad of Celite®, washed with methanol, and concentrated *in vacuo*. The residue was purified by Sephadex G-25 column followed by AG 50W-X8 cation exchange column (Na^+ form) using water as eluent to furnish the disaccharide 6'-phpsphate **28** (4.1 mg, 78%). ^{1}H NMR (600 MHz, D_2O) δ 5.44 (s, 1H, H-1'), 5.21 (s, 1H, H-1), 4.57 (s, 1H, H-5), 4.32-4.28 (m, 2H, H-2, H-3), 4.21-4.13 (m, 2H, H-4, H-6'a), 3.98-3.95 (m, 1H, H-6'b), 3.92-3.90 (m, 1H, H-5'), 3.85-3.82 (m, 1H, linker CH_2), 3.77-3.71 (m, 3H, H-3', H-4'), 3.36-3.34 (m, 1H, H-2'), 3.10-3.06 (m, 2H, linker CH_2), 1.79-1.69 (m, 4H, linker CH_2), 1.56-1.53 (m, 2H, linker CH_2); ^{13}C NMR (150 MHz, D_2O) δ 173.3(CH), 97.3(CH), 95.5(CH), 76.0(CH), 75.6(CH), 71.5(CH), 71.4(CH), 70.6(CH), 69.0(CH), 68.5(CH), 68.4(CH), 68.0(CH_2), 67.9(CH), 62.3(CH_2), 58.1(CH), 39.3(CH_2), 27.8(CH_2), 26.1(CH_2), 22.2(CH_2); HRMS m/z (ESI, (M-3Na+2H)$^-$) calcd for $C_{17}H_{30}N_2O_{20}Na_2PS_2^-$ 723.0366, found 723.0362.

Compound 29.

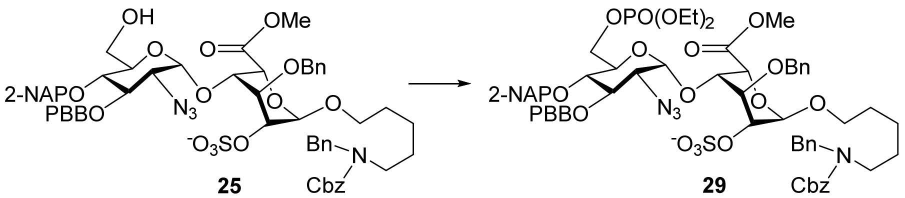

Compound **25** (107 mg, 90.4 μmol) was dissolved in pyridine (2 mL) at room temperature under nitrogen atmosphere. Diethyl phosphoryl chloride (76 μL, 525 μmol) was added to the solution, and the reaction mixture was kept stirring at the same temperature for 2 h. The resulting solution

was diluted with dichloromethane (10 mL), and the mixture was quenched with sat. sodium bicarbonate. The organic layer was washed with water, dried over $MgSO_4$, filtered, and concentrated *in vacuo*. The crude residue was purified by flash column chromatography MeOH/$CHCl_3$ = 1/20 → 1/15 → 1/10 → 1/7 → 1/5) on silica gel to afford compound **29** (106 mg, 89%). ^{1}H NMR (600 MHz, CD_3OD) δ 7.85-7.81 (m, 3H, Ar-H), 7.73 (bs, 1H, Ar-H), 7.50-7.46 (m, 2H, Ar-H), 7.42-7.37 (m, 7H, Ar-H), 7.32-7.21(m, 12H, Ar-H), 7.17-7.15 (m, 1H, Ar-H), 5.20-5.13 (m, 4H, H-1', H-1, $ArCH_2$), 4.96-4.91 (m, 2H, $ArCH_2$), 4.84-4.80 (m, 4H, H-5, $ArCH_2$), 4.64-4.62 (m, 1H, $ArCH_2$), 4.55 (bs, 1H, H-2), 4.47-4.45 (m, 2H, $ArCH_2$), 4.35 (bs, 1H, H-3), 4.30-4.27 (m, 1H, H-6'a), 4.25-4.23 (m, 1H, H-6'b), 4.17 (bs, 1H, H-4), 4.10-4.02 (m, 5H, H-3', OEt), 3.97-3.94 (m, 1H, H-5'), 3.79 (s, 3H, OCH_3), 3.72-3.62 (m, 2H, H-4', linker CH_2), 3.52-3.43 (m, 1H linker CH_2), 3.37 (dd, J = 10.2, 3.0, 1H, H-2'), 3.32-3.16 (m, 2H, linker CH_2), 1.62-1.46 (m, 4H, linker CH_2), 1.19-1.12 (m, 8H, OEt, linker CH_2); ^{13}C NMR (150 MHz, CD_3OD) δ 170.4 (C), 157.0 (C), 156.5 (C), 148.6 (CH), 138.0 (C), 137.8 (C), 136.7 (C), 135.6 (C), 133.3 (C), 133.0 (C), 131.0 (CH), 129.5 (CH), 128.2 (CH), 128.1 (CH), 127.9 (CH), 127.8 (CH),127.7 (CH) 127.6 (CH), 127.5 (CH), 127.4 (CH), 127.3 (CH), 127.0 (CH), 126.0 (CH), 125.9 (CH), 125.6 (CH), 125.4 (CH) 120.9 (C), 99.6 (CH), 97.1 (CH), 79..8 (CH), 77.4 (CH), 74.3 (CH_2), 73.9 (CH_2), 72.6 (CH), 71.5 (CH_2), 70.8 (CH), 70.6 (CH), 67.9 (CH_2), 67.0 (CH_2), 66..9 (CH_2), 66.8 (CH_2), 66.0 (CH_2), 65.9 (CH_2), 64.2 (CH_2), 64.1 (CH_2), 63.7 (CH), 51.5 (CH_2), 50.1 (CH_2), 49.9 (CH_2), 48.5 (CH), 47.0 (CH_2), 46.5 (CH_2), 46.2 (CH_2), 28.8 (CH_2), 27.5 (CH_2), 27.1 (CH_2), 23.2 (CH_2), 23.1 (CH_2), 17.0 (CH_3), 15.1 (CH_3), 7.9 (CH_3) ; HRMS m/z (ESI, $(M-H)^-$) calcd for $C_{62}H_{71}N_4O_{19}PSBr^-$ 1317.3354, found 1317.3334.

Compound 30.

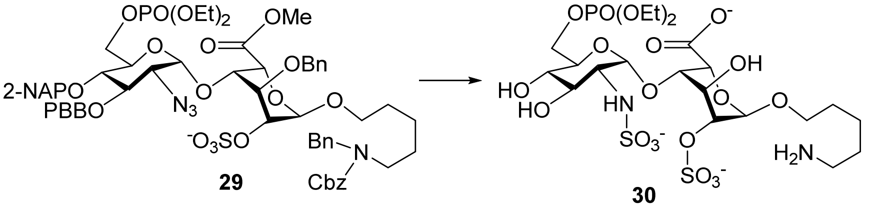

Compound **29** (98 mg, 74 μmol) was dissolved in tetrahydrofuran (1.0 mL) at room temperature. A 1.0 N solution lithium hydroxide in water (1.9 mL) and a 37% solution of hydrogen peroxide in

water (0.37 mL) were sequentially added to the reaction solution. The mixture was warmed up and stirred at 37 °C for 18 h. After cooling to room temperature, the reaction was neutralized by acetic acid till pH 7, and the resulting mixture was concentrated *in vacuo*. The crude compound was dissolved in tetrahydrofuran (1 mL) at room temperature, a 0.1 N sodium hydroxide aqueous solution (74 μL) and a 1 M solution of trimethylphosphine in tetrahydrofuran (74 μL, 74 μmol) were sequentially added into the reaction flask, and the mixture was continuously stirred for 14 h. The reaction was quenched by 0.1 N $HCl_{(aq)}$ (74 μL), the mixture was concentrated *in vacuo*, and the residue was purified by flash column chromatography (MeOH/$CHCl_3$ = 1/9 → 1/4) on silica gel followed by Na^+ ion exchange column (AG 50W-X8 cation exchange resin) using methanol as eluent to obtain the crude 2'-amino product. This amine compound was dissolved in methanol (1 mL) at room temperature. A solution of 0.1 N sodium hydroxide aqueous solution (1.0 mL, 0.1 mmol), triethylamine (1.0 mL, 7.4 mmol) and sulfur trioxide/pyridine complex (118 mg, 0.74 mmol) were consecutively added to the solution, and the reaction flask was kept stirring for 18 h. The mixture was concentrated under the reduced pressure, and the crude residue was purified by flash column chromatography (MeOH/$CHCl_3$ = 1/20 → 1/15 → 1/10 → 1/5) on silica gel to give the *N*-sulfate. ^{1}H NMR (600 MHz, CD_3OD) δ 7.76-7.71 (m, 3H, Ar-H), 7.57 (s, 1H, Ar-H), 7.40-7.38 (m, 2H, Ar-H), 7.31-7.17 (m, 20H, Ar-H), 5.32 (d, *J* = 3.4 Hz, 1H, H-1'), 5.19 (s, 1H, H-1), 5.10-5.05 (m, 3H, $ArCH_2$), 4.88-4.85 (m, 1H, $ArCH_2$), 4.74-4.71 (m, 2H, $ArCH_2$), 4.67-4.62 (m, 3H, H-5, $ArCH_2$), 4.48 (s, 1H, H-2), 4.40-4.36 (m, 3H, H-3, $ArCH_2$), 4.31-4.26 (m, 2H, H-6'), 4.08 (s, 1H, H-4), 4.05-3.99 (m, 4H, OEt), 3.89 (d, *J* = 9.9 Hz, 1H, H-5'), 3.79-3.76 (m, 1H, H-4'), 3.63-3.56 (m, 2H, H-3', linker CH_2), 3.44 (dd, *J* = 10.3, 3.4, Hz, 1H, H-2'), 3.43-3.37 (m, 1H, linker CH_2), 3.12-3.06 (m, 2H, linker CH_2), 1.54-1.43 (m, 4H, linker CH_2), 1.26-1.18 (m, 8H, OEt, linker CH_2); ^{13}C NMR (150 MHz, CD_3OD) δ 174.2 (C), 158.6 (C), 158.0 (C), 140.2 (C), 139.9 (C), 139.5 (C), 139.4 (C), 138.2(C), 137.6 (C), 134.9 (C), 134.5 (C), 132.3 (CH), 131.5 (CH), 129.7 (CH), 129.6 (CH), 129.3 (CH), 129.2 (CH), 129.1 (CH), 129.0 (CH), 128.8 (CH), 128.7 (CH), 128.6 (CH), 128.5 (CH), 128.4 (CH), 127.4 (CH), 127.2 (CH), 127.0 (CH), 127.0 (CH), 122.1 (C), 101.1 (CH), 100.6 (CH), 81.8 (CH), 78.4 (CH), 76.8 (CH), 75.9 (CH_2), 75.8 (CH_2), 75.3 (CH), 73.1 (CH_2), 72.0 (CH), 71.4 (CH), 71.4 (CH), 69.3 (CH_2), 69.2 (CH_2), 68.6 (CH_2), 68.5 (CH_2), 67.8 (CH), 67.6 (CH_2), 67.6 (CH), 65.7 (CH_2) 65.7 (CH_2), 65.5 (CH_2), 65.5 (CH), 60.5 (CH_3) 51.6 (CH_2), 51.4 (CH_2), 50.0 (CH), 49.7 (CH), 48.5 (CH_2), 47.7 (CH), 44.5 (CH), 43.7 (CH), 30.4 (CH_2), 29.1 (CH_2), 28.7 (CH_2), 24.8 (CH_2), 24.7 (CH_2), 16.6 (CH_3), 16.6 (CH_3), 16.6 (CH_3), 16.5(CH_3);

HRMS m/z (ESI, $(M-2H+Na)^-$) calcd for $C_{61}H_{70}N_2O_{22}NaPS2Br^-$ 1379.2680, found 1379.268. A mixture of this *N*-sulfate and Degussa type 20% $Pd(OH)_2/C$ (468 mg) in methanol (12 mL) and pH 7 phosphate buffer (1.0 mL) was purged with nitrogen for 10 min at room temperature. The reaction flask was then equipped with a hydrogen balloon. After stirring for 3 d, the whole mixture was filtered through a pad of Celite®, washed with methanol, and concentrated *in vacuo*. The residue was purified by Sephadex G-25 column followed by AG 50W-X8 cation exchange column (Na^+ form) using water as eluent to obtain compound **30** (23.8 mg, 41% in four steps). ^{1}H NMR (600 MHz, D_2O) δ 5.37 (d, *J* = 3.4 Hz, 1H, H-1'), 5.14 (d, *J* = 1.8 Hz, 1H, H-1), 4.49 (d, *J* = 2.4 Hz, 1H, H-5), 4.43-4.40 (m, 1H, H-6'a), 4.30-4.26 (m, 1H, H-6'b), 4.24-4.19 (m, 6H, H-2, H-3, OEt), 4.05-4.04 (m, 1H, H-4), 3.95-3.93 (m, 1H, H-5'), 3.78-3.74 (m, 1H, linker CH_2), 3.69-3.63 (m, 2H, H-3', linker CH_2), 3.56 (t, *J* = 9.3 Hz, 1H, H-4'), 3.22 (dd, *J* = 3.4, 10.3 Hz, 1H, H-2'), 3.01-2.99 (m, 2H, linker CH_2), 1.70-1.62 (m, 4H, linker CH_2), 1.49-1.43 (m, 2H, linker CH_2), 1.37-1.35 (m, 6H, OEt); ^{13}C NMR (150 MHz, D_2O) δ 174.8 (C), 98.6 (CH), 97.1 (CH), 75.9 (CH), 75.7 (CH), 70.9 (CH), 70.1 (CH), 70.0 (CH), 69.0 (CH), 68.4 (CH), 68.2 (CH), 68.0 (CH), 67.9 (CH_2), 66.1 (CH_2), 66.0 (CH_2), 65.69 (CH_2), 65.65 (CH_2), 65.61 (CH_2), 65.5 (CH_2), 58.0 (CH), 39.3 (CH_2), 27.7 (CH_2), 26.2 (CH_2), 22.2 (CH_2), 15.3 (CH_3), 15.2 (CH_3); HRMS m/z (ESI, $M-H+2Na^+$) calcd for $C_{21}H_{40}N_2O_{20}Na_2PS_2^+$ 781.1149, found 781.1152.

Compound 31.

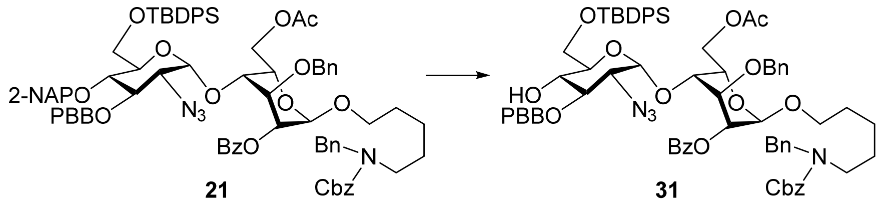

Compound **21** (2.5 g, 1.71 mmol) was dissolved in a 18/1 ratio of dichloromethane/water (95 mL) at room temperature. 2,3-Dichloro-5,6-dicyano-1,4-benzoquinone (DDQ, 1.13 g, 5.13 mmol) was added to the reaction solution in three equal portions in half-hour intervals. After stirring for 4 h, the mixture was filtered through a pad of Celite® followed by washed with dichloromethane. The filtrate was washed with 10% $Na_2S_2O_{4(aq)}$, dried over anhydrous $MgSO_4$, filtered, and concentrated *in vacuo*. The residue was purified by flash column chromatography (EtOAc/Hex = 1/3) on silica gel to provide the 4'-alcohol **31** (1.7 g, 75%). ^{1}H NMR (600 MHz, $CDCl_3$) δ 8.13-8.11 (m, 2H,

Ar-H), 7.66-7.63 (m, 4H, Ar-H), 7.48-7.20 (m, 32H, Ar-H), 7.13-7.11 (m, 3H, Ar-H), 5.17-5.11(m, 3H, H-2, $ArCH_2$), 4.94 (d, J = 10.5 Hz, 1H, H-1), 4.85 (d, J = 11.1 Hz, 1H, $ArCH_2$), 4.70-4.68 (m, 2H, H-1', $ArCH_2$), 4.55 (d, J = 11.0 Hz, 1H, $ArCH_2$), 4.47-4.44 (m, 2H, $ArCH_2$), 4.4-4.37 (m ,1H, H-6a), 4.3-4.27 (m, 2H, $ArCH_2$, H-5), 4.03-4.02 (m, 1H, H-6b), 4.04 (s, 1H, H-3), 3.90-3.88 (m, 1H, H-6'a), 3.80-3.77 (m, 1H, H-6'b), 3.7-3.65 (m, 4H, H-4, H-4', H-5', linker CH_2), 3.60-3.57 (m, 1H, H-3'), 3.42-3.37 (m, 1H, linker CH_2), 3.20-3.13 (m, 3H, H-2', linker CH_2), 2.55 (bs, 1H, OH), 1.97 (bs, 3H, OAc), 1.59-1.47 (m, 4H, linker CH_2), 1.29-1.25 (m, 2H, linker CH_2), 1.04 (s, 9H, TBDPS); ^{13}C NMR (150 MHz, $CDCl_3$) δ 170.4 (C), 165.7 (C), 156.7 (C), 156.1 (C), 137.9 (C), 137.7 (C), 137.0 (C), 135.6 (CH), 135.5 (CH), 133.2 (CH), 132.8 (C), 132.7 (C), 131.5 (CH), 129.97 (CH), 129.95 (CH), 129.8 (CH), 129.6 (CH), 128.55 (CH), 128.50 (CH), 128.3 (CH), 128.0 (CH), 127.89 (CH), 127.84 (CH), 127.2 (CH), 127.1 (CH), 121.7 (C), 98.2 (CH), 98.0 (CH), 80.2 (CH), 74.2 (CH_2), 73.9 (CH), 73.1 (CH), 72.5 (CH_2), 72.0 (CH), 69.4 (CH), 68.0 (CH_2), 67.1 (CH_2), 66.0 (CH), 64.0 (CH_2), 63.0 (CH), 50.5 (CH_2), 50.2 (CH_2), 47.1 (CH_2), 46.2 (CH_2), 29.1 (CH_2), 27.9 (CH_2), 27. 5(CH_2), 26.8 (CH_3), 23.4 (CH_2), 20.8 (CH_3), 19.4 (C); HRMS m/z (ESI, M+Na^+) calcd for $C_{71}H_{79}N_4O_{14}SiBrNa^+$ 1341.4436, found 1341.4443.

Compound 32.

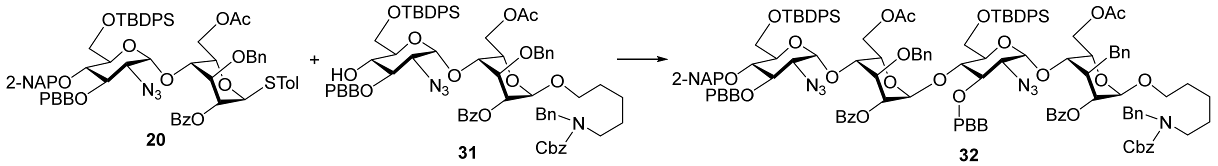

20 31 32

A mixture of the donor **20** (6.8 g, 5.4 mmol), the acceptor **31** (6.8 g, 5.1 mmol), and freshly activated 3Å MS (14.0 g) in dichloromethane (140 mL) was stirred at room temperature for 30 min under nitrogen atmosphere. The reaction flask was cooled down to –78 °C, and *N*-iodosuccinimide (1.4 g, 6.2 mmol) was added to the solution. After stirring for 30 min, trifluoromethanesulfonic acid (0.18 mL, 2.0 mmol) was added to the reaction mixture, and the resulting solution was gradually warmed up to –40 °C for 3 h. The reaction was quenched by 10% $Na_2S_2O_{3(aq)}$ and sat. $NaHCO_{3(aq)}$. The crude reaction mixture was filtered through a pad of Celite® followed by washed with dichloromethane. After separation, the organic layer was washed with water, dried over anhydrous $MgSO_4$, filtered, concentrated *in vacuo*. The crude product was

purified by flash column chromatography (EtOAc/Hex = 1/9 → 1/4) on silica gel to yield compound **32** (10.2 g, 81 %). ^{1}H NMR (600 MHz, $CDCl_3$) δ 8.24 (d, *J* = 7.2 Hz, 2H, Ar-H), 8.13 (d, *J* = 7.0 Hz, 2H, Ar-H), 7.93-7.80 (m, 12H, Ar-H), 7.57-7.41 (m, 45H,Ar-H), 7.12 (d, *J* = 8.4 Hz, 2H, Ar-H), 7.05 (d, *J* = 8.3 Hz, 2H, Ar-H), 5.54 (s, 1H, H-1''), 5.35-5.29 (m, 4H, H-2, H-2'', $ArCH_2$), 5.13-5.04 (m, 5H, H-1, $ArCH_2$), 4.87-4.77 (m, 5H, H-1', H-1''', $ArCH_2$), 4.71 (bs, 1H, H-1'''), 4.61-4.52 (m, 6H, H-6''a, $ArCH_2$), 4.26-4.12 (m, 12H, H-3, H-3'', H-5, H-5'', H-5''', H-6, H-6', H-6''b, H-6'''), 3.92-3.78 (m, 8H, H-3', H-3''', H-4, H-4', H-4'', H-4''', H-5', linker CH_2), 3.59-3.52 (m, 1H, $ArCH_2$), 3.42 (dd, *J* = 10.3, 2.8 Hz, 1H, H-2''), 3.38-3.34 (m, 2H, H-2', linker CH_2), 3.30-3.25 (m, 1H, linker CH_2), 1.94 (d, *J* = 9.3 Hz, 3H, OAc), 1.85 (s, 3H, OAc), 1.78-1.67 (m, 4H, linker CH_2), 1.48-1.41 (m, 2H, linker CH_2), 1.20 (s, 9H, TBDPS), 1.11 (s, 9H, TBDPS) ; ^{13}C NMR (150 MHz, $CDCl_3$) δ 171.0 (C), 170.3 (C), 170.0 (C), 165.8 (C), 165.7 (C), 156.8 (C), 156.2 (C), 138.1 (C), 137.9 (C), 137.6 (C), 137.2 (C), 137.0 (C), 136.9 (C), 136.0 (CH), 136.0 (CH), 135.7 (CH), 135.6 (CH), 133.5 (C), 133.4 (C), 133.3 (CH), 133.15 (C), 133.11 (C), 131.6 (CH), 131.4 (CH), 131.4 (CH), 131.1 (CH), 130.2 (CH), 130.0 (CH), 130.0 (CH), 129.9 (CH), 129.9 (CH), 129.9 (CH), 129.8 (CH), 129.8 (CH), 129.6 (CH), 129.1 (CH), 128.9 (CH), 128.7 (CH), 128.6 (CH), 128.6 (CH), 128.5 (CH), 128.4 (CH), 128.4 (CH), 128.3 (CH), 128.2 (CH), 128.0 (CH), 128.0 (CH), 127.9 (CH), 127.9 (CH), 127.8 (CH), 127.7(CH), 127.6 (CH), 127.4 (CH), 127.3 (CH), 127.3 (CH), 126.6 (CH), 126.3 (CH), 126.2 (CH), 125.9 (CH), 121.8 (C), 121.1 (C), 98.6 (CH), 98.4 (CH), 97.6 (CH), 97.1 (CH), 80.5 (CH), 79.3 (CH), 77.9 (CH), 77.6 (CH), 77.4 (CH), 77.2 (CH), 75.3 (CH_2), 74.3 (CH_2), 74.1 (CH_2), 74.0 (CH), 73.8 (CH), 73.7 (CH), 73.2 (CH), 73.0 (CH_2), 72.9 (CH_2), 72.7 (CH), 72.6 (CH_2), 69.2 (CH), 68.6 (CH), 68.1 (CH_2), 67.2 (CH_2), 65.4 (CH), 64.8 (CH), 64.4 (CH), 64.2 (CH), 62.99 (CH_2), 62.94 (CH_2), 62.5 (CH_2), 62.2 (CH_2), 60.4 (CH_2), 50.7 (CH_2), 50.3 (CH_2), 47.3 (CH_2), 46.3 (CH_2), 29.2 (CH_2), 28.1 (CH_2), 27.6 (CH_2), 27.1 (CH_3), 26.9 (CH_3), 21.1 (CH_3), 20.8 (CH_3), 20.7 (CH_3), 19.6 (C), 19.5 (C), 14.4 (CH_3); HRMS m/z (ESI, $M+2Na^{2+}$) calcd for $C_{133}H_{141}N_7O_{25}Si_2Br_2Na_2^{2+}$ 1247.8839, found 1248.8829.

Compound 33.

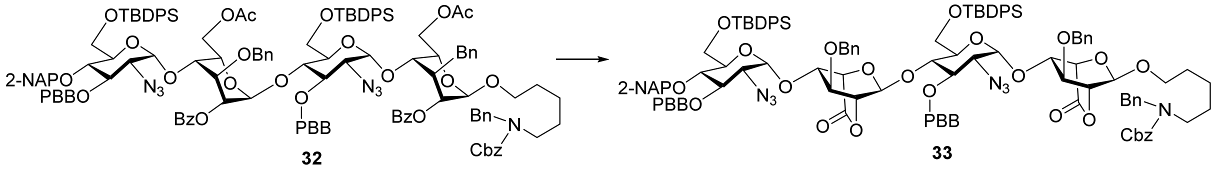

To a solution of compound **32** (4.4 g, 3.0 mmol) in a 1/1 ratio of methanol/dichloromethane (20 mL) was added sodium methoxide (1.9 g, 36 mmol) at 0 °C under nitrogen atmosphere. The mixture was gradually warmed up to room temperature, and the reaction was kept stirring for 18 h. The reaction mixture was neutralized with DOWEX 50 (H^+ exchange resin), filtered, and the solvent was removed under reduced pressure. The crude compound was dissolved in ethyl acetate (100 mL) followed by washed with brine, dried over anhydrous $MgSO_4$, filtered, and concentrated *in vacuo*. The crude residue was dissolved in a 2/1 ratio of dichloromethane/water (35 mL) at room temperature. (2,2,6,6-Tetramethylpiperidin-1-yl)oxyl (TEMPO, 86 mg, 0.55 mmol) and bis(acetoxy)iodobenzene (BAIB, 2.2 g, 6.8 mmol) were consecutively added to the solution, and the reaction mixture was kept stirring for 16 h. The reaction was quenched with 10 % $Na_2S_2O_{3(aq)}$, and the organic layer was washed with water, dried over anhydrous $MgSO_4$, filtered, and concentrated *in vacuo*. The residue was purified by flash column chromatography (EtOAc/Hex = 1/20 → 1/5) on silica gel to provide the lactone **33** (2.7 g, 70% in two steps). ^1H NMR (600 MHz, $CDCl_3$) δ 7.87-7.85 (m, 1H, Ar-H), 7.80-7.76 (m, 4H, Ar-H), 7.71-7.64 (m, 7H, Ar-H), 7.52-7.50 (m, 4H, Ar-H), 7.46-7.43 (m, 4H, Ar-H), 7.4-7.30 (m, 32H, Ar-H), 7.24-7.21 (m, 6H, Ar-H), 5.47 (bs, 1H, H-1''), 5.23-4.93 (m, 7H, H-1, H-1', $ArCH_2$), 4.87-4.51(m, 10H, H-2, H-2'', $ArCH_2$), 4.35-4.22 (m, 5H, H-1''', H-3'', H-5, H-5'', $ArCH_2$), 4.10 (t, 1H, H-4''), 4.05-4.03 (m, 1H, H-6'a), 3.96-3.77 (m, 11H,H-3, H-3', H-3''', H-4, H-4''', H-5', H-5''', H-6'b, H-6''', linker CH_2), 3.70-3.68 (m, 1H, H-4'), 3.51-3.44 (m, 1H, linker CH_2), 3.40-3.38 (m, 1H, H-2'), 3.35 (dd, J = 10.2, 3.7 Hz, 1H, H-2'''), 3.31-3.22 (m, 2H, linker CH_2), 1.68-1.54 (m, 4H, linker CH_2), 1.39-1.30 (m, 2H, linker CH_2), 1.13 (s, 9H, TBDPS), 1.08 (s, 9H, TBDPS); ^{13}C NMR (150 MHz, $CDCl_3$) δ 167.8 (C), 167.1 (C), 156.8 (C), 156.3 (C), 138.0 (C), 137.6 (C), 137.3 (C), 137.0 (C) 136.9 (C), 136.8 (C), 136.1 (C), 136.0 (CH), 135.9 (CH), 135.69 (CH), 135.6 (CH), 135.59 (C), 133.7 (C), 133.4 (C), 133.0 (C), 133.0 (C), 132.9 (C), 132.8 (C), 131.7 (CH), 131.6 (CH), 130.1 (CH), 129.95 (CH), 129.93 (CH), 129.9 (CH), 129.87 (CH), 129.83 (CH), 129.8 (CH), 128.69 (CH), 128.65 (CH), 128.62 (CH), 128.57 (CH), 128.3 (CH), 128.1 (CH), 128.08 (CH), 128.06 (CH), 127.9 (CH), 127.89 (CH), 127.86 (CH), 127.82 (CH), 127.7 (CH), 127.6 (CH), 127.2 (CH), 126.27 (CH), 126.25 (CH), 126.06 (CH), 125.6 (CH), 121.9 (C), 121.3 (C), 99.9 (CH), 99.6 (CH), 97.2 (CH), 97.0 (CH), 80.7 (CH), 80.4 (CH), 79.9 (CH), 79.4 (CH), 78.5 (CH), 78.1 (CH), 77.8 (CH), 76.5 (CH), 75.2 (CH_2), 74.8 (CH_2), 73.6 (CH_2), 72.8 (CH), 72.4 (CH), 72.3 (CH), 72.24 (CH), 72.2 (CH_2), 69.7 (CH_2), 69.5 (CH), 69.2 (CH), 67.3 (CH_2), 63.5 (CH), 63.2 (CH), 61.9 (CH_2), 61.8 (CH_2), 50.6 (CH_2), 50.3

(CH2), 47.2 (CH2), 46.1 (CH2), 29.2 (CH2), 27.9 (CH2), 27.4 (CH2) 27.06 (CH3), 27.02 (CH3), 23.2 (CH2), 19.4 (C), 19.3 (C); HRMS m/z (ESI, M+Na$^+$) calcd for $C_{115}H_{121}N_7O_{21}Si_2Br_2Na^+$ 2175.6434, found 2175.6418.

Compound 34.

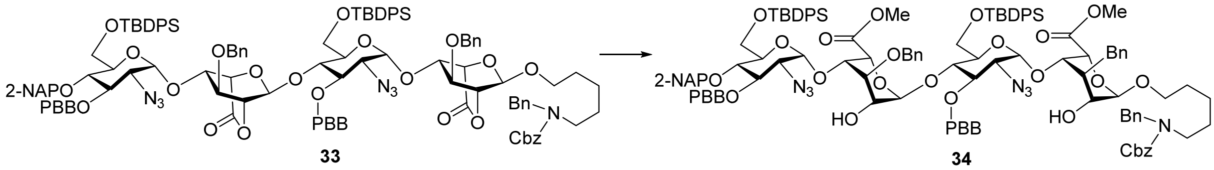

Triethylamine (78 μL, 0.028 mmol) was added to a solution of compound **33** (2.0 g, 0.92 mmol) in 1/2 ratio of methanol/dichloromethane (60 mL) at room temperature. The mixture was warmed up and kept stirring at 40 °C for 18 h. The reaction mixture was cooled down to room temperature and concentrated at the reduced pressure. The crude residue was purified by flash column chromatography (EtOAc/Hex = 1/9 → 1/1) on silica gel to afford the diol **34** (2.0 g, 97 %). ^{1}H NMR (600 MHz, CDCl3) δ 7.86-7.75 (m, 10H, Ar-H), 7.70 (d, *J* = 7.2 Hz, 2H, Ar-H), 7.64 (s, 1H, Ar-H), 7.53-7.51 (m, 2H, Ar-H), 7.48-7.27 (m, 44H, Ar-H), 7.24-7.23 (m, 1H, Ar-H), 7.20-7.17 (m, 2H, Ar-H), 7.09-7.08 (m, 2H, Ar-H), 5.42 (s, 1H, H-1''), 5.21 (d, *J* = 15.9 Hz, 2H, ArCH2), 5.08-5.07(m, 2H, H-1, H-1') 5.01-4.93 (m, 3H, H-1''', ArCH2), 4.87-4.49 (m, 13H, H-2, H-2'', H-3'', H-5, H-5'', ArCH2), 4.14-4.10 (m, 2H, H-3, H-4'), 4.04-3.83 (m, 10H, H-4, H-5''', H-6', H-6''', linker CH2, ArCH2), 3.78-3.67 (m, 3H, H-3''', H-4''', H-5'), 3.61-3.54 (m, 9H, H-2', H-2''', H-3', linker CH2, CO2Me), 3.45-3.43 (m, 1H, H-4'), 3.33 (s, 3H, CO2Me), 3.29-3.19 (m, 2H, linker CH2), 1.65-1.51 (m, 4H, linker CH2), 1.36-1.29 (m, 2H, linker CH2), 1.15-1.14 (m 18H, TBDPS); ^{13}C NMR (150 MHz, CDCl3) δ 169.9 (C), 169.0 (C), 156.7 (C), 156.2 (C), 138 (C), 137.6 (C), 137.2 (C), 136.8 (C), 136.7 (C), 136.05 (CH), 136.00 (CH), 135.9 (CH), 135.67 (C), 135.64 (CH), 133.4 (C), 133.39 (C), 133.31 (C), 133.1 (C), 133.0 (C), 131.6 (CH), 131.2 (CH), 129.9 (CH), 129.89 (CH), 129.86 (CH), 129.7 (CH), 129.1 (CH), 128.8 (CH), 128.6 (CH), 128.5 (CH), 128.4 (CH), 128.3 (CH), 128.2 (CH), 128.0 (CH), 127.99 (CH), 127.94 (CH), 127.86 (CH), 127.81 (CH), 127.68 (CH), 127.6 (CH), 127.4 (CH), 127.3 (CH), 127.2 (CH), 126.3 (CH), 126.0 (CH), 125.9 (CH),125.3 (CH), 121.9 (C), 121.2 (C), 101.6 (CH), 100.9 (CH), 95.4 (CH), 94.9 (CH), 80.9 (CH), 79.6 (CH), 77.7 (CH), 75.0 (CH2), 74.2 (CH2), 74.0 (CH2), 72.9 (CH), 72.7 (CH2), 72.6 (CH), 71.8

(CH), 71.7 (CH), 71.6 (CH), 68.6 (CH_2), 68.5 (CH_2), 67.4 (CH), 67.2 (CH_2), 66.9 (CH), 66.8 (CH), 66.2 (CH), 66.1 (CH), 64.2 (CH), 63.8 (CH), 62.3 (CH_2), 62.0 (CH_2), 52.1 (CH), 51.8 (CH), 50.5 (CH_2), 50.2 (CH_2), 47.1 (CH_2), 46.1 (CH_2), 29.2 (CH_2), 28.0 (CH_2), 27.5 (CH_2), 27.06 (CH_3), 27.04 (CH_3), 23.5 (CH_2), 23.4 (CH_2), 19.52 (CH_3), 19.50 (CH_3). HRMS m/z (ESI, M+Na^+) calcd for $C_{117}H_{129}N_7O_{23}Si_2Br_2Na^+$ 2236.6943, found 2236.6885.

Compound 35.

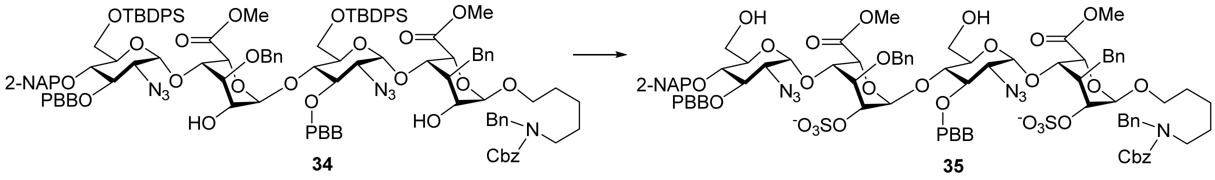

Compound **34** (200 mg, 91.6 μmol) was dissolved in *N*,*N*-dimethylformamide (2.0 mL) at room temperature, sulfurtrioxide•triethylamine (199 mg, 1.09 mmol) was added to the solution, and the reaction mixture was warmed up and kept stirring at 60 °C for 18 h. The reaction flask was cooled down to room temperature, water (2 mL) and sodium bicarbonate solid (400 mg) were added to the solution, and the resulting mixture was continuously stirred for 3 h. The solvent was removed at the reduced pressure, and the crude residue was dissolved in dichloromethane (20 mL) followed by washed with sat. $NaHCO_{3(aq)}$ and water. The organic layer was dried over anhydrous $MgSO_4$, filtered, and concentrated *in vacuo*. The crude residue was purified by flash column chromatography (methanol/chloroform = 1/20 → 1/15 → 1/10 → 1/5) on silica gel to furnish the *O*-sulfonated product (128 mg, 75%). This sulfate compound (0.10 g, 42 μmol) was dissolved in tetrahydrofuran (0.8 mL) and pyridine (0.8 mL) at room temperature. The reaction flask was cooled down to 0 °C, a ~70%/~30% ratio of hydrogen fluoride/pyridine (0.6 mL) was slowly added to the solution, and the mixture was kept stirring for 3 d. The reaction was quenched by silica gel (1 g), and the mixture was continuously stirred for 1 h. The whole mixture was filtered, and the solid part was washed with 1/1 ratio of methanol/chloroform (5 mL). The filtrate was concentrated *in vacuo*, and the crude residue was purified by flash column chromatography (methanol/chloroform = 1/20 → 1/15 → 1/10 → 1/4) on silica gel to give the diol **35** (60 mg, 76%). ^1H NMR (600 MHz, CD_3OD) δ 7.73-7.70 (m, 3H, Ar-H), 7.59 (s, 1H, Ar-H), 7.41-7.37 (m, 6H, Ar-H), 7.29-7.26 (m, 10H, Ar-H), 7.22-7.17 (m, 11H, Ar-H), 7.10-7.08 (m, 2H, Ar-H), 6.95 (d, *J*

= 8.4 Hz, 2H, Ar-H), 5.31 (s, 1H, H-1''), 5.10-5.03 (m, 5H, H-1, H-1', H-1''', ArCH2), 4.76-4.71 (m, 7H, H-5, H-5'', ArCH2), 4.59-4.50(m, 7H, H-2, H-2'', ArCH2), 4.38-4.36 (m ,2H, ArCH2), 4.24-4.22 (m, 2H, H-3, H-3''), 4.07(s, 1H, H-4''), 3.92-3.87 (m, 4H, H-3''', H-4, H-6'''), 3.75-3.73 (m, 1H, H-6'a), 3.70-3.67 (m, 6H, H-3', H-5''', H-6'b, CO2Me), 3.60-3.57 (m 4H, H-4', H-4''', H-5', linker CH2), 3.42-3.37 (m, 4H, linker CH2, CO2Me), 3.22-3.19 (m, 2H, H-2', H-2'''), 3.14-3.09 (m, 2H, linker CH2), 1.53-1.44 (m, 4H, linker CH2), 1.26-1.20 (m, 2H, linker CH2); ^{13}C NMR (150 MHz, CD3OD) δ 170.1 (C), 170.0 (C), 157.1 (C), 156.5 (C), 138.0 (C), 137.77 (C), 137.75 (C), 137.4 (C), 135.9 (C), 133.3 (C), 133.0 (C), 130.9 (CH), 130.7 (CH), 129.4 (CH), 129.1 (CH), 128.4 (CH), 128.3 (CH), 128.2 (CH), 128.1 (CH), 127.9 (CH), 127.8 (CH), 127.6 (CH), 127.5 (CH), 127.36 (CH), 127.31 (CH), 126.9 (CH), 125.8 (CH), 125.7 (CH), 125.5 (CH), 125.4 (CH), 120.8 (C), 120.5 (C), 99.4 (CH), 98.3 (CH), 97.1 (CH), 95.7(CH), 79.7 (CH), 78.2 (CH), 77.6 (CH), 74.2 (CH2), 73.8 (CH2), 73.2 (CH2), 73.0 (CH), 72.5 (CH), 72.4 (CH), 72.0 (CH2), 71.6 (CH2), 70.9 (CH), 70.5 (CH), 67.9 (CH2), 67.3 (CH2), 67.2 (CH), 67.1 (CH2), 66.9 (CH2), 63.8 (CH), 63.5 (CH), 60.0 (CH2), 59. 8(CH2), 51.6 (CH3), 51.2 (CH3), 50.1 (CH2), 49.9 (CH2), 48.4 (CH), 48.2 (CH), 47.1 (CH), 47.0 (CH), 46.1 (CH2), 28.8 (CH2), 27.5 (CH2), 27.1 (CH2), 23.2 (CH2), 23.1 (CH2); HRMS m/z (ESI, M-2H^{2-}) calcd for $C_{85}H_{91}N_7O_{29}S_2Br_2^{2-}$ 948.1851, found 948.1830.

Compound 36.

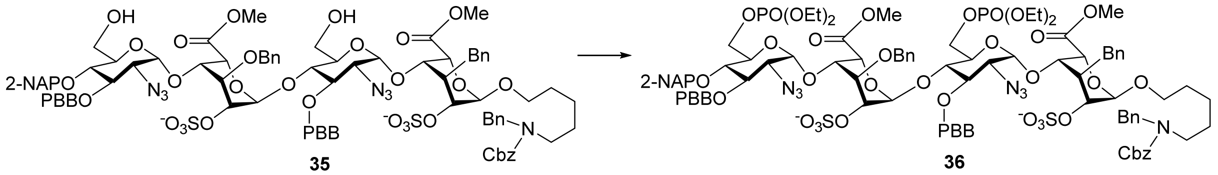

To a solution of compound **35** (40 mg, 21 μmol) in pyridine (1 mL) was added diethylphosphoryl chloride (40 μL, 0.26 mmol) at room temperature under nitrogen atmosphere. After stirring for 2 h, the reaction was quenched by methanol (0.1 mL) and the resulting mixture was continuously stirred for another 30 min. The solvent was removed at the reduced pressure, and the crude residue was purified by flash column chromatography (methanol/chloroform = 1/20 → 1/15 → 1/10 → 1/7 → 1/4) on silica gel to provide the compound **36** (41 mg, 90%). ^{1}H NMR (600 MHz, CD3OD) δ 7.81-7.76 (m, 3H, Ar-H), 7.65 (s, 1H, Ar-H), 7.49-7.41 (m,7H, Ar-H), 7.38-7.35 (m, 8H, Ar-H), 7.32-7.09 (m, 14H, Ar-H), 7.02-7.01 (d, *J* = 8.3 Hz, 2H, Ar-H), 5.29 (bs, 1H, H-1''), 5.15-5.10 (m,

4H, H-1, H-1"‘’, ArCH2), 4.95 (d, J = 2.5 Hz, 1H, H-1'), 4.82-4.81 (m, 2H, ArCH2), 4.80-4.77 (m, 5H, ArCH2), 4.73 (s, 1H, H-5"), 4.64-4.56 (m, 5H, H-2", H-5, ArCH2), 4.51 (bs, 1H, H-2), 4.45-4.42 (m, 3H, H-6'", ArCH2), 4.38-4.35 (m, 1H, ArCH2), 4.31 (bs, 1H,H-3), 4.21-4.13 (m, 7H, H-3", H-4, H-6'a, OEt), 4.07-3.97 (m, 4H, OEt), 3.93-3.92 (m, 1H, H-3'), 3.88-3.86 (m, 3H, H-4", H-5'", H-6'b), 3.77-3.71 (m, 6H, H-3'", H-4'", H-5', CO2Me), 3.61-3.54 (m, 2H, H-4’, linker CH2), 3.39-3.35 (m, 4H, linker CH2, CO2Me), 3.27-3.26 (m, 1H, H-2'"), 3.28-3.27 (m, 1H, H-2'), 3.18-3.13 (m, 2H, linker CH2), 1.51-1.44 (m, 4H, linker CH2), 1.29-1.23 (m, 14H, OEt, linker CH2); ^{13}C NMR (150 MHz, CD3OD) δ 170.2 (C), 169.5 (C), 157.0 (C), 156.5 (C), 148.6 (C), 138.0 (C), 137.8 (C), 137.7 (C), 137.5 (C), 137.3 (C), 137.1 (C), 136.7 (C), 135.5 (C), 133.3 (C), 133.0 (C), 131.0 (C), 130.9 (CH), 129.4 (CH), 129.0 (CH), 128.5 (CH), 128.3 (CH), 128.2 (CH), 128.1 (CH), 127.9 (CH), 127.7 (CH), 127.59 (CH), 127.57 (CH), 127.4 (CH), 127.3 (CH), 126.9 (CH), 126.1 (CH), 125.8 (CH), 125.7 (CH), 125.6 (CH), 120.9 (C), 120.5 (C), 99.6 (CH), 98.1 (CH), 96.6 (CH), 80.0 (CH), 78.2 (CH), 77.4 (CH), 74.6 (CH2), 74.0 (CH2), 73.6 (CH2), 73.4 (CH), 72.5 (CH), 72.2 (CH), 72.1 (CH), 72.0 (CH2), 71.5 (CH2), 70.7 (CH), 70.6 (CH), 70.5 (CH), 69.8 (CH), 67.99 (CH2), 67.92 (CH2), 67.1 (CH), 66.9 (CH2), 66.8 (CH), 66.6 (CH), 66.1 (CH2), 65.77 (CH2), 65.74 (CH2), 65.3 (CH2), 65.16 (CH2), 65.12 (CH2), 64.25 (CH2), 64.21 (CH2), 64.17 (CH2), 64.13 (CH2), 63.9 (CH), 63.7 (CH), 51.6 (CH3), 51.2 (CH3), 50.1 (CH2), 49.9 (CH2), 46.1 (CH2), 28.8 (CH2), 27.5 (CH2), 27.1 (CH2), 23.2 (CH2), 23.1 (CH2), 15.3 (C), 15.2 (C), 15.17 (C), 15.15 (C), 15.12 (C), 15.0 (C); HRMS m/z (ESI, M-2H^{2-}) calcd for $C_{93}H_{109}Br_2N_7O_{35}P_2S_2^{2-}$ 1085.2130, found 1085.2118.

Compound 37.

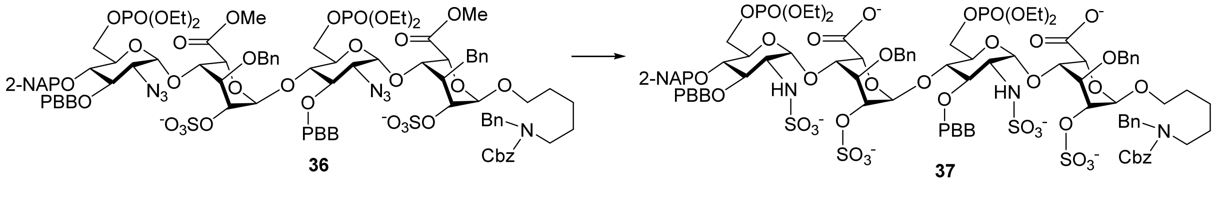

Compound **36** (93 mg, 42.8 μmol) was dissolved in tetrahydrofuran (6.0 mL) at room temperature. A 1.0 N solution lithium hydroxide in water (4 mL) and a 37% solution of hydrogen peroxide in water (0.85 mL) were consecutively added to the reaction solution. The mixture was warmed up and stirred at 37 °C for 18 h. After cooling to room temperature, the reaction was neutralized by

acetic acid till pH 7, and the resulting mixture was concentrated *in vacuo*. The crude compound was dissolved in tetrahydrofuran (3 mL) at room temperature, a 0.1 N sodium hydroxide aqueous solution (0.3 mL) and a 1 M solution of trimethylphosphine in tetrahydrofuran (294 μL, 294 μmol) were sequentially added into the reaction flask, and the mixture was continuously stirred for 14 h. The reaction was quenched by 0.1 N $HCl_{(aq)}$ (0.3 mL), the mixture was concentrated *in vacuo*, and the residue was purified by flash column chromatography ($MeOH/CHCl_3$ = 1/9 → 1/4) on silica gel followed by Na^+ ion exchange column (AG 50W-X8 cation exchange resin) using methanol as eluent to obtain the crude diamine product. This diamine compound was dissolved in methanol (3 mL) at room temperature. A solution of 0.1 N sodium hydroxide aqueous solution (1.5 mL, 0.15 mmol), triethylamine (1.5 mL, 11 mmol) and sulfur trioxide/pyridine complex (118 mg, 0.74 mmol) were consecutively added to the solution, and the reaction flask was kept stirring for 18 h. The mixture was concentrated under the reduced pressure, and the crude residue was purified by flash column chromatography ($MeOH/CHCl_3$ = 1/14 → 1/10 → 1/9 → 1/7 → 1/5 → 1/3) on silica gel to provide the *N*-sulfate **37** (35 mg, 36% in 4 steps). ^1H NMR (600 MHz, CD_3OD) δ 7.73-7.68 (m, 3H, Ar-H), 7.56 (bs, 1H, Ar-H), 7.38-7.35 (m, 2H, Ar-H), 7.30-7.25 (m, 9H, Ar-H), 7.19-7.04 (m, 20H, Ar-H), 5.29-5.01 (m, 8H, H-1, H-1', H-1'', H-1'‘’,$ArCH_2$), 4.85-4.82 (m, 2H, $ArCH_2$), 4.64-4.54 (5H, H-5, H-5'', $ArCH_2$), 4.41-3.81 (m, 27H, H-2, H-2'', H-3, H-3', H-3'', H-3''', H-4, H-4'', H-5', H-5'‘’, H-6', H-6''', OEt, $ArCH_2$), 3.57-3.33 (m, 6H, H-2', H-2''', H-4', H-4''', linker CH_2), 3.02 (bs, 2H, linker CH_2), 1.48-1.35 (m, 4H, linker CH_2), 1.22-1.10 (m, 14H, OEt, linker CH_2); ^{13}C NMR (150 MHz, CD_3OD) δ 175.6 (C), 173.5 (C), 157.0 (C), 156.5 (C), 138.8 (C), 138.2 (C), 138.1 (C), 137.9 (C), 137.8 (C), 136.7 (C), 135.8 (C), 133.3 (C), 133.0 (C), 130.8 (CH), 130.5 (CH), 130.1 (CH), 129.9 (CH), 128.2 (CH), 128.1 (CH), 127.7 (CH), 127.6 (CH), 127.5 (CH), 127.3 (CH), 127.1 (CH), 127.09 (CH), 127.03 (CH), 126.9 (CH), 126.2 (CH), 125.8 (CH), 125.7 (CH), 125.5 (CH), 120.6(C), 120.2 (C), 98.9 (CH), 98.8 (CH), 98.6 (CH), 80.2 (CH), 77.1 (CH), 75.0 (CH), 74.7 (CH_2), 74.1 (CH_2), 73.7 (CH), 73.3 (CH), 71.3 (CH_2), 70.3 (CH), 70.1 (CH), 69.6 (CH), 69.5 (CH), 67.9 (CH_2), 67.7 (CH_2), 67.0 (CH_2), 66.9 (CH_2), 66.2 (CH_2), 66.1 (CH_2), 64.17 (CH_2), 64.13 (CH_2), 64.08 (CH_2), 64.02 (CH_2), 63.9 (CH_2), 59.2 (CH), 58.9 (CH), 50.1 (CH_2), 49.8 (CH_2), 48.2 (CH), 46.9 (CH_2), 46.1 (CH_2), 28.8 (CH_2), 27.6 (CH_2), 27.0 (CH_2), 23.1 (CH_2), 15.1 (CH_3), 15.0 (CH_3); HRMS m/z (ESI, $M-3H+K^{2-}$) calcd for $C_{91}H_{108}Br_2N_3KO_{41}P_2S_4^{2-}$ 1143.6405, found 1143.6426.

Compound 38.

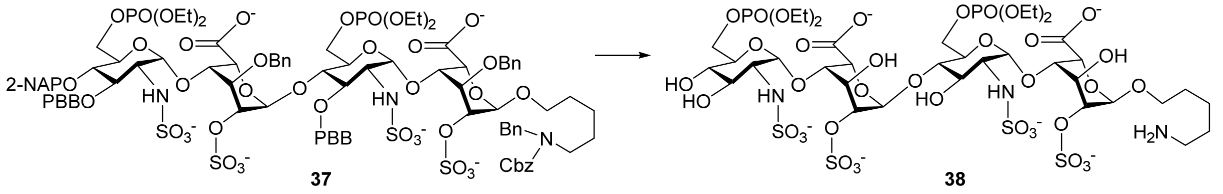

A mixture of compound **37** (24 mg, 10.6 μmol) and Degussa type 20% $Pd(OH)_2/C$ (80 mg) in methanol (2 mL) and pH 7 phosphate buffer (1.0 mL) was purged with nitrogen for 10 min at room temperature. The reaction flask was then equipped with a hydrogen balloon. After stirring for 3 d, the whole mixture was filtered through a pad of Celite®, washed with methanol, and concentrated *in vacuo*. The residue was purified by Sephadex G-25 column followed by AG 50W-X8 cation exchange column (Na^+ form) using water as eluent to yield the product **38** (9.0 mg, 62%). ^{1}H NMR (600 MHz, D_2O) δ 5.40-5.33 (m, 2H, H-1', H-1'''), 5.24 (bs, 1H, H-1''), 5.14 (s, 1H, H-1),5.02 (d, *J* = 2.0 Hz, 1H, H-5), 4.65-4.58 (m, 1H, H-5''), 4.46-4.34 (m, 4H, H-6', H-6'''), 4.30-4.20 (m, 12H, H-2, H-2'', H-3'', H-5', OEt), 4.09 (bs, 2H, H-4, H-4''), 3.97-3.95 (m, 2H, H-3, H-3') 3.79-3.75(m, 2H, H-3''', H-4''') 3.72-3.65 (m, 3H, H-5''', linker CH_2), 3.58-.355 (m, 1H, H-4') 3.30-3.25 (m, 2H, H-2', H-2'''), 3.01 (t, *J* = 7.5 Hz, 1H, linkerCH_2), 1.75-1.60 (m, 4H, linker CH_2), 1.49-1.35 (m, 14H, OEt, linker CH_2); ^{13}C NMR (150 MHz, D_2O) δ 99.1 (CH), 97.4 (CH), 96.5 (CH), 77.0 (CH),76.2 (CH), 75.9 (CH), 75.9 (CH), 75.1 (CH), 70.9 (CH), 70.2 (CH), 70.1 (CH), 69.5 (CH), 69.4 (CH), 69.1 (CH), 68.5 (CH), 68.3 (CH), 68.0 (CH_2), 66.3 (CH_2), 66.1 (CH_2), 65.7 (CH_2), 65.7 (CH_2), 65.6 (CH_2), 65.6 (CH_2), 65.6 (CH_2), 65.5 (CH_2), 58.1, 39.4 (CH_2), 27.8 (CH_2), 26.1 (CH_2), 22.1 (CH_2), 15.4 (CH_3), 15.39 (CH_3), 15.35 (CH_2), 15.31 (CH_3); ^{31}P NMR (202 MHz, D_2O) δ 0.0023; HRMS m/z (ESI, M-3H+Na^{2-}) calcd for $C_{37}H_{66}N_3NaO_{39}P_2S_4^{2-}$ 694.5765, found 694.5756.

3. Biological Procedures

Heparin sodium was manufactured from porcine intestinal mucosa. Its strength, quality and purity met the specifications (Anti-Factor IIa activity: 204 USP units/mg, Anti-Factor Xa activity: 195 units/mg, Anti-clotting activity: 197 IU/mg, Bacterial endotoxins: < 0.0015 EU/u.)

Synthetic human Aβ1–42 was purchased from the Peptide Institute (cat #: 4349-v; Osaka, Japan), and heparin (average molecular weight 13,000, and <38% sulfur content, lot #: PH-72210) was

purchased from Celsus Laboratories (Cincinnati, OH). Thioflavin T (ThT) was form Sigma (cat #: T3516; St. Louis, MO).

Measuring Aβ Fibril Formation

Fibril formation of Aβ was measured by means of a ThT assay. Aβ (10 μM) in phosphate-buffered saline (PBS, pH 7.4) was incubated in the presence of 10 μM ThT and heparin (40 μg/mL) or HS/heparin derivatives (100 μM) at 37 °C with agitation on an orbital rotator at 10 rpm. Fluorescence of ThT was recorded from 460 to 600 nm with an excitation wavelength of 445 nm.

AFM Analysis

Aβ assemblies for morphologic analysis by using an atomic force microscope (AFM) were prepared by incubating Aβ (10 μM) in PBS in the presence of heparin (40 μg/mL) without or with a HS derivative (100 μM) at 37 °C for 24 h. Ten μL of each fibril solution in PBS was diluted with 45 μL of distilled water and spotted on a freshly cleaved mica (Nilaco Corp., Tokyo, Japan), after which the mica was washed with 20 μL of distilled water. Sample images were obtained under ambient conditions at room temperature by using NanoScope IIIa Tapping Mode AFM (Veeco Instrument, Plainview, NY) and a single-crystal microcantilever OMCLAC160TS-R3 (Olympus, Tokyo, Japan) at a scan rate of 0.5 Hz in tapping mode.

Statistical analysis

The data were analyzed using an ordinary two-way analysis of variance (ANOVA) with Dunnett's test or Tukey's range test using Prism software (GraphPad Software, La Jolla, CA, USA). Differences were regarded as significant for $P < 0.05$.

4. References

[1] M. M. L. Zulueta, S.-Y. Lin, Y.-T. Lin, C.-J. Huang, C.-C. Wang, C.-C. Ku, Z. Shi, C.-L. Chyan, D. Irene, L.-H. Lim, T.-I. Tsai, Y.-P. Hu, S. D. Arco, C.-H. Wong, S.-C. Hung, *J. Am. Chem. Soc.* **2012**, *134*, 8988-8995.

[2] Lee, J.-C.; Lu, X.-A.; Kulkarni, S. S.; Wen, Y.-S.; Hung, S.-C. Synthesis of Heparin Oligosaccharides. *J. Am. Chem. Soc.* **2004**, *126*, 476–477.

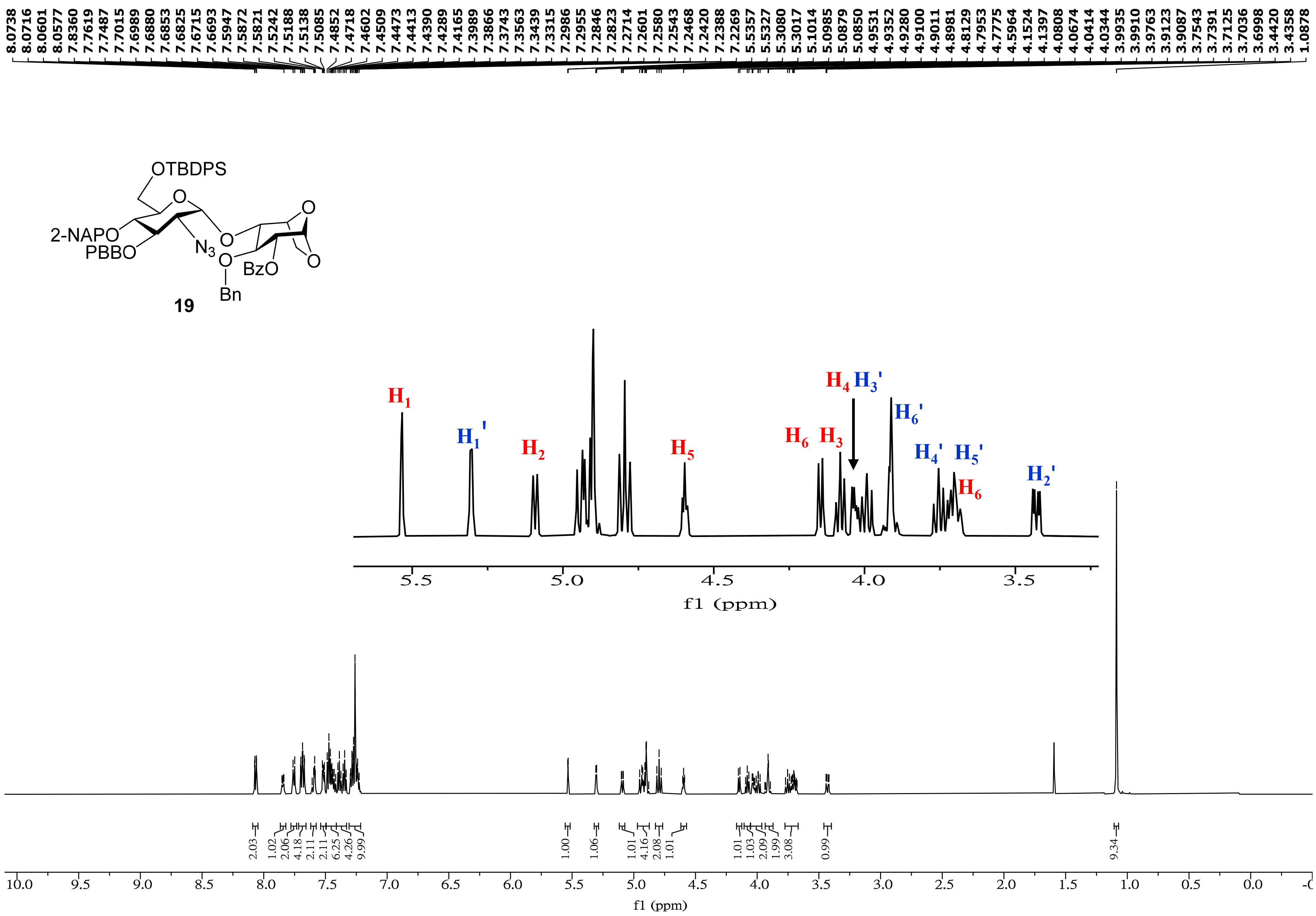
8.0738 8.0716 8.0601 8.0577 7.8360 7.7619 7.7487 7.7015 7.6989 7.6880 7.6853 7.6825 7.6715 7.6693 7.5947 7.5872 7.5821 7.5242 7.5188 7.5138 7.5085 7.4852 7.4718 7.4602 7.4509 7.4473 7.4413 7.4390 7.4289 7.4165 7.3989 7.3866 7.3743 7.3563 7.3439 7.3315 7.2986 7.2955 7.2846 7.2823 7.2714 7.2601 7.2580 7.2543 7.2468 7.2420 7.2388 7.2269 5.5357 5.5327 5.3080 5.3017 5.1014 5.0985 5.0879 5.0850 4.9531 4.9352 4.9280 4.9100 4.9011 4.8981 4.8129 4.7953 4.7775 4.5964 4.1524 4.1397 4.0808 4.0674 4.0414 4.0344 3.9935 3.9910 3.9763 3.9123 3.9087 3.7543 3.7391 3.7125 3.7036 3.6998 3.4420 3.4358 1.0878
OTBDPS
O
2-NAPO
PBBO
N3
O
O
O
BzO
O
O
Bn
H1
H1'
H2
H5
H6
H3
H4
H3'
H6'
H4'
H5'
H6
H2'
f1 (ppm)
2.03 1.02 2.06 4.18 2.11 2.11 6.25 4.26 9.99 1.00 1.06 1.01 4.16 2.08 1.01 1.01 1.03 2.09 1.99 3.08 0.99 9.34
f1 (ppm)

19

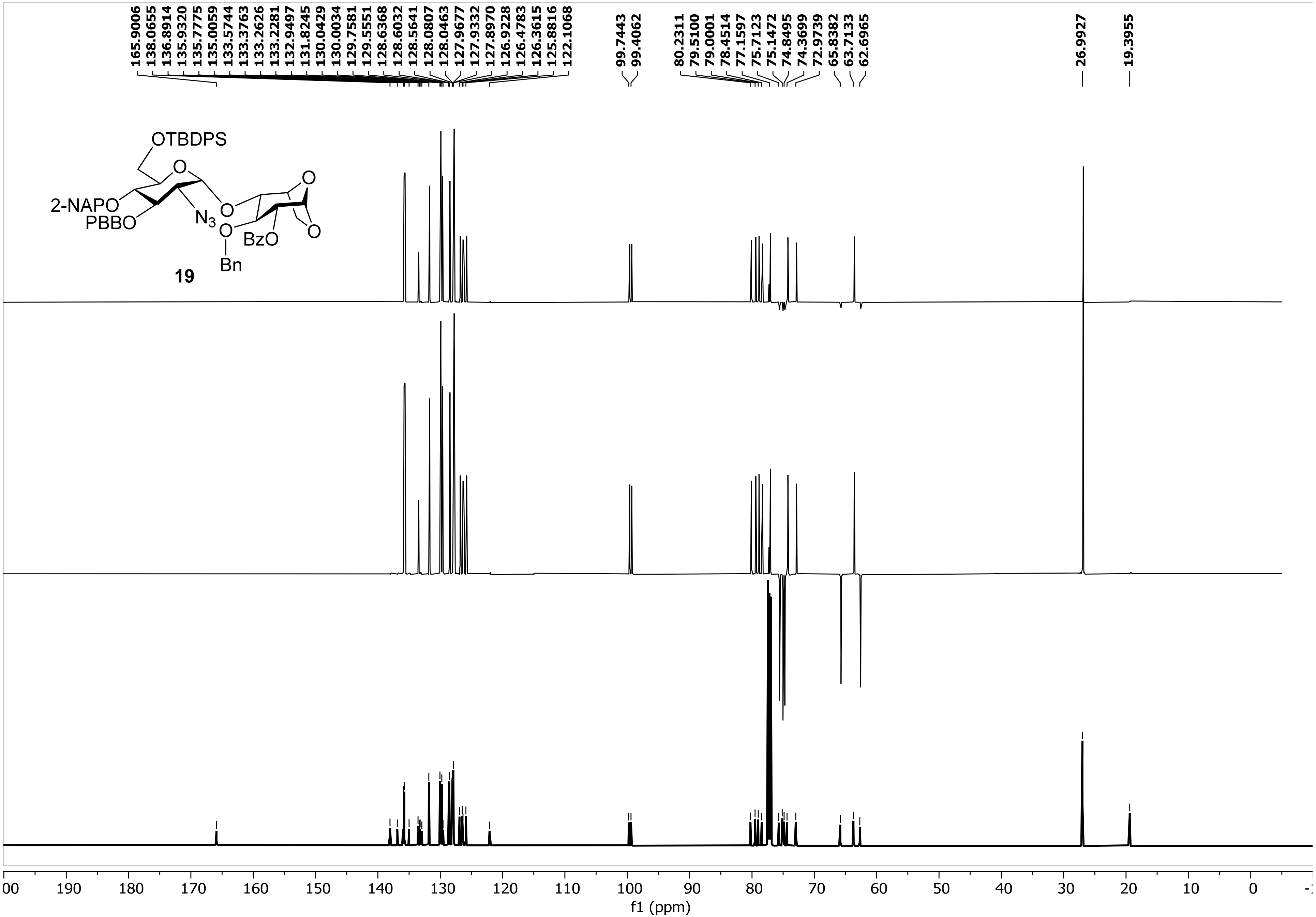

165.9006
138.0655
136.8914
135.9320
135.7775
135.0059
133.5744
133.3763
133.2626
133.2281
132.9497
131.8245
130.0429
130.0034
129.7581
129.5551
128.6368
128.6032
128.5641
128.0807
128.0463
127.9677
127.9332
127.8970
126.9228
126.4783
126.3615
125.8816
122.1068
99.7443
99.4062
80.2311
79.5100
79.0001
78.4514
77.1597
75.7123
75.1472
74.8495
74.3699
72.9739
65.8382
63.7133
62.6965
26.9927
19.3955
OTBDPS
2-NAPO
PBBO
N_3
O
BzO
Bn
19
190
180
170
160
150
140
130
120
110
100
90
80
70
60
50
40
30
20
10
0
f1 (ppm)

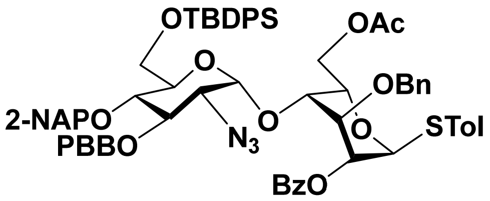
OTBDPS
OAc
OBn
O
2-NAPO
PBBO
N_3
STol
BzO

20

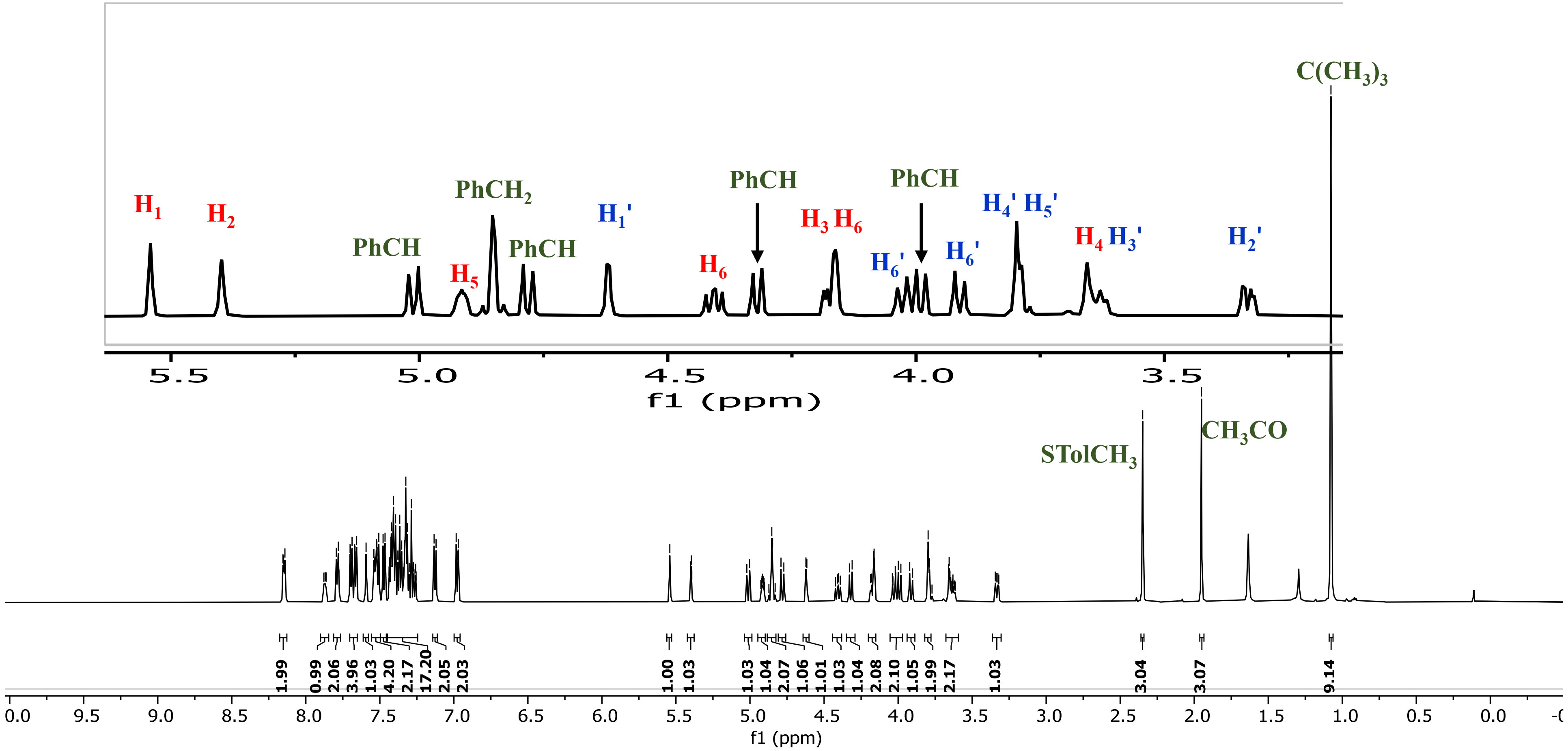
8.1546 8.1498 8.1458 8.1414 8.1381 7.7941 7.7862 7.7804 7.7036 7.7012 7.6988 7.6903 7.6878 7.6854 7.6705 7.6678 7.6596 7.6568 7.6543 7.5934 7.5403 7.5357 7.5325 7.5282 7.5242 7.5203 7.5181 7.5074 7.4822 7.4785 7.4675 7.4647 7.4379 7.4349 7.4258 7.4222 7.4083 7.3941 7.3783 7.3661 7.3556 7.3529 7.3414 7.3338 7.3250 7.3139 7.3017 7.2875 7.2721 7.2691 7.2581 7.1338 7.1207 6.9843 6.9730 6.9701 5.5412 5.4015 5.3971 5.0216 5.0022 4.8530 4.8482 4.7908 4.7715 4.6225 4.6165 4.3284 4.3103 4.1661 4.1617 4.1580 4.0188 3.9991 3.9811 3.9218 3.9024 3.7973 3.7878 3.6594 3.6550 2.3482 1.9510 1.0802 1.0766
$C(CH_3)_3$
H_1
H_2
PhCH
$PhCH_2$
H_5
PhCH
H_1'
H_6
PhCH
H_3 H_6
H_6'
PhCH
H_6'
H_4' H_5'
H_4 H_3'
H_2'
5.5
5.0
4.5
4.0
3.5
f1 (ppm)
$STolCH_3$
CH_3CO
1.99 0.99 2.06 3.96 1.03 4.20 2.17 17.20 2.05 2.03 1.00 1.03 1.03 1.04 2.07 1.06 1.01 1.03 1.04 2.08 2.10 1.05 1.99 2.17 1.03 3.04 3.07 9.14
0.0 9.5 9.0 8.5 8.0 7.5 7.0 6.5 6.0 5.5 5.0 4.5 4.0 3.5 3.0 2.5 2.0 1.5 1.0 0.5 0.0
f1 (ppm)

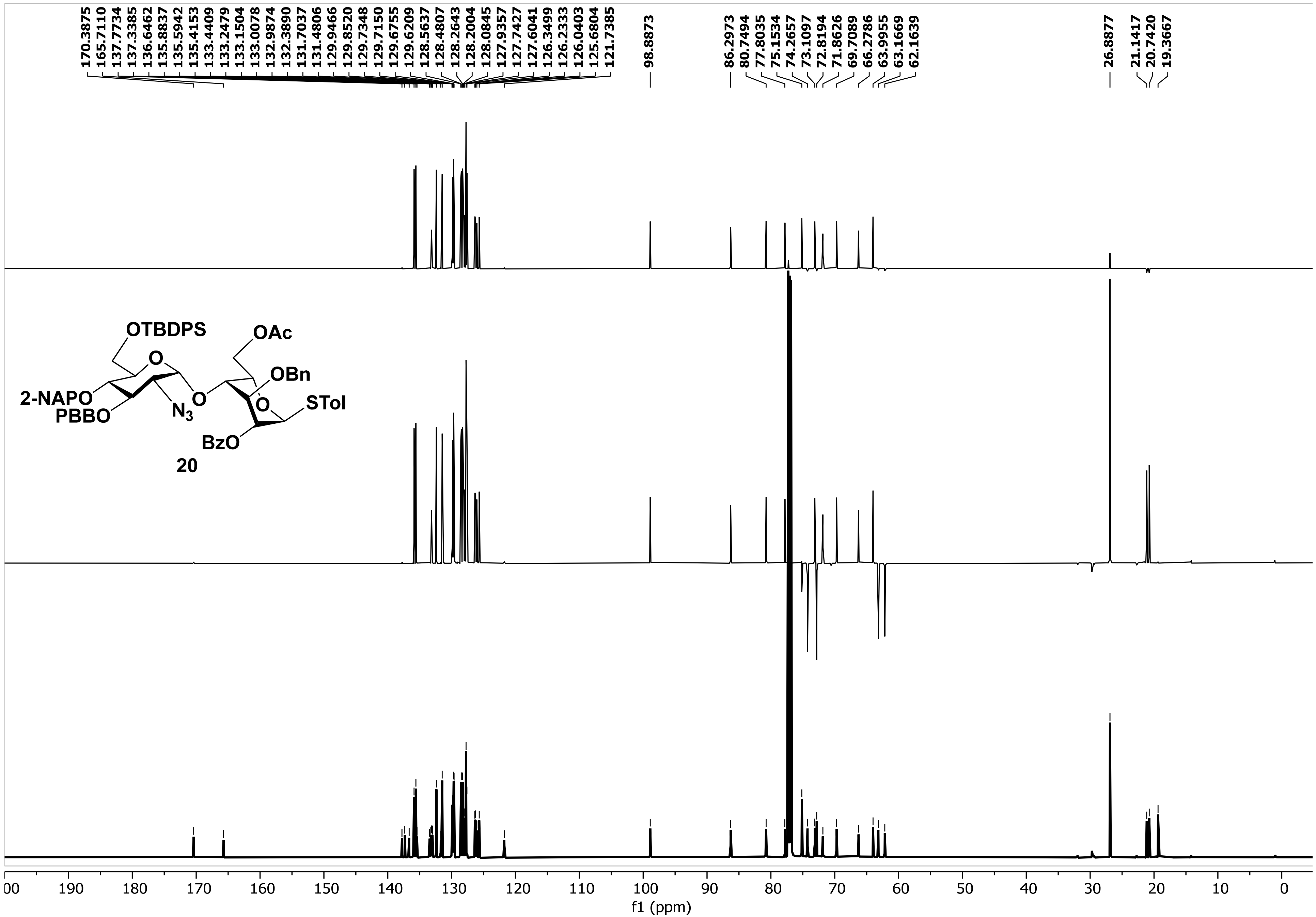
170.3875
165.7110
137.7734
137.3385
136.6462
135.8837
135.5942
135.4153
133.4409
133.2479
133.1504
133.0078
132.9874
132.3890
131.7037
131.4806
129.9466
129.8520
129.7348
129.7150
129.6755
129.6209
128.5637
128.4807
128.2643
128.2004
128.0845
127.9357
127.7427
127.6041
126.3499
126.2333
126.0403
125.6804
121.7385
98.8873
86.2973
80.7494
77.8035
75.1534
74.2657
73.1097
72.8194
71.8626
69.7089
66.2786
63.9955
63.1669
62.1639
26.8877
21.1417
20.7420
19.3667
OTBDPS
OAc
O
OBn
2-NAPO
O
O
STol
PBBO
N_3
BzO
20
f1 (ppm)

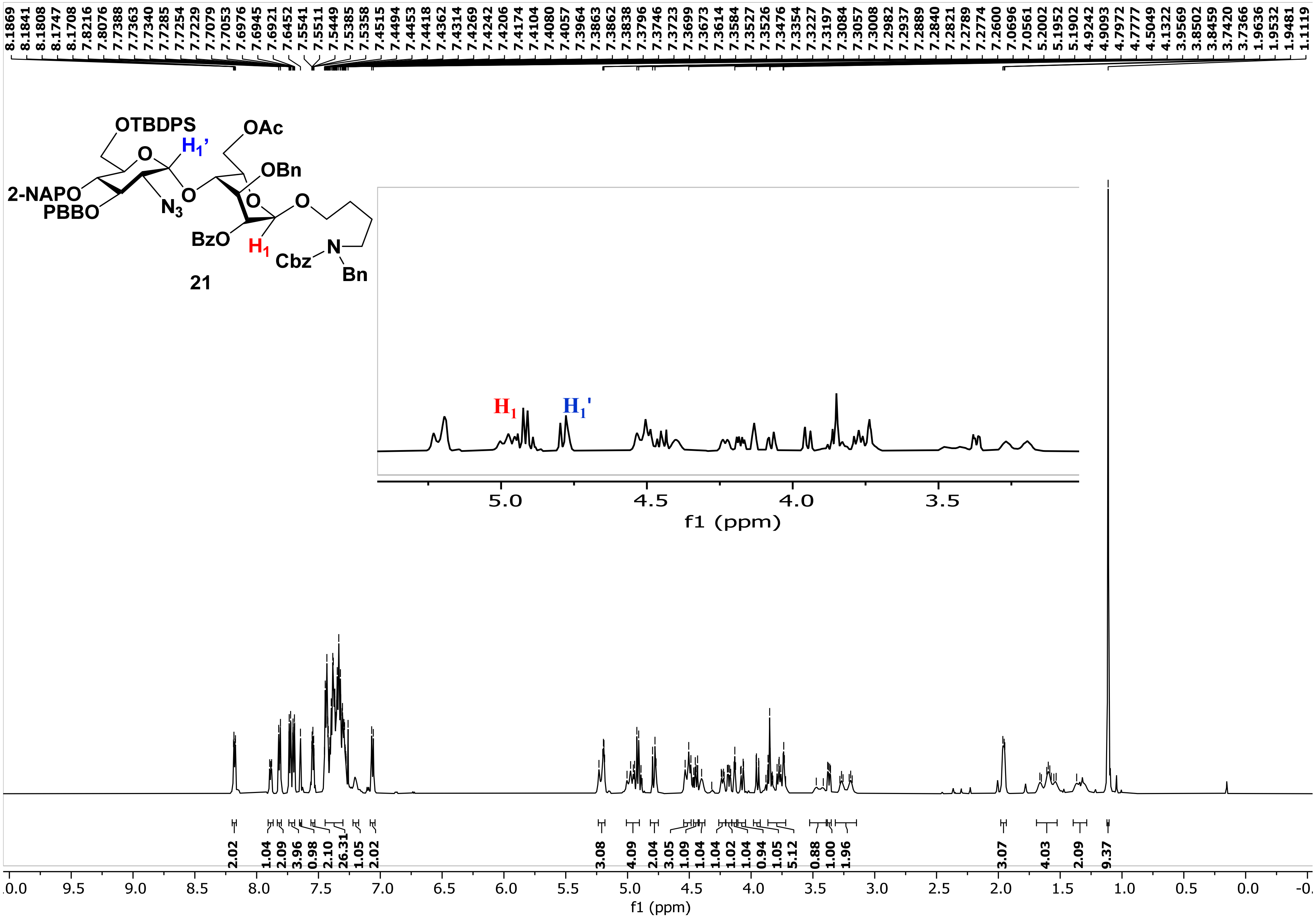

OTBDPS
OAc
H_1'
OBn
2-NAPO
PBBO
N_3
BzO
H_1
Cbz
N
Bn
21
H_1
H_1'
f1 (ppm)
f1 (ppm)

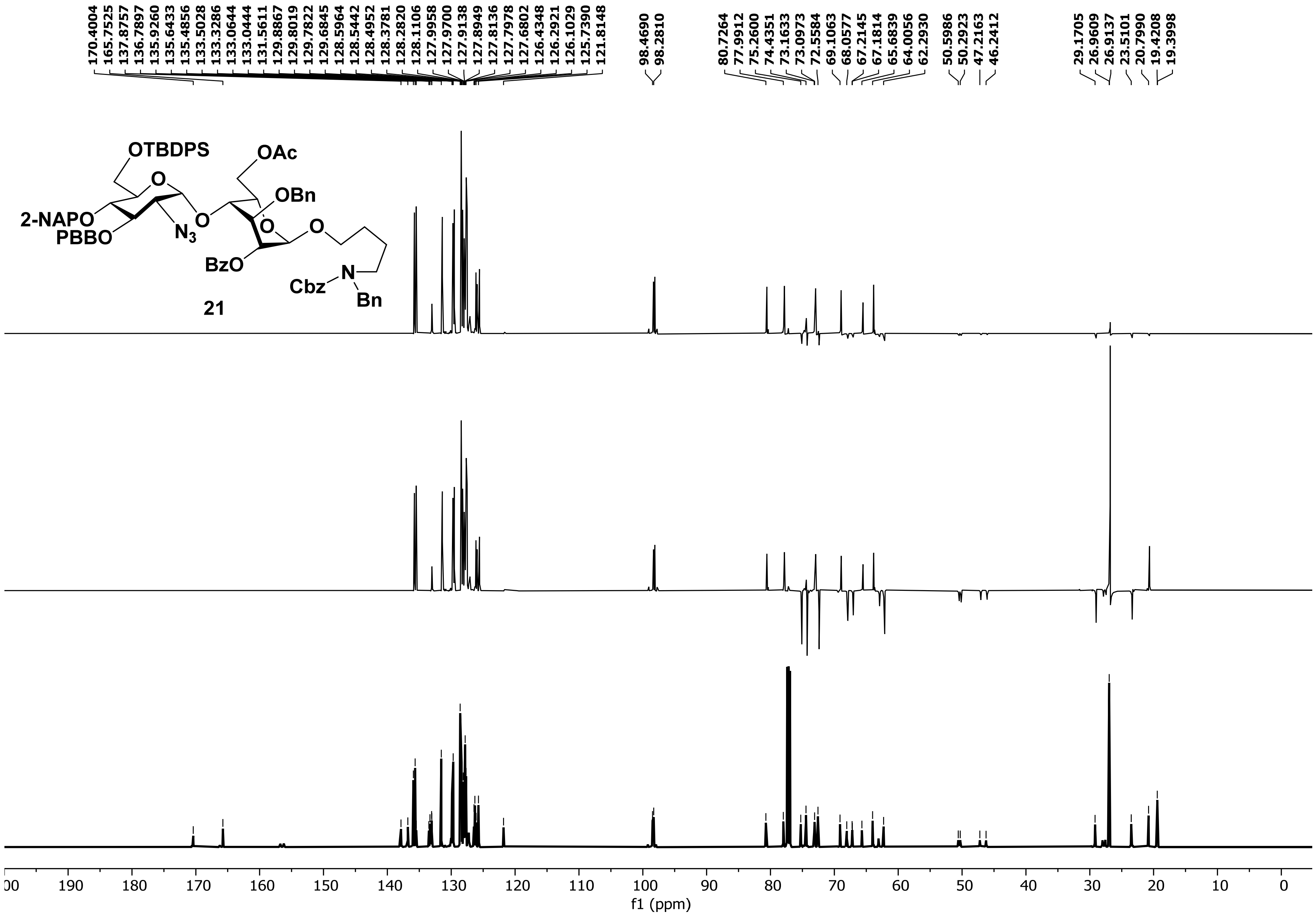
170.4004
165.7525
137.8757
136.7897
135.9260
135.6433
135.4856
133.5028
133.3286
133.0644
133.0444
131.5611
129.8867
129.8019
129.7822
129.6845
128.5964
128.5442
128.4952
128.3781
128.2820
128.1106
127.9958
127.9700
127.9138
127.8949
127.8136
127.7978
127.6802
126.4348
126.2921
126.1029
125.7390
121.8148
98.4690
98.2810
80.7264
77.9912
75.2600
74.4351
73.1633
73.0973
72.5584
69.1063
68.0577
67.2145
67.1814
65.6839
64.0056
62.2930
50.5986
50.2923
47.2163
46.2412
29.1705
26.9609
26.9137
23.5101
20.7990
19.4208
19.3998
OTBDPS
OAc
OBn
2-NAPO
PBBO
N_3
BzO
Cbz
N
Bn
21
f1 (ppm)

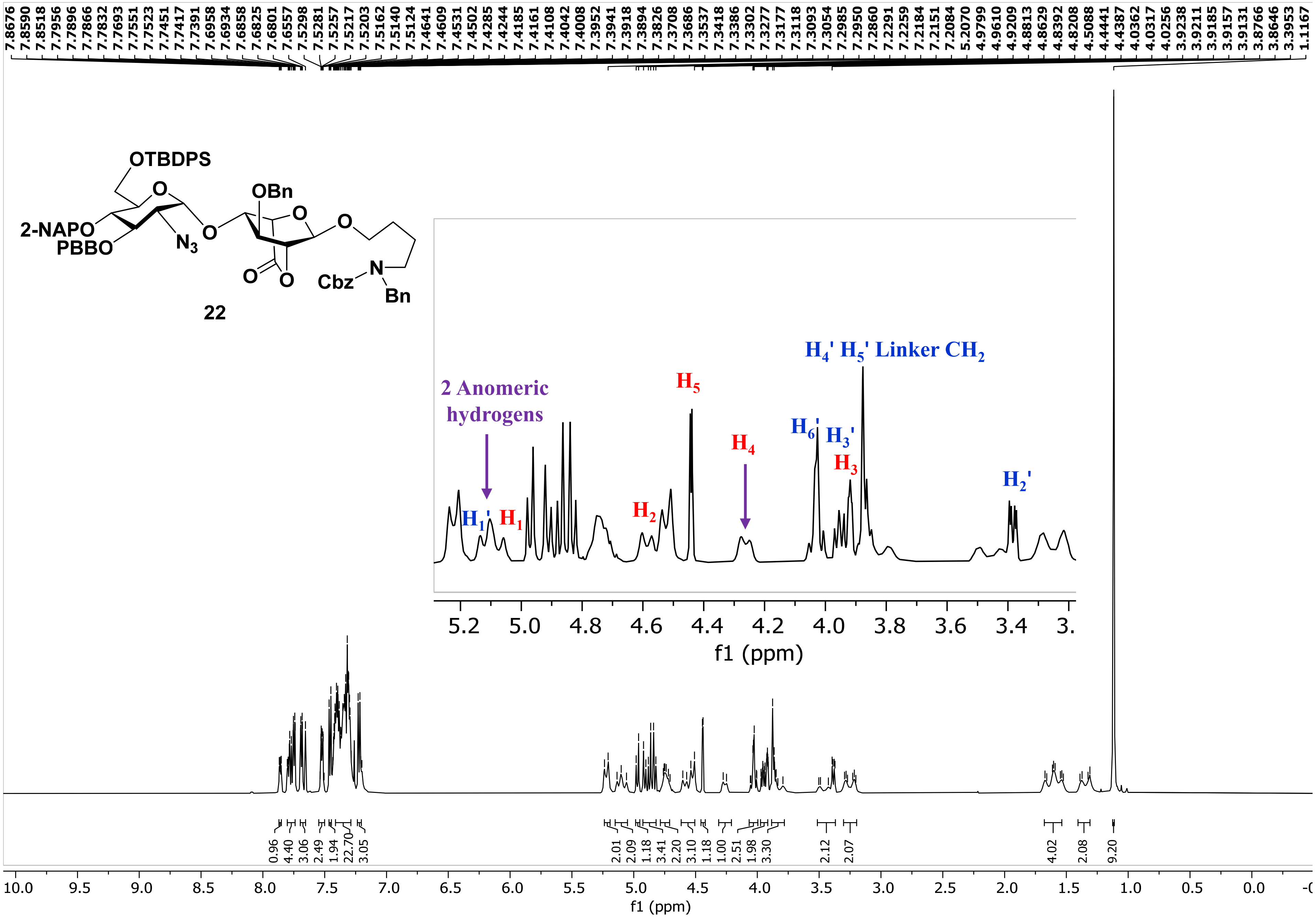
OTBDPS
2-NAPO
PBBO
N3
OBn
O
Cbz
N
Bn
22
2 Anomeric hydrogens
H1'
H1
H2
H5
H4
H6'
H3'
H3
H4' H5' Linker CH2
H2'
f1 (ppm)

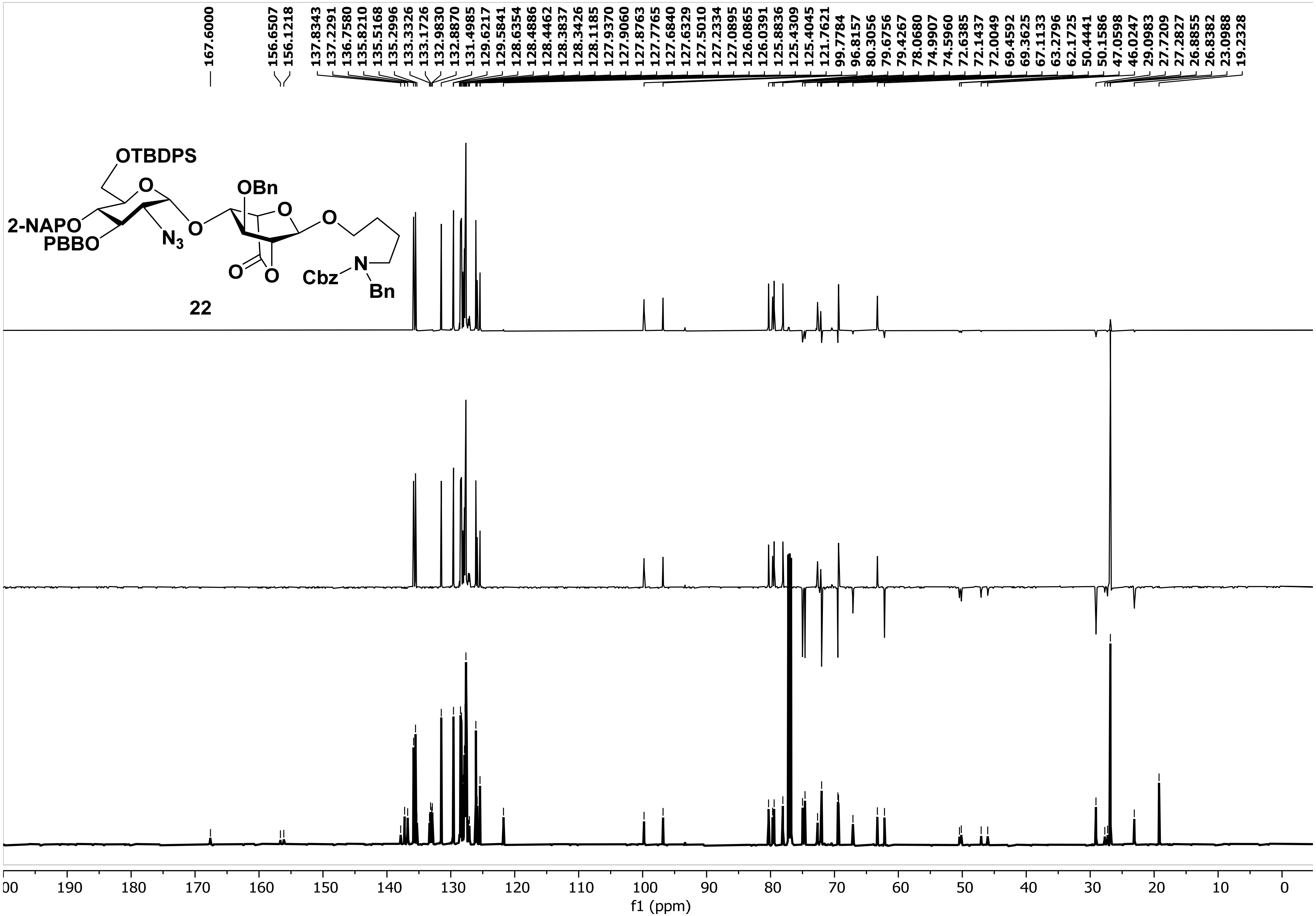

167.6000
156.6507
156.1218
137.8343
137.2291
136.7580
135.8210
135.5168
135.2996
133.3326
133.1726
132.9830
132.8870
131.4985
129.6217
129.5841
128.6354
128.4886
128.4462
128.3837
128.3426
128.1185
127.9370
127.9060
127.8763
127.7765
127.6840
127.6329
127.5010
127.2334
127.0895
126.0865
126.0391
125.8836
125.4309
125.4045
121.7621
99.7784
96.8157
80.3056
79.6756
79.4267
78.0680
74.9907
74.5960
72.6385
72.1437
72.0049
69.4592
69.3625
67.1133
63.2796
62.1725
50.4441
50.1586
47.0598
46.0247
29.0983
27.7209
27.2827
26.8855
26.8382
23.0988
19.2328
OTBDPS
O
2-NAPO
PBBO
N3
O
OBn
O
O
O
O
Cbz
N
Bn
22
190
180
170
160
150
140
130
120
110
100
90
80
70
60
50
40
30
20
10
0
f1 (ppm)

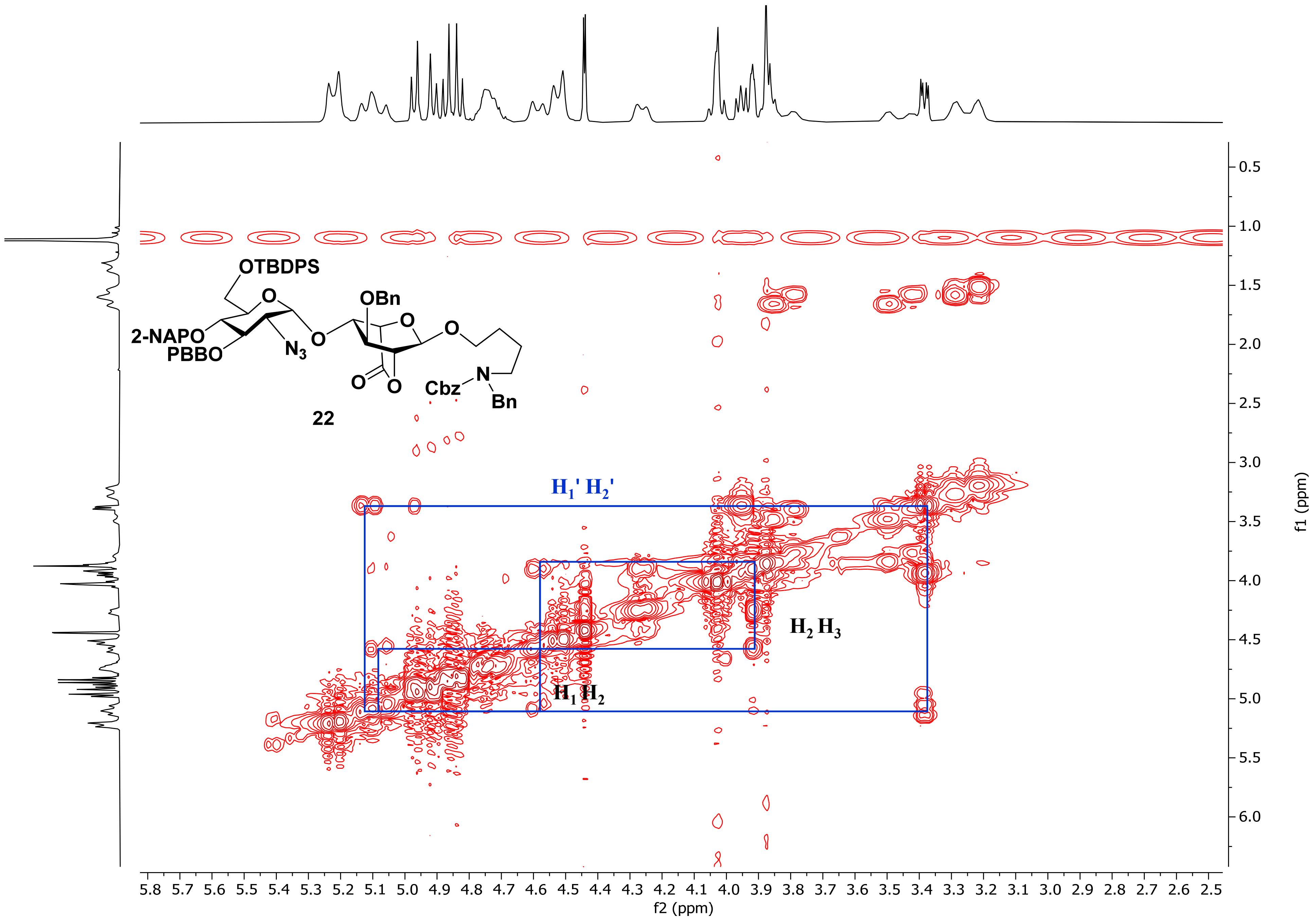
OTBDPS
OBn
2-NAPO
PBBO
N3
Cbz
N
Bn
22
H1' H2'
H2 H3
H1 H2
f2 (ppm)
f1 (ppm)

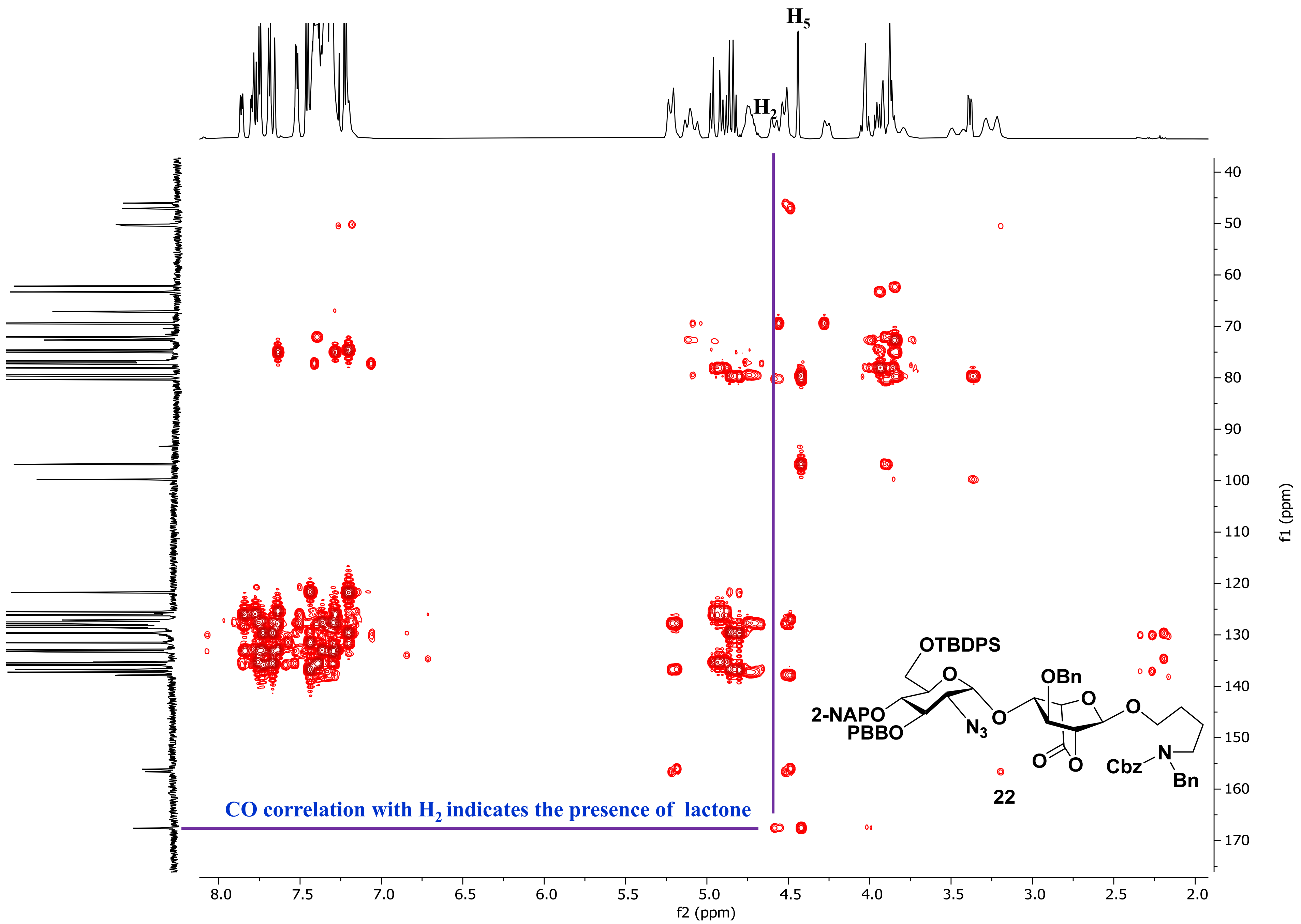

H5
H2
OTBDPS
OBn
2-NAPO
PBBO
N3
Cbz
N
Bn
22
CO correlation with H2 indicates the presence of lactone
f2 (ppm)
f1 (ppm)

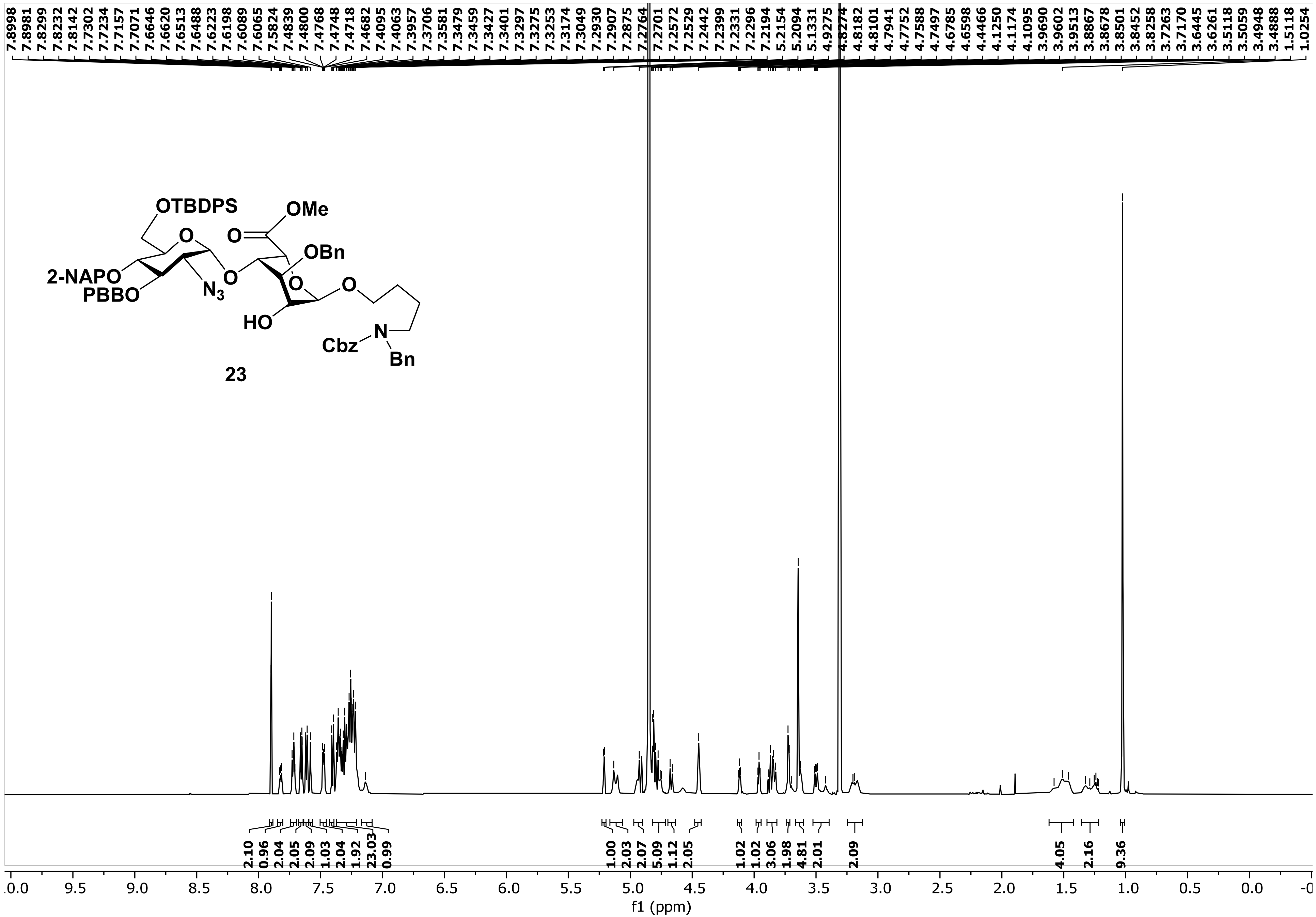

7.8998 7.8981 7.8299 7.8232 7.8142 7.7302 7.7234 7.7157 7.7071 7.6646 7.6620 7.6513 7.6488 7.6223 7.6198 7.6089 7.6065 7.5824 7.4839 7.4800 7.4768 7.4748 7.4718 7.4682 7.4095 7.4063 7.3957 7.3706 7.3581 7.3479 7.3459 7.3427 7.3401 7.3297 7.3275 7.3253 7.3174 7.3049 7.2930 7.2907 7.2875 7.2764 7.2701 7.2572 7.2529 7.2442 7.2399 7.2331 7.2296 7.2194 5.2154 5.2094 5.1331 4.9275 4.8274 4.8182 4.8101 4.7941 4.7752 4.7588 4.7497 4.6785 4.6598 4.4466 4.1250 4.1174 4.1095 3.9690 3.9602 3.9513 3.8867 3.8678 3.8501 3.8452 3.8258 3.7263 3.7170 3.6445 3.6261 3.5118 3.5059 3.4948 3.4888 1.5118 1.0254
OTBDPS
OMe
O
O
OBn
2-NAPO
PBBO
N3
O
O
O
HO
Cbz
N
Bn
23
2.10 0.96 2.04 2.05 2.09 1.03 2.04 1.92 23.03 0.99
1.00 2.03 2.07 5.09 1.12 2.05
1.02 1.02 3.06 1.98 4.81 2.01
2.09
4.05 2.16 9.36
.0.0 9.5 9.0 8.5 8.0 7.5 7.0 6.5 6.0 5.5 5.0 4.5 4.0 3.5 3.0 2.5 2.0 1.5 1.0 0.5 0.0 -0
f1 (ppm)

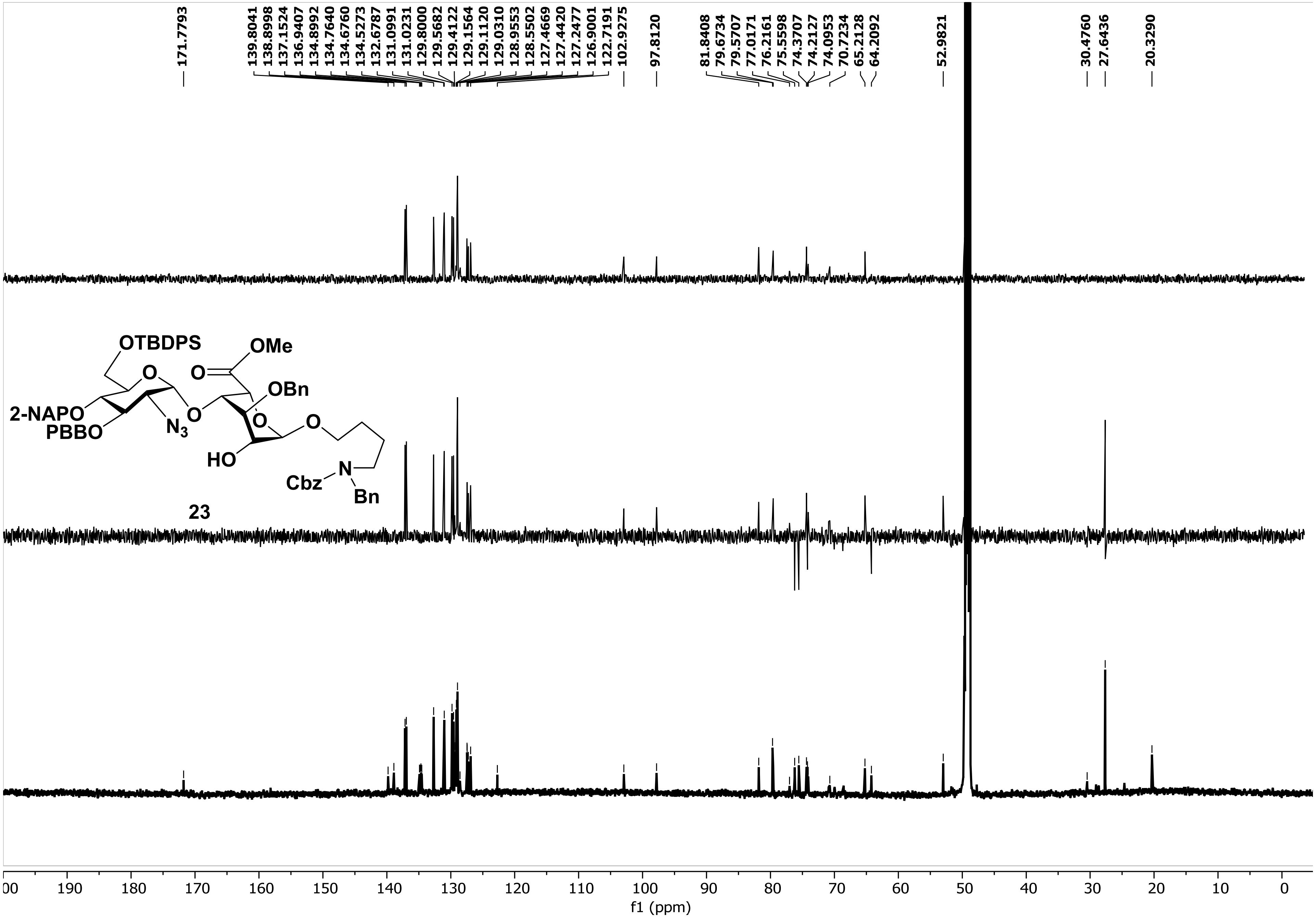
171.7793
139.8041
138.8998
137.1524
136.9407
134.8992
134.7640
134.6760
134.5273
132.6787
131.0991
131.0231
129.8000
129.5682
129.4122
129.1564
129.1120
129.0310
128.9553
128.5502
127.4669
127.4420
127.2477
126.9001
122.7191
102.9275
97.8120
81.8408
79.6734
79.5707
77.0171
76.2161
75.5598
74.3707
74.2127
74.0953
70.7234
65.2128
64.2092
52.9821
30.4760
27.6436
20.3290
OTBDPS
OMe
O
OBn
2-NAPO
PBBO
N_3
HO
Cbz
N
Bn
23
00 190 180 170 160 150 140 130 120 110 100 90 80 70 60 50 40 30 20 10 0
f1 (ppm)

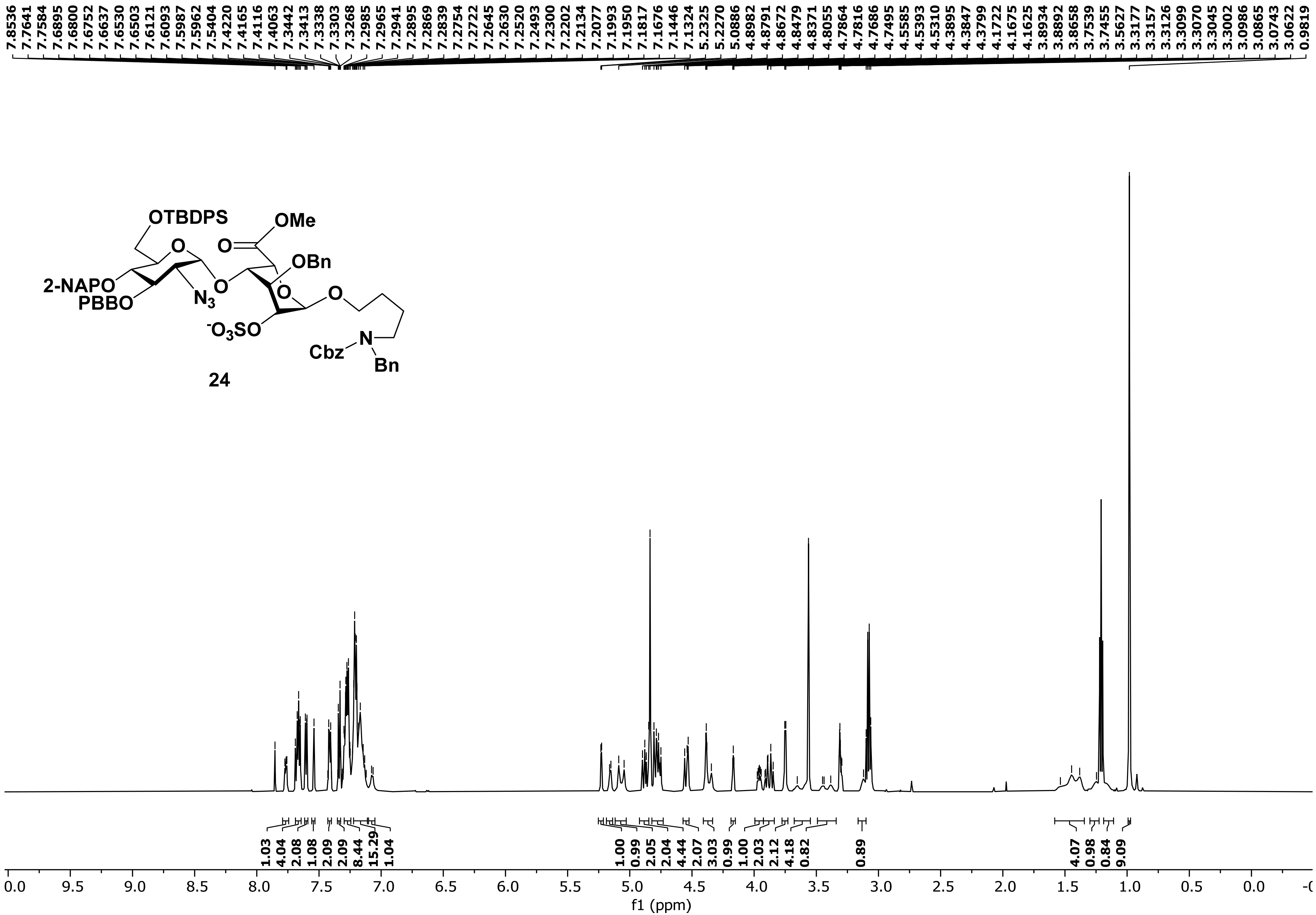
7.8536 7.7641 7.7584 7.6895 7.6800 7.6752 7.6637 7.6530 7.6503 7.6121 7.6093 7.5987 7.5962 7.5404 7.4220 7.4165 7.4116 7.4063 7.3442 7.3413 7.3338 7.3303 7.3268 7.2985 7.2965 7.2941 7.2895 7.2869 7.2839 7.2754 7.2722 7.2645 7.2630 7.2520 7.2493 7.2300 7.2202 7.2134 7.2077 7.1993 7.1950 7.1817 7.1676 7.1446 7.1324 5.2325 5.2270 5.0886 4.8982 4.8791 4.8672 4.8479 4.8371 4.8055 4.7864 4.7816 4.7686 4.7495 4.5585 4.5393 4.5310 4.3895 4.3847 4.3799 4.1722 4.1675 4.1625 3.8934 3.8892 3.8658 3.7539 3.7455 3.5627 3.3177 3.3157 3.3126 3.3099 3.3070 3.3045 3.3002 3.0986 3.0865 3.0743 3.0622 0.9819
OTBDPS
OMe
O
O
OBn
2-NAPO
PBBO
N3
O
O
O
-O3SO
N
Cbz
Bn
24
1.03 4.04 2.08 1.08 2.09 2.09 8.44 15.29 1.04
1.00 0.99 2.05 2.04 4.44 2.07 3.03 0.99 1.00 2.03 2.12 4.18 0.82
0.89
4.07 0.98 0.84 9.09
f1 (ppm)

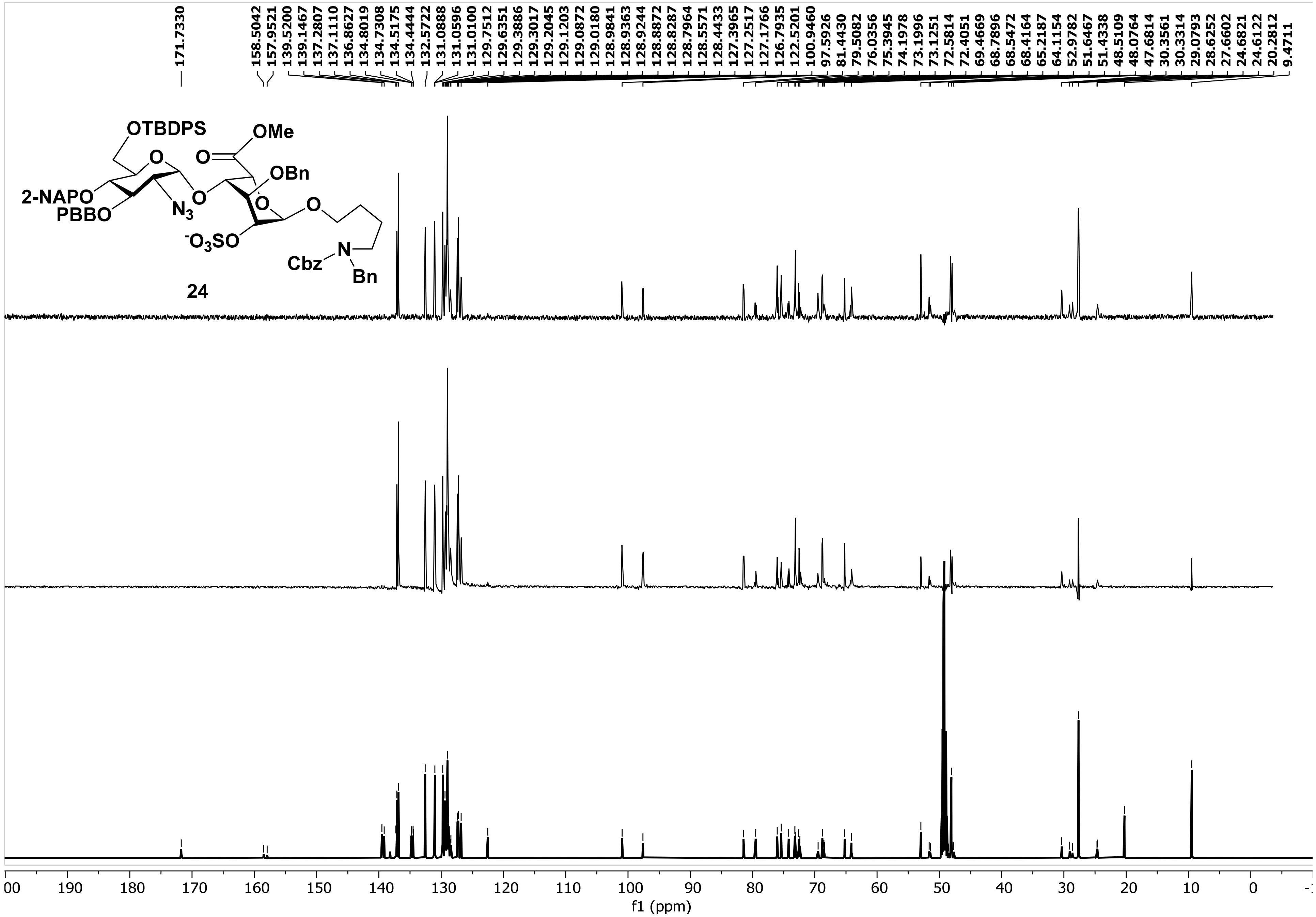
171.7330
158.5042
157.9521
139.5200
139.1467
137.2807
137.1110
136.8627
134.8019
134.7308
134.5175
134.4444
132.5722
131.0888
131.0596
131.0100
129.7512
129.6351
129.3886
129.3017
129.2045
129.1203
129.0872
129.0180
128.9841
128.9363
128.9244
128.8872
128.8287
128.7964
128.5571
128.4433
127.3965
127.2517
127.1766
126.7935
122.5201
100.9460
97.5926
81.4430
79.5082
76.0356
75.3945
74.1978
73.1996
73.1251
72.5814
72.4051
69.4669
68.7896
68.5472
68.4164
65.2187
64.1154
52.9782
51.6467
51.4338
48.5109
48.0764
47.6814
30.3561
30.3314
29.0793
28.6252
27.6602
24.6821
24.6122
20.2812
9.4711
OTBDPS
OMe
O
O
OBn
2-NAPO
O
O
PBBO
N_3
O
O
$^{-}O_3SO$
N
Cbz
Bn
24
f1 (ppm)

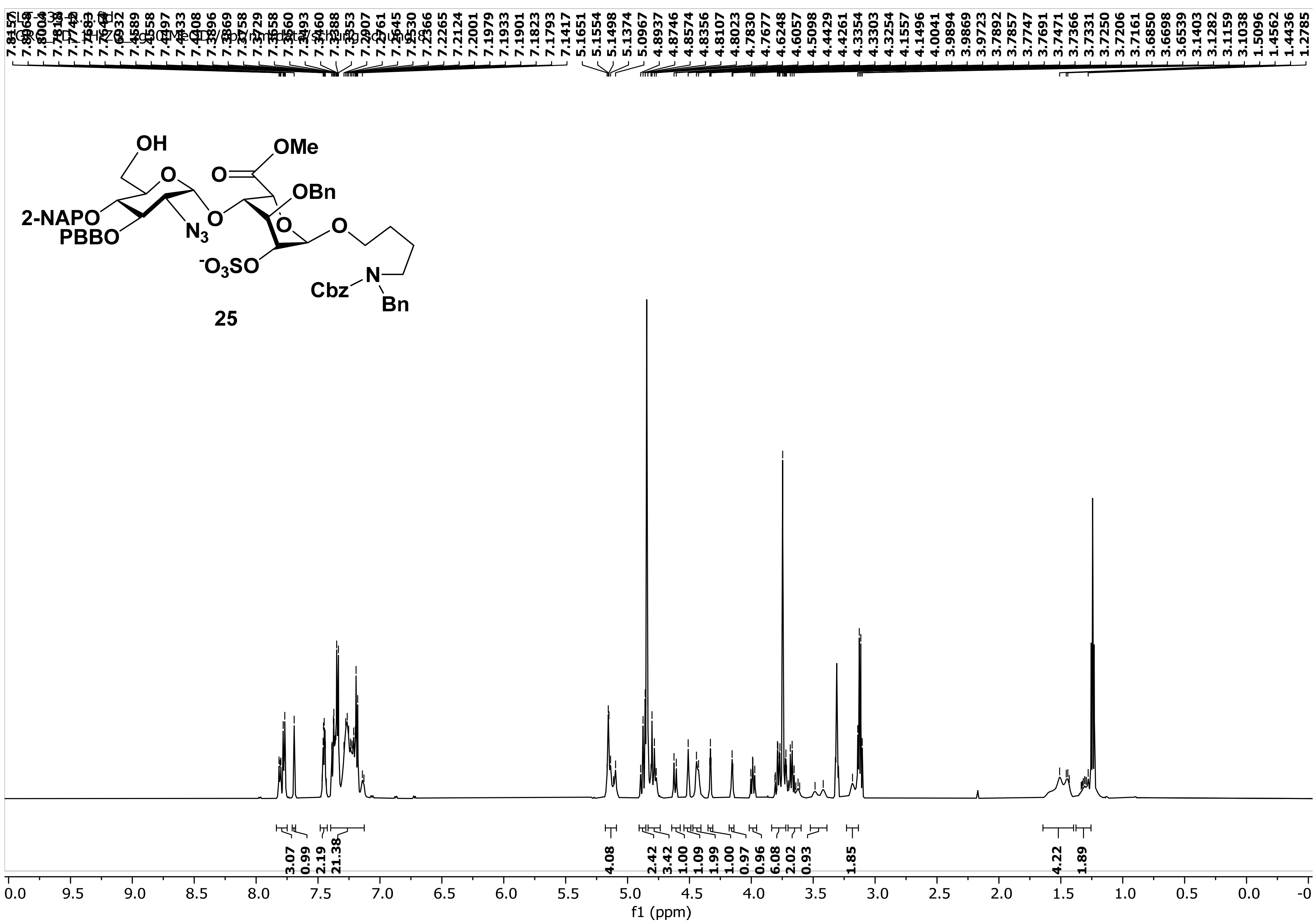
OH
O
OMe
OBn
2-NAPO
PBBO
N_3
^-O_3SO
Cbz
N
Bn
25
f1 (ppm)

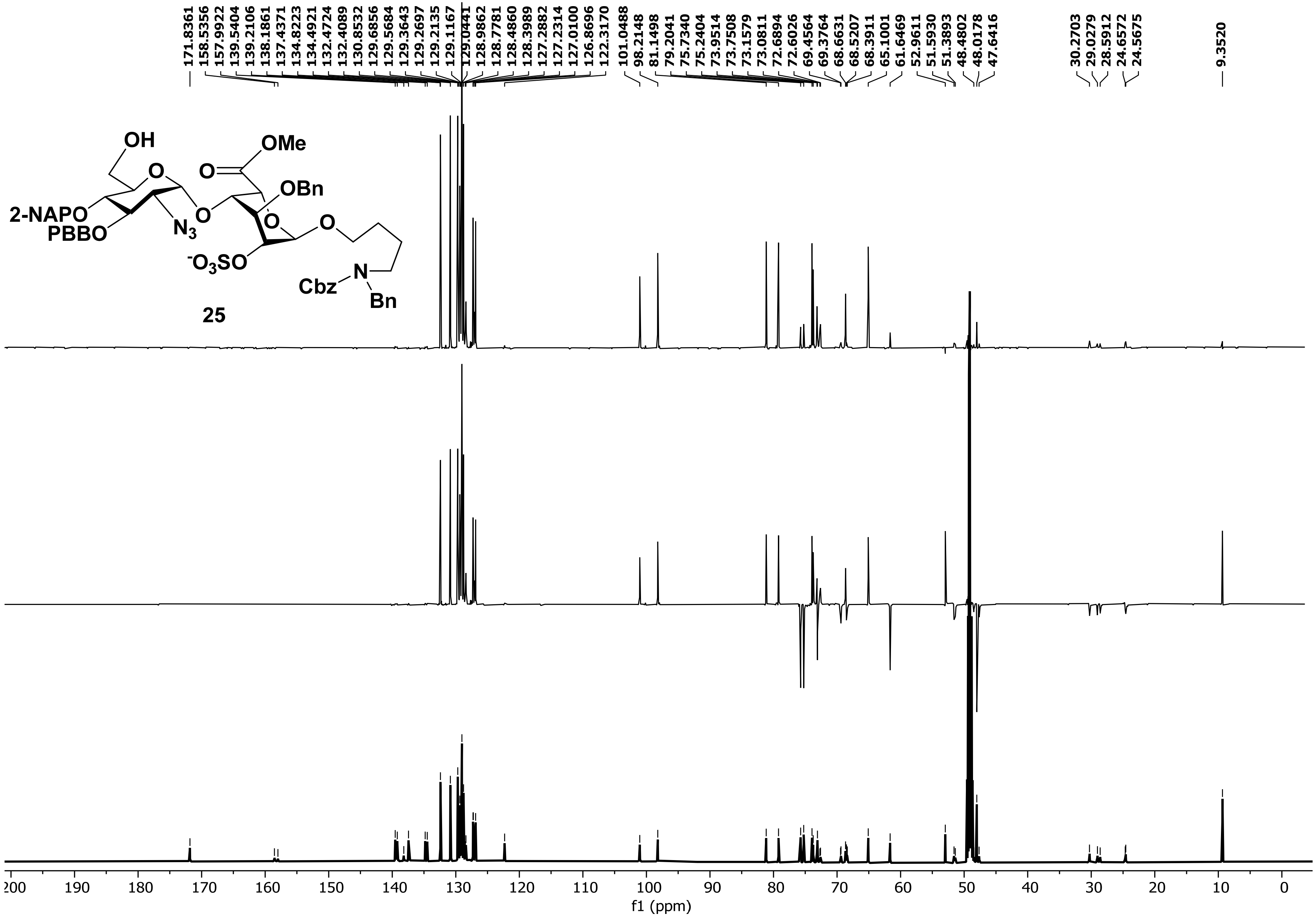
171.8361
158.5356
157.9922
139.5404
139.2106
138.1861
137.4371
134.8223
134.4921
132.4724
132.4089
130.8532
129.6856
129.5684
129.3643
129.2697
129.2135
129.1167
129.0441
128.9862
128.7781
128.4860
128.3989
127.2882
127.2314
127.0100
126.8696
122.3170
101.0488
98.2148
81.1498
79.2041
75.7340
75.2404
73.9514
73.7508
73.1579
73.0811
72.6894
72.6026
69.4564
69.3764
68.6631
68.5207
68.3911
65.1001
61.6469
52.9611
51.5930
51.3893
48.4802
48.0178
47.6416
30.2703
29.0279
28.5912
24.6572
24.5675
9.3520
OH
O
OMe
O
OBn
2-NAPO
O
PBBO
N_3
O
O
$^{-}O_3SO$
N
Cbz
Bn
25
f1 (ppm)

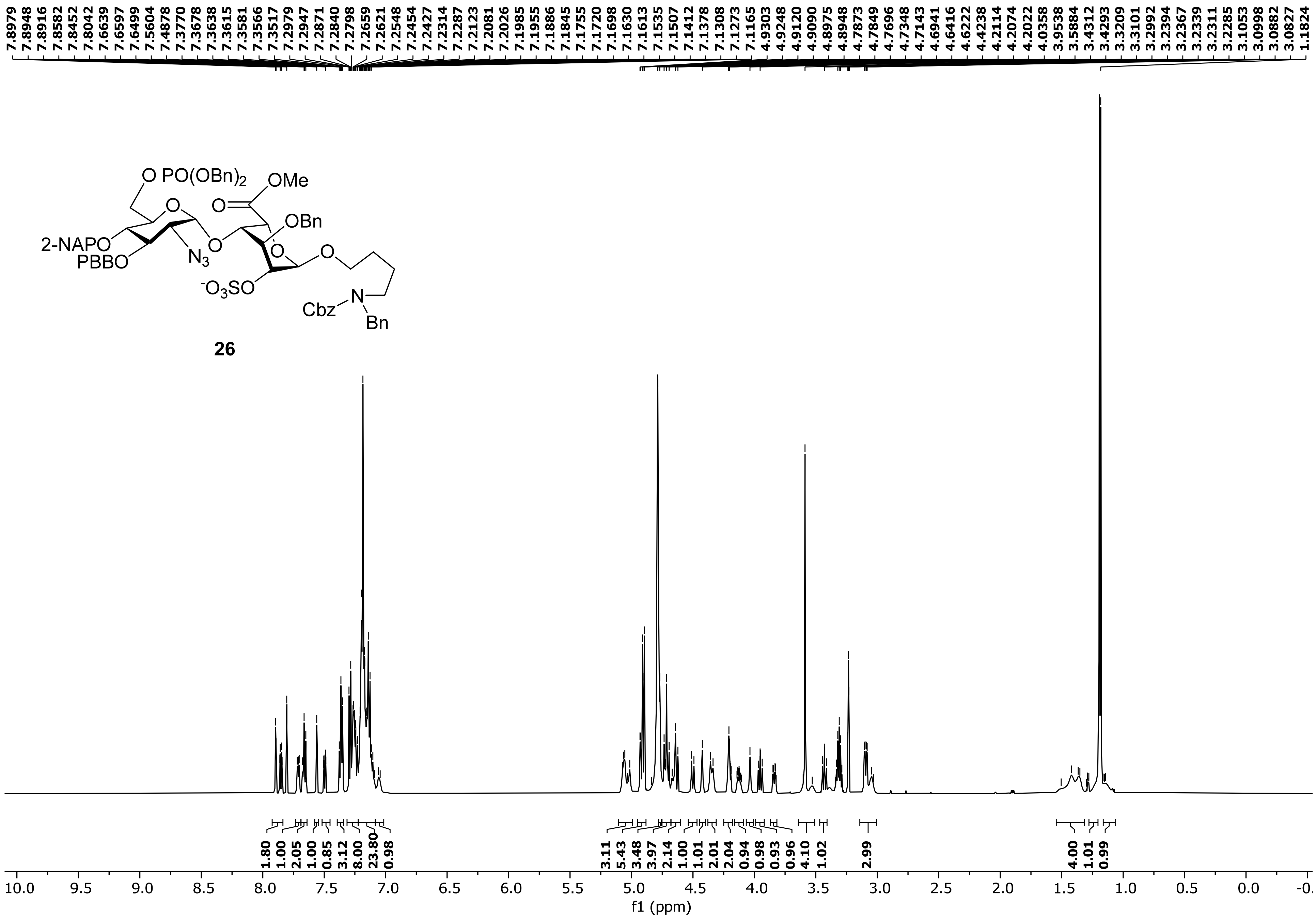
7.8979 7.8948 7.8916 7.8582 7.8452 7.8042 7.6639 7.6597 7.6499 7.5604 7.4878 7.3770 7.3678 7.3638 7.3615 7.3581 7.3566 7.3517 7.2979 7.2947 7.2871 7.2840 7.2798 7.2659 7.2621 7.2548 7.2454 7.2427 7.2314 7.2287 7.2123 7.2081 7.2026 7.1985 7.1955 7.1886 7.1845 7.1755 7.1720 7.1698 7.1630 7.1613 7.1535 7.1507 7.1412 7.1378 7.1308 7.1273 7.1165 4.9303 4.9248 4.9120 4.9090 4.8975 4.8948 4.7873 4.7849 4.7696 4.7348 4.7143 4.6941 4.6416 4.6222 4.4238 4.2114 4.2074 4.2022 4.0358 3.9538 3.5884 3.4312 3.4293 3.3209 3.3101 3.2992 3.2394 3.2367 3.2339 3.2311 3.2285 3.1053 3.0998 3.0882 3.0827 1.1824
O PO(OBn)2
OMe
O
O
OBn
2-NAPO
PBBO
N3
O
O
O
-O3SO
Cbz
N
Bn
26
1.80 1.00 2.05 1.00 0.85 3.12 8.00 23.80 0.98
3.11 5.43 3.48 3.97 2.14 1.00 1.01 2.01 2.04 0.94 0.98 0.93 0.96 4.10 1.02 2.99
4.00 1.01 0.99
10.0 9.5 9.0 8.5 8.0 7.5 7.0 6.5 6.0 5.5 5.0 4.5 4.0 3.5 3.0 2.5 2.0 1.5 1.0 0.5 0.0
f1 (ppm)

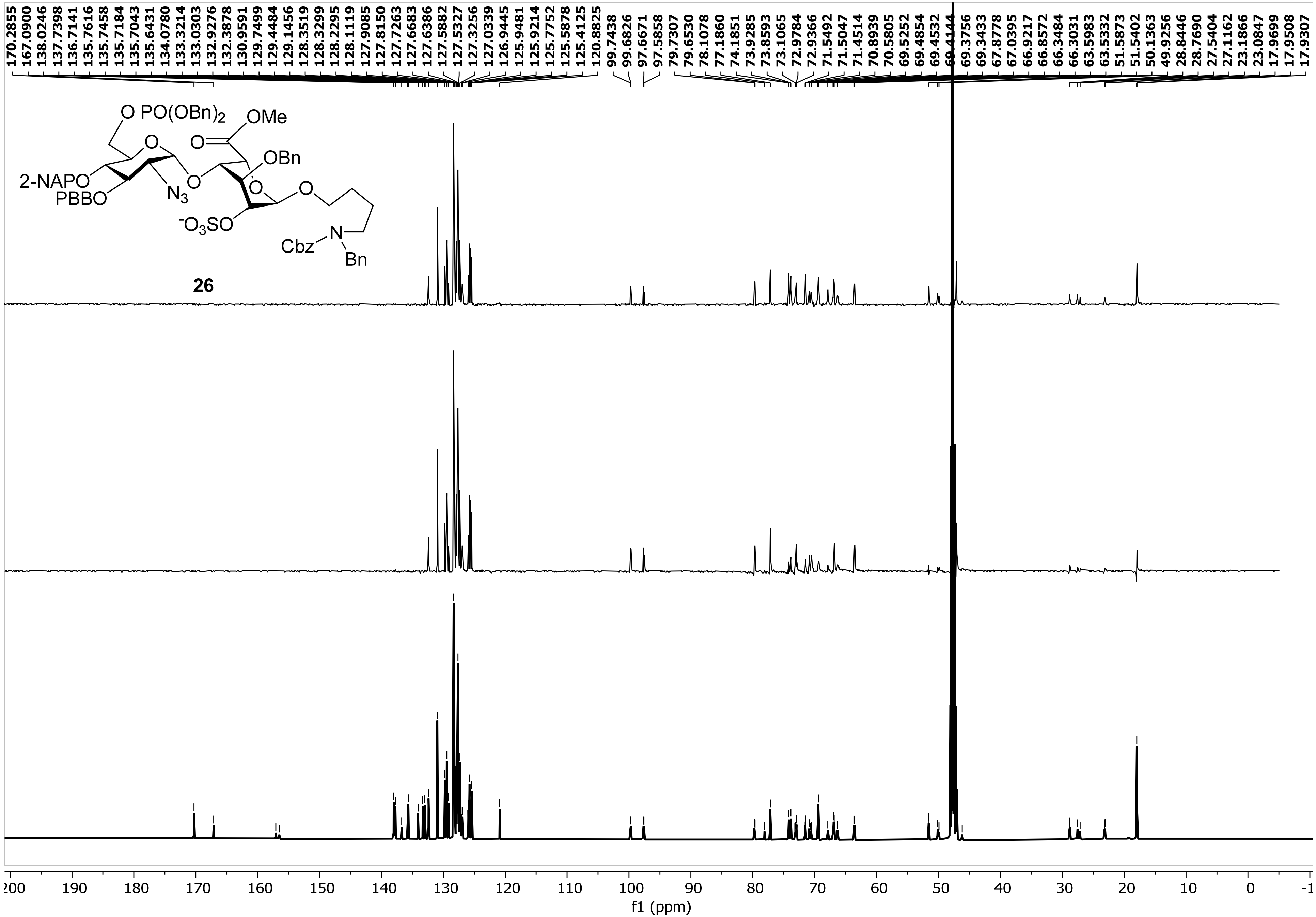
170.2855
167.0900
138.0246
137.7398
136.7141
135.7616
135.7458
135.7184
135.7043
135.6431
134.0780
133.3214
133.0303
132.9276
132.3878
130.9591
129.7499
129.4484
129.1456
128.3519
128.3299
128.2295
128.1119
127.9085
127.8150
127.7263
127.6683
127.6386
127.5882
127.5327
127.3256
127.0339
126.9445
125.9481
125.9214
125.7752
125.5878
125.4125
120.8825
99.7438
99.6826
97.6671
97.5858
79.7307
79.6530
78.1078
77.1860
74.1851
73.9285
73.8593
73.1065
72.9784
72.9366
71.5492
71.5047
71.4514
70.8939
70.5805
69.5252
69.4854
69.4532
69.4144
69.3756
69.3433
67.8778
67.0395
66.9217
66.8572
66.3484
66.3031
63.5983
63.5332
51.5873
51.5402
50.1363
49.9256
28.8446
28.7690
27.5404
27.1162
23.1866
23.0847
17.9699
17.9508
17.9307
O PO(OBn)$_2$
OMe
O
O
OBn
2-NAPO
PBBO
N_3
O
O
O
^-O_3SO
Cbz
N
Bn
26
200
190
180
170
160
150
140
130
120
110
100
90
80
70
60
50
40
30
20
10
0
-10
f1 (ppm)

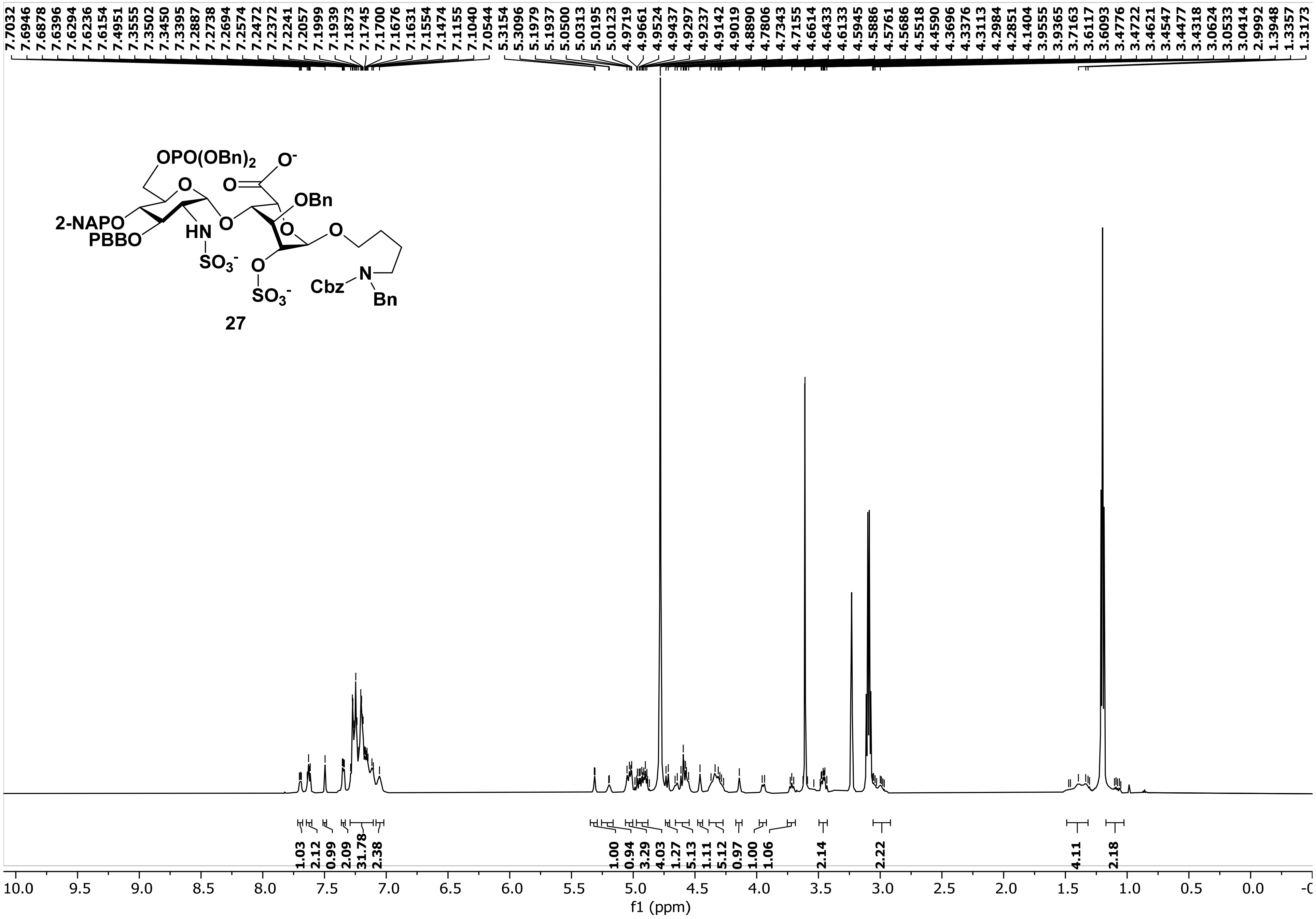
OPO(OBn)2
2-NAPO
PBBO
HN
SO3-
OBn
O-
27
Cbz
N
Bn
f1 (ppm)

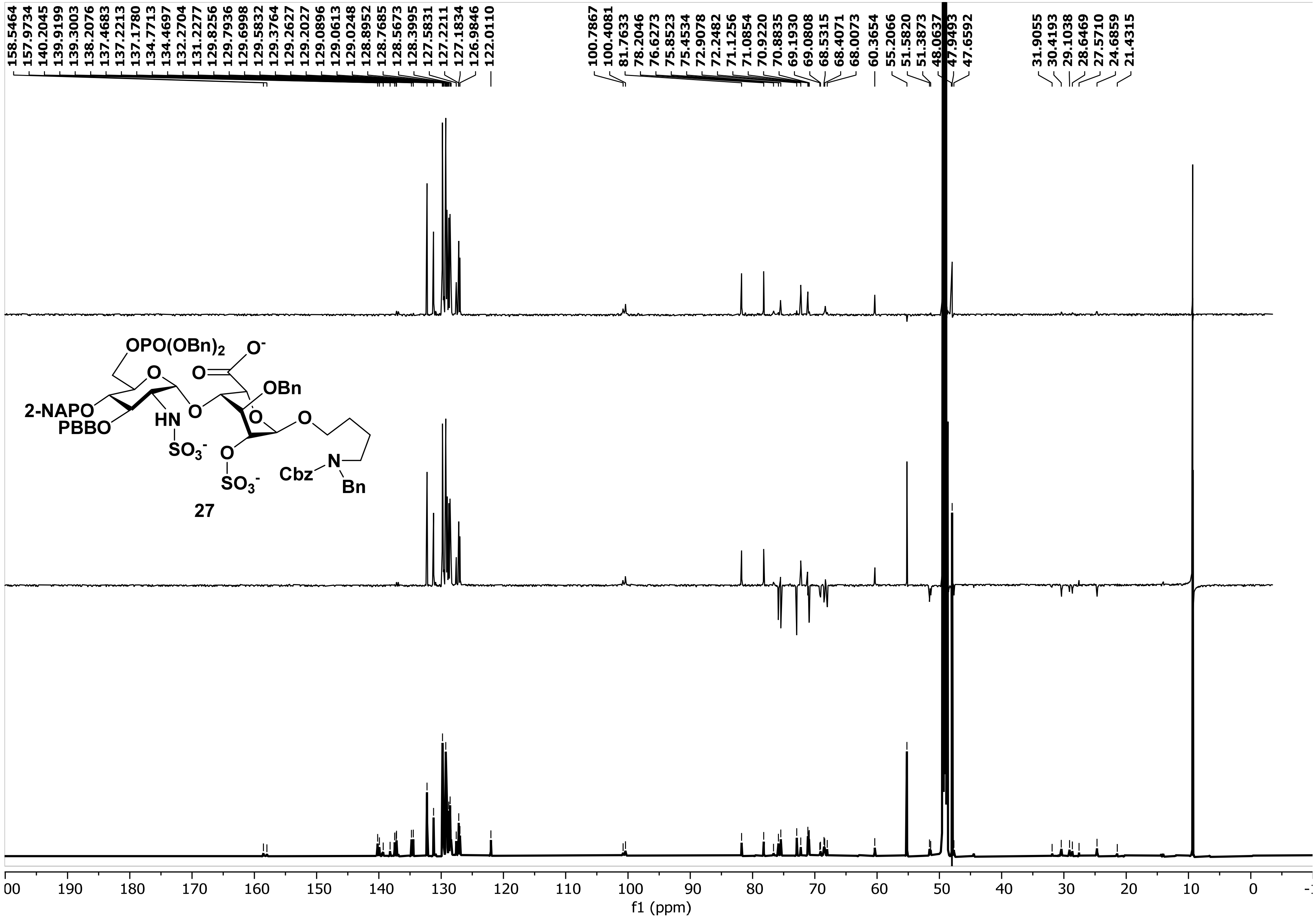
158.5464
157.9734
140.2045
139.9199
139.3003
138.2076
137.4683
137.2213
137.1780
134.7713
134.4697
132.2704
131.2277
129.8256
129.7936
129.6998
129.5832
129.3764
129.2627
129.2027
129.0896
129.0613
129.0248
128.8952
128.7685
128.5673
128.3995
127.5831
127.2211
127.1834
126.9846
122.0110
100.7867
100.4081
81.7633
78.2046
76.6273
75.8523
75.4534
72.9078
72.2482
71.1256
71.0854
70.9220
70.8835
69.1930
69.0808
68.5315
68.4071
68.0073
60.3654
55.2066
51.5820
51.3873
48.0637
47.9493
47.6592
31.9055
30.4193
29.1038
28.6469
27.5710
24.6859
21.4315
OPO(OBn)2
O-
O
O
OBn
2-NAPO
PBBO
HN
O
O
O
SO3-
O
SO3-
Cbz
N
Bn
27
200
190
180
170
160
150
140
130
120
110
100
90
80
70
60
50
40
30
20
10
0
-10
f1 (ppm)

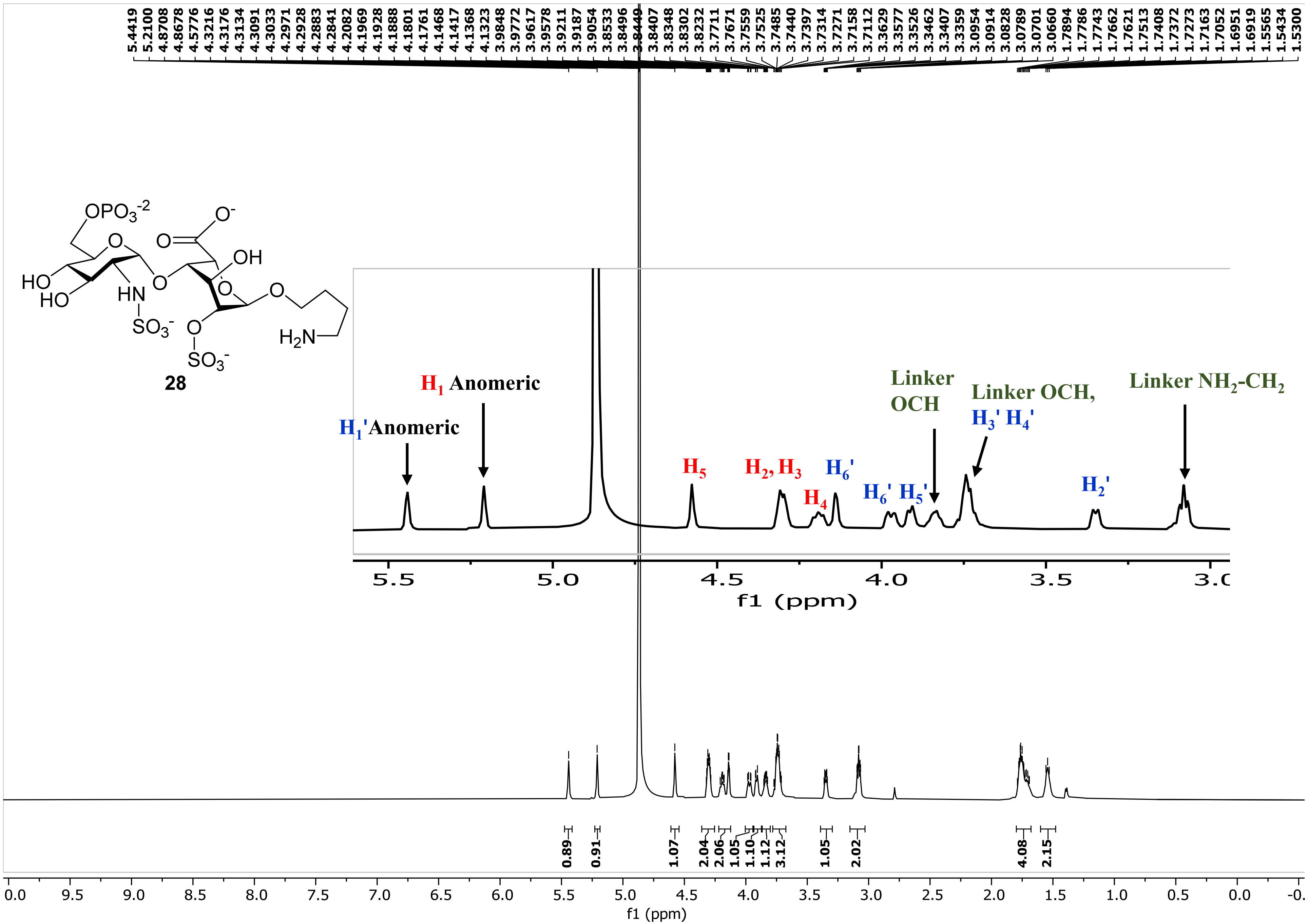

5.4419 5.2100 4.8708 4.8678 4.5776 4.3216 4.3176 4.3134 4.3091 4.3033 4.2971 4.2928 4.2883 4.2841 4.2082 4.1969 4.1928 4.1888 4.1801 4.1761 4.1468 4.1417 4.1368 4.1323 3.9848 3.9772 3.9617 3.9578 3.9211 3.9187 3.9054 3.8533 3.8496 3.8449 3.8407 3.8348 3.8302 3.8232 3.7711 3.7671 3.7559 3.7525 3.7485 3.7440 3.7397 3.7314 3.7271 3.7158 3.7112 3.3629 3.3577 3.3526 3.3462 3.3407 3.3359 3.0954 3.0914 3.0828 3.0789 3.0701 3.0660 1.7894 1.7786 1.7743 1.7662 1.7621 1.7513 1.7408 1.7372 1.7273 1.7163 1.7052 1.6951 1.6919 1.5565 1.5434 1.5300
28
H1'Anomeric
H1 Anomeric
H5
H2, H3
H4
H6'
H6' H5'
Linker OCH
Linker OCH, H3' H4'
H2'
Linker NH2-CH2
5.5 5.0 4.5 4.0 3.5 3.0
f1 (ppm)
0.89 0.91 1.07 2.04 2.06 1.05 1.10 1.12 3.12 1.05 2.02 4.08 2.15
0.0 9.5 9.0 8.5 8.0 7.5 7.0 6.5 6.0 5.5 5.0 4.5 4.0 3.5 3.0 2.5 2.0 1.5 1.0 0.5 0.0 -0.
f1 (ppm)

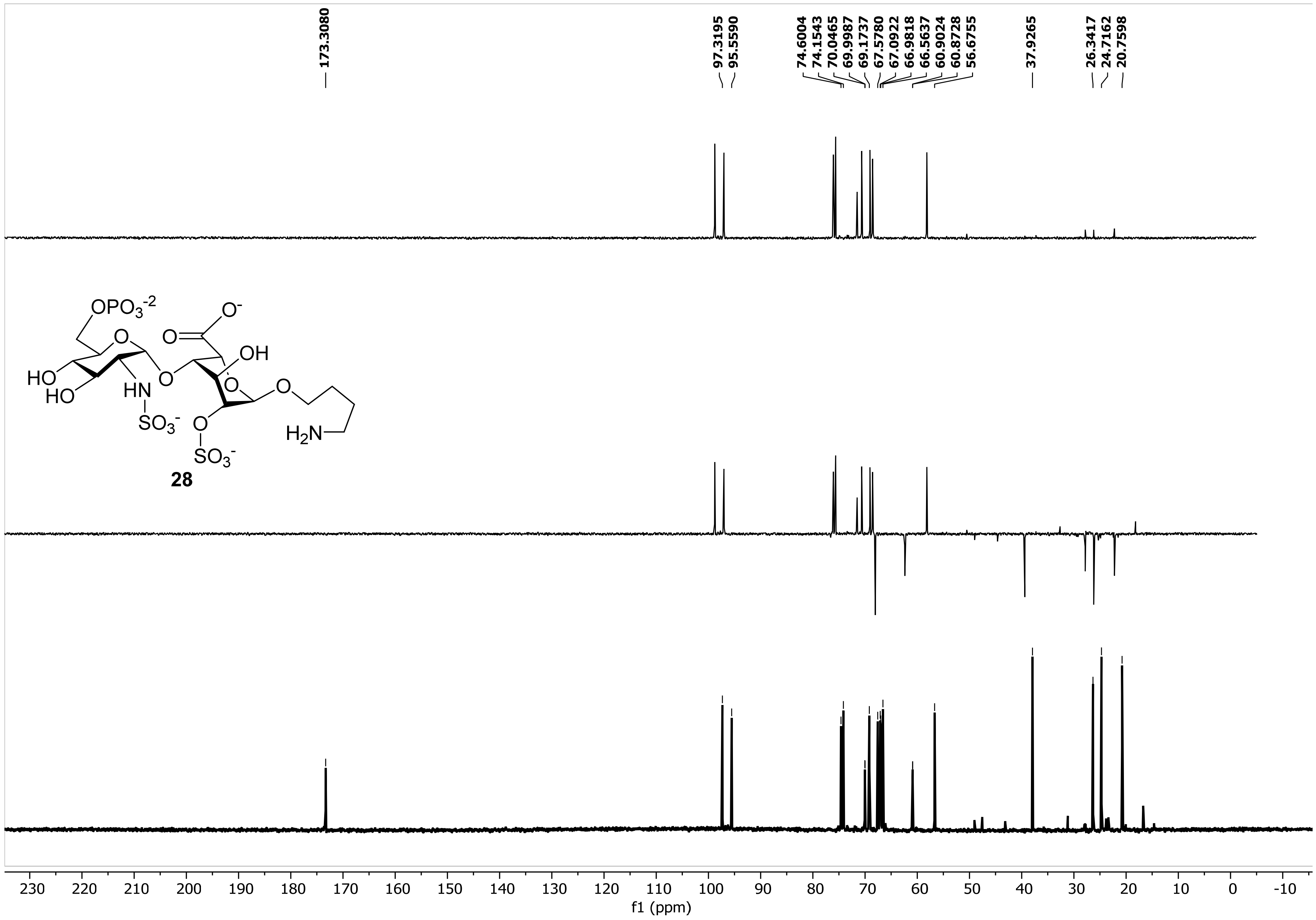

173.3080
97.3195
95.5590
74.6004
74.1543
70.0465
69.9987
69.1737
67.5780
67.0922
66.9818
66.5637
60.9024
60.8728
56.6755
37.9265
26.3417
24.7162
20.7598
28
f1 (ppm)

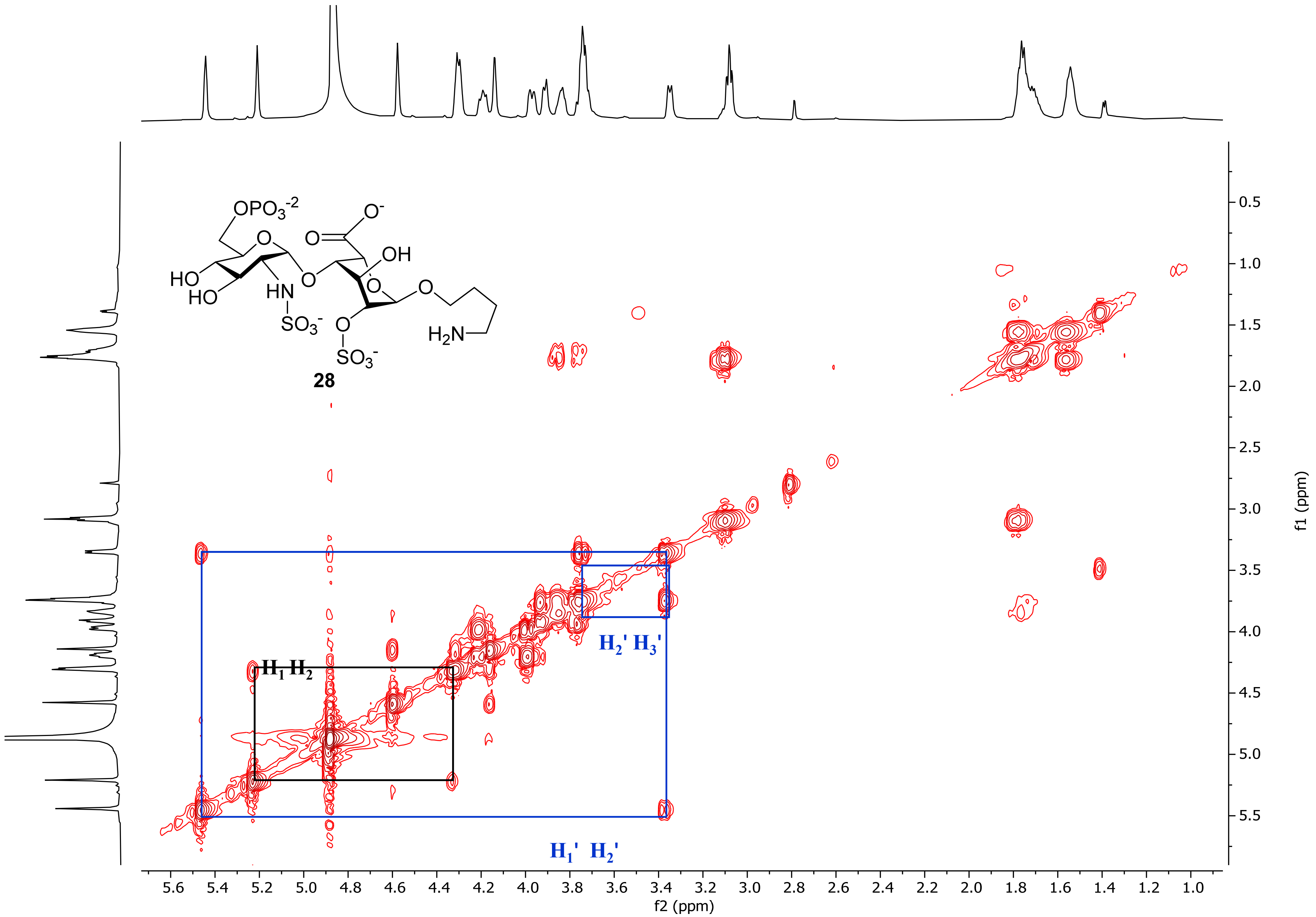
OPO3-2
O-
OH
HO
HO
HN
SO3-
SO3-
H2N
28
H1 H2
H2' H3'
H1' H2'
f1 (ppm)
f2 (ppm)

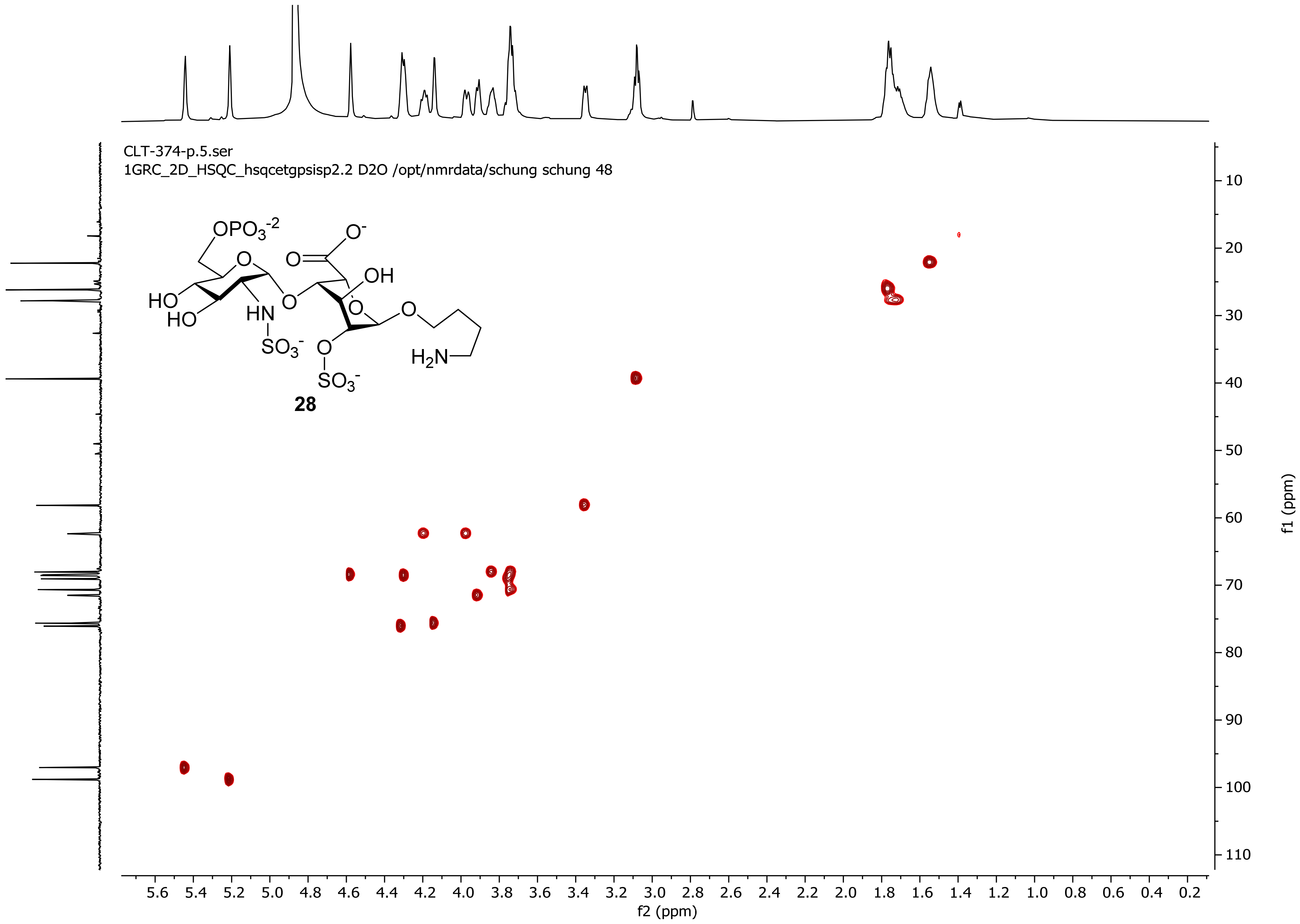
CLT-374-p.5.ser
1GRC_2D_HSQC_hsqcetgpsisp2.2 D2O /opt/nmrdata/schung schung 48
28
f2 (ppm)
f1 (ppm)

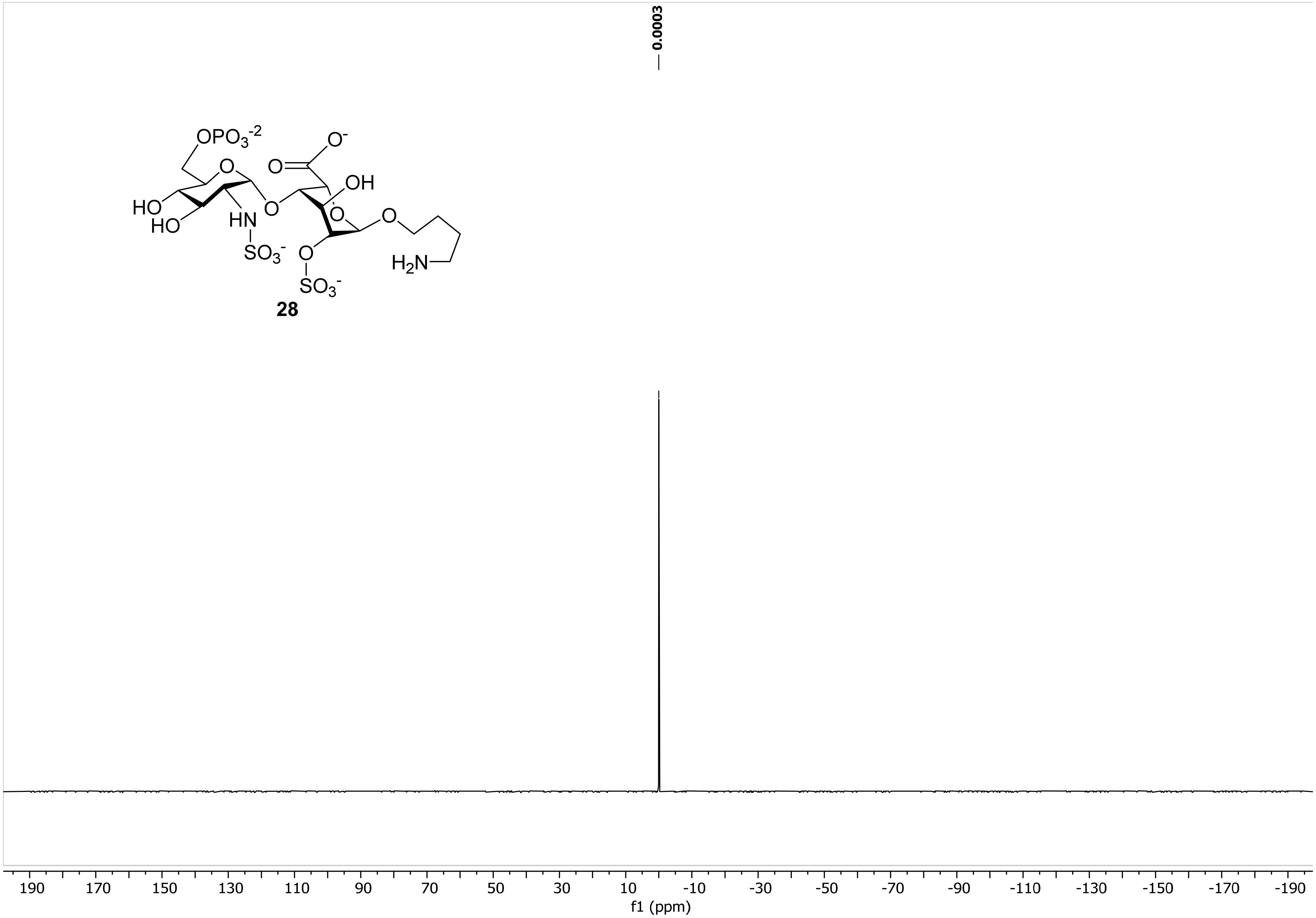

0.0003
OPO3-2
O-
OH
HO
HO
HN
SO3-
O
H2N
28
190 170 150 130 110 90 70 50 30 10 -10 -30 -50 -70 -90 -110 -130 -150 -170 -190
f1 (ppm)

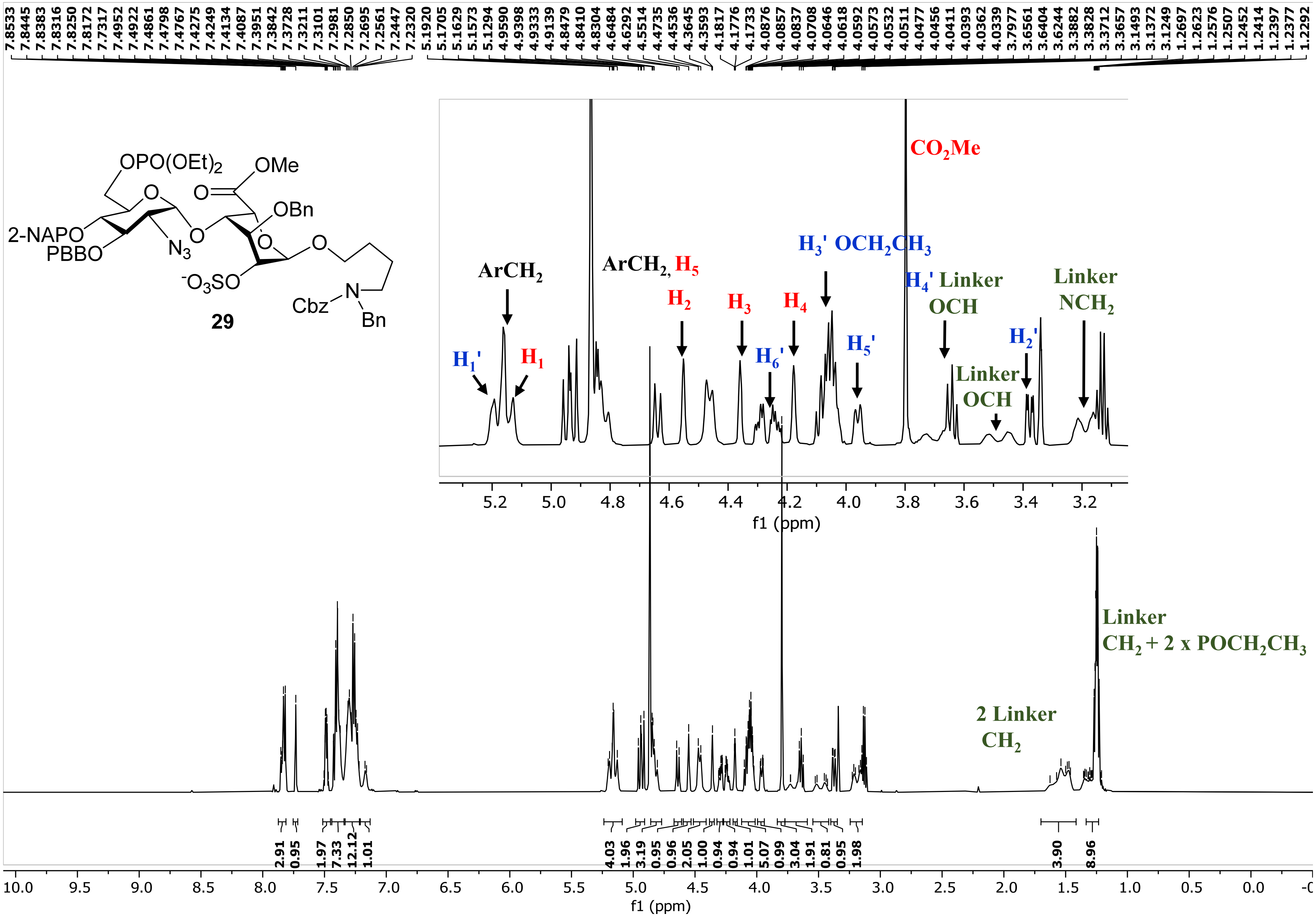
7.8533
7.8445
7.8383
7.8316
7.8250
7.8172
7.7317
7.4952
7.4922
7.4861
7.4798
7.4767
7.4275
7.4249
7.4134
7.4087
7.3951
7.3842
7.3728
7.3211
7.3101
7.2981
7.2850
7.2695
7.2561
7.2447
7.2320
5.1920
5.1705
5.1629
5.1573
5.1294
4.9590
4.9398
4.9333
4.9139
4.8479
4.8410
4.8304
4.6484
4.6292
4.5514
4.4735
4.4536
4.3645
4.3593
4.1817
4.1776
4.1733
4.0876
4.0857
4.0837
4.0708
4.0646
4.0618
4.0592
4.0573
4.0532
4.0511
4.0477
4.0456
4.0411
4.0393
4.0362
4.0339
3.7977
3.6561
3.6404
3.6244
3.3882
3.3828
3.3712
3.3657
3.1493
3.1372
3.1249
1.2697
1.2623
1.2576
1.2507
1.2452
1.2414
1.2397
1.2377
1.2292
OPO(OEt)2
OMe
O
O
OBn
2-NAPO
PBBO
N3
O
O
O
-O3SO
N
Cbz
Bn
29
CO2Me
H3' OCH2CH3
ArCH2
ArCH2, H5
H4' Linker
OCH
Linker
NCH2
H2
H3
H4
H1'
H1
H6'
H5'
H2'
Linker
OCH
f1 (ppm)
Linker
CH2 + 2 x POCH2CH3
2 Linker
CH2
2.91
0.95
1.97
7.33
12.12
1.01
4.03
1.96
3.19
0.95
0.96
2.05
1.00
0.94
0.94
1.01
5.07
0.99
3.04
1.91
0.81
0.95
1.98
3.90
8.96
f1 (ppm)

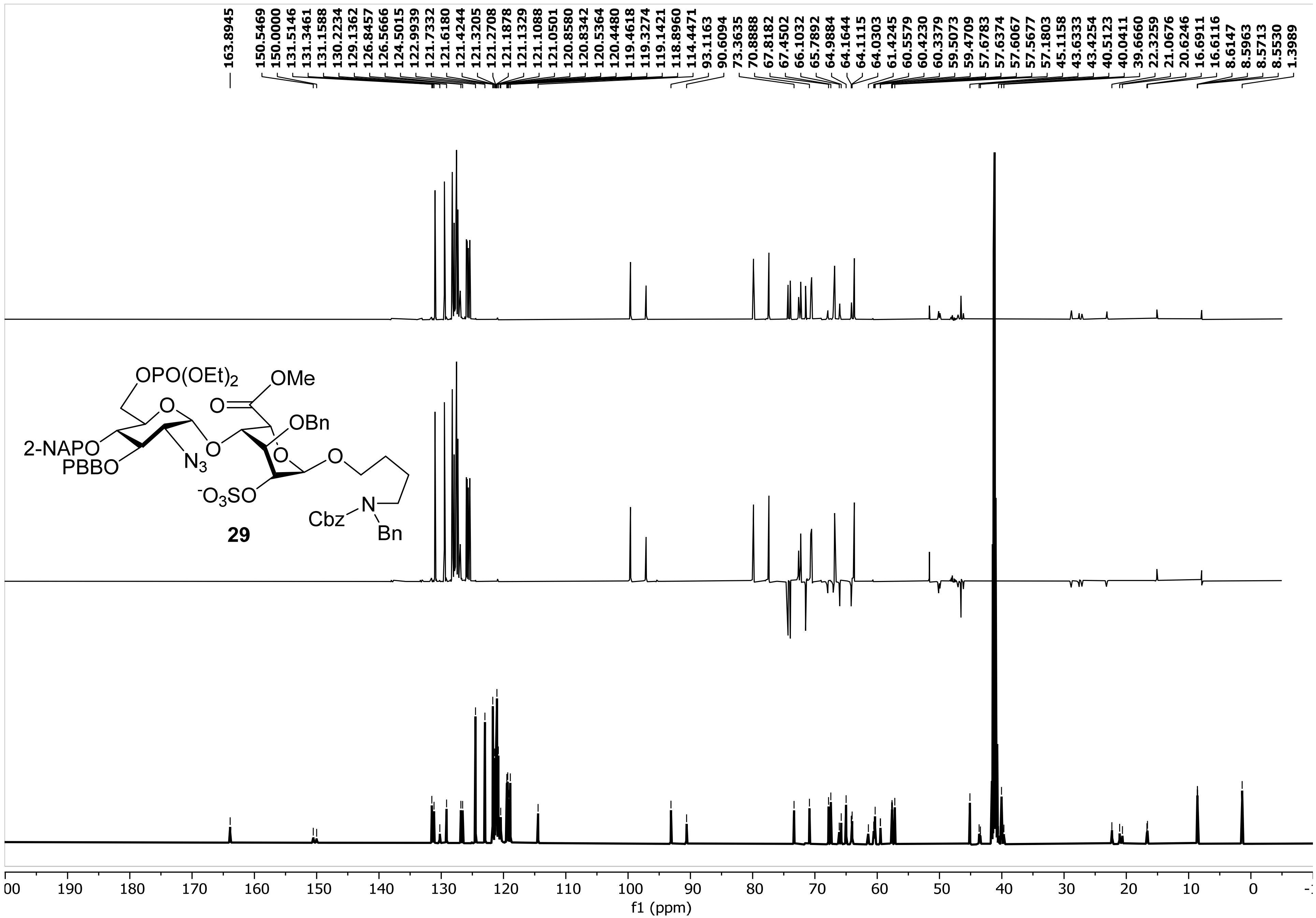
163.8945
150.5469
150.0000
131.5146
131.3461
131.1588
130.2234
129.1362
126.8457
126.5666
124.5015
122.9939
121.7332
121.6180
121.4244
121.3205
121.2708
121.1878
121.1329
121.1088
121.0501
120.8580
120.8342
120.5364
120.4480
119.4618
119.3274
119.1421
118.8960
114.4471
93.1163
90.6094
73.3635
70.8888
67.8182
67.4502
66.1032
65.7892
64.9884
64.1644
64.1115
64.0303
61.4245
60.5579
60.4230
60.3379
59.5073
59.4709
57.6783
57.6374
57.6067
57.5677
57.1803
45.1158
43.6333
43.4254
40.5123
40.0411
39.6660
22.3259
21.0676
20.6246
16.6911
16.6116
8.6147
8.5963
8.5713
8.5530
1.3989
OPO(OEt)2
OMe
O
OBn
2-NAPO
PBBO
N3
-O3SO
O
Cbz
N
Bn
29
190
180
170
160
150
140
130
120
110
100
90
80
70
60
50
40
30
20
10
0
f1 (ppm)

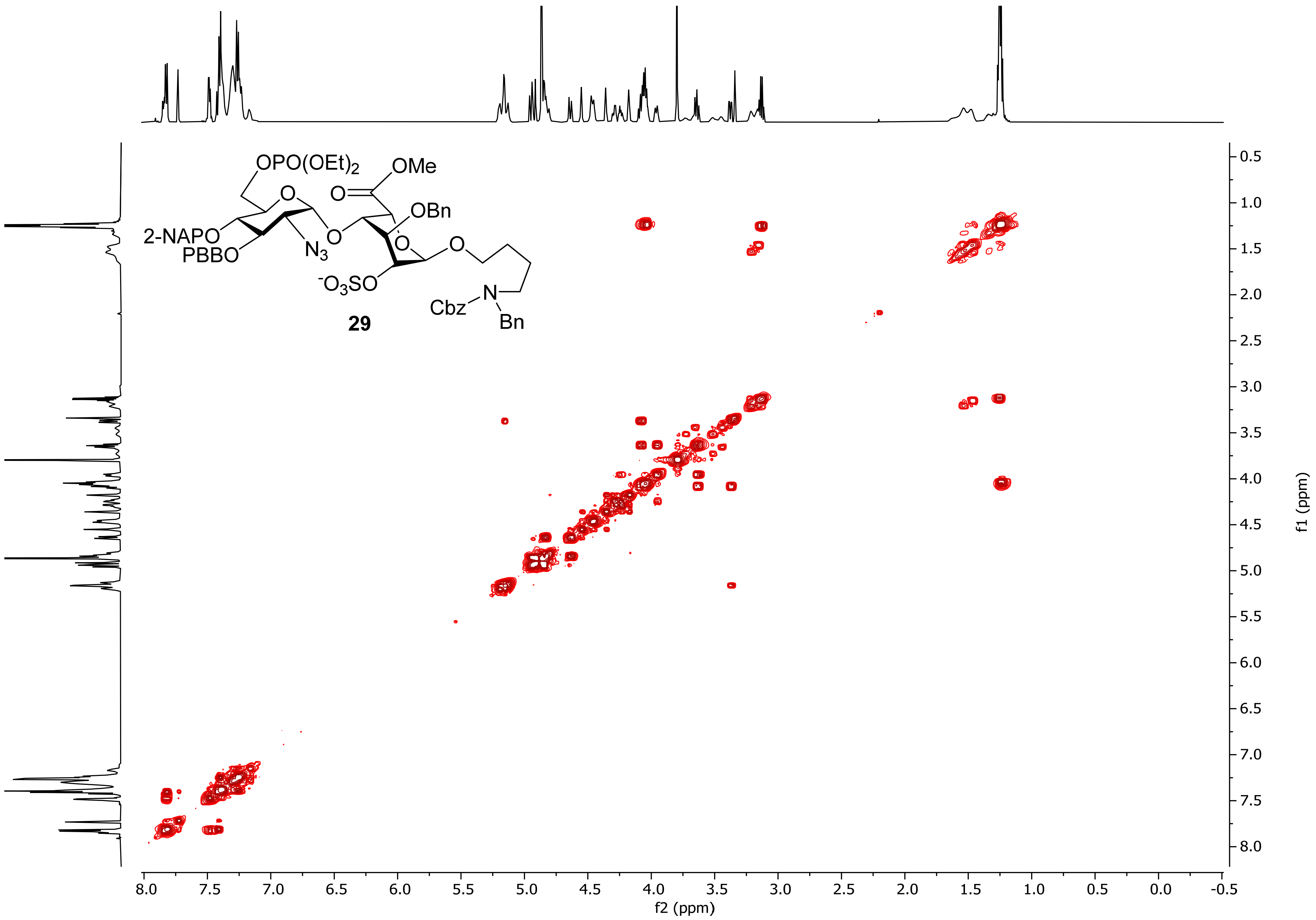
OPO(OEt)2
OMe
O
O
OBn
2-NAPO
PBBO
O
N3
O
O
-O3SO
Cbz
N
Bn
29
8.0
7.5
7.0
6.5
6.0
5.5
5.0
4.5
4.0
3.5
3.0
2.5
2.0
1.5
1.0
0.5
0.0
-0.5
f2 (ppm)
f1 (ppm)

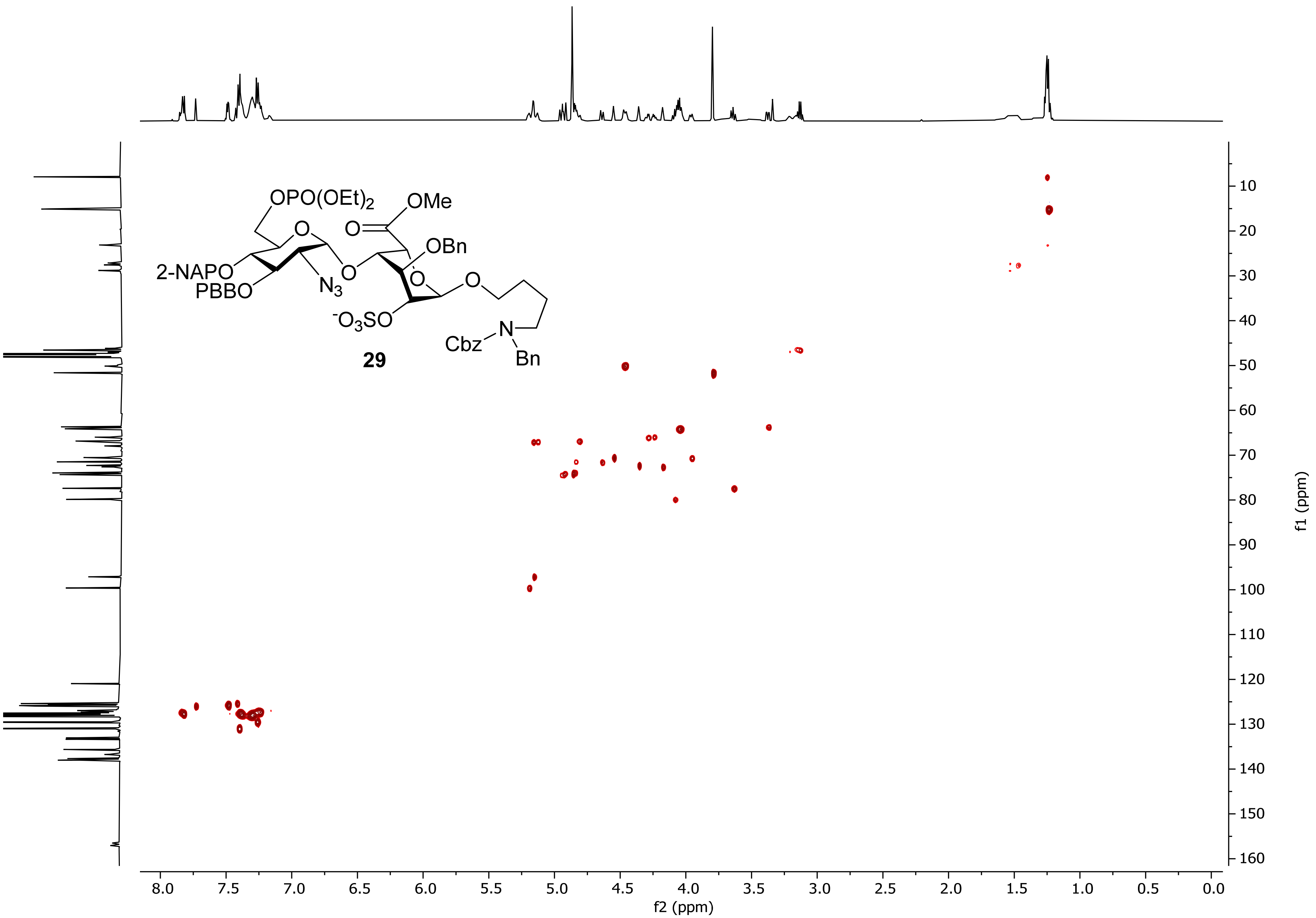

OPO(OEt)2
OMe
O
O
OBn
2-NAPO
PBBO
N3
O
O
O
-O3SO
Cbz
N
Bn
29
f2 (ppm)
f1 (ppm)

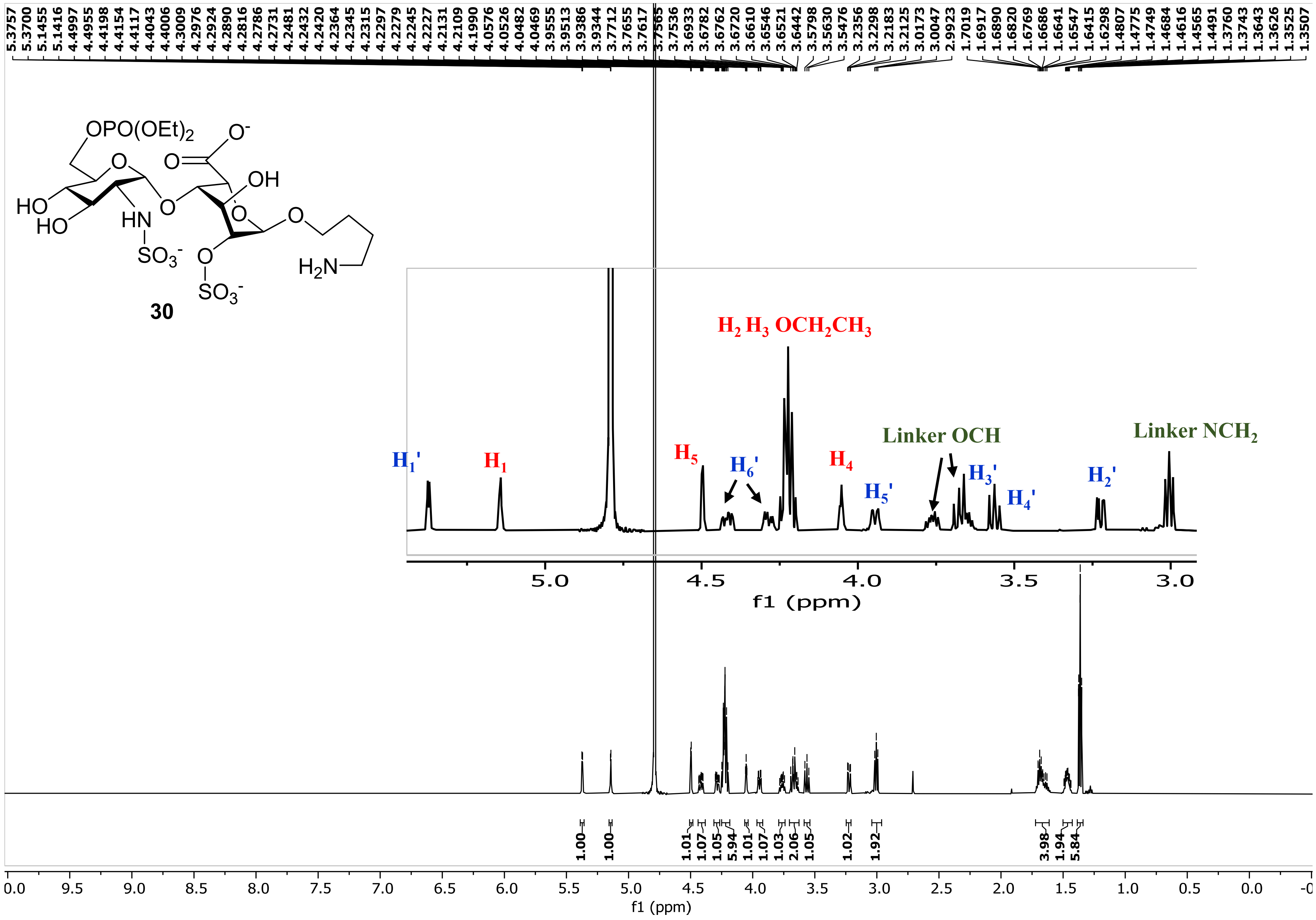

OPO(OEt)2
30
H1'
H1
H5
H6'
H2 H3 OCH2CH3
H4
H5'
Linker OCH
H3'
H4'
H2'
Linker NCH2
f1 (ppm)

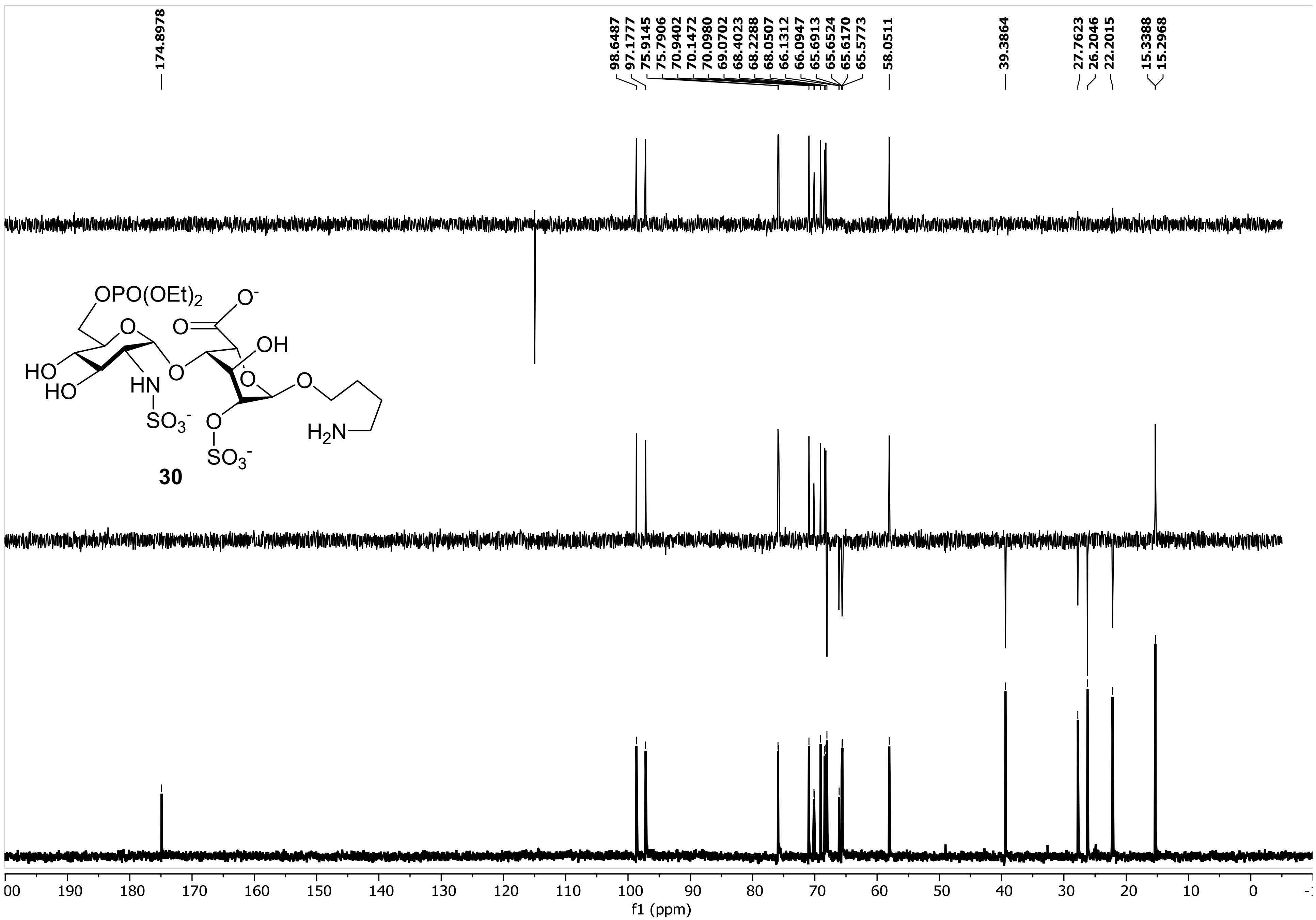
174.8978
98.6487
97.1777
75.9145
75.7906
70.9402
70.1472
70.0980
69.0702
68.4023
68.2288
68.0507
66.1312
66.0947
65.6913
65.6524
65.6170
65.5773
58.0511
39.3864
27.7623
26.2046
22.2015
15.3388
15.2968
OPO(OEt)2
O-
O
O
OH
HO
HO
HN
O
O
O
SO3-
O
SO3-
H2N
30
f1 (ppm)

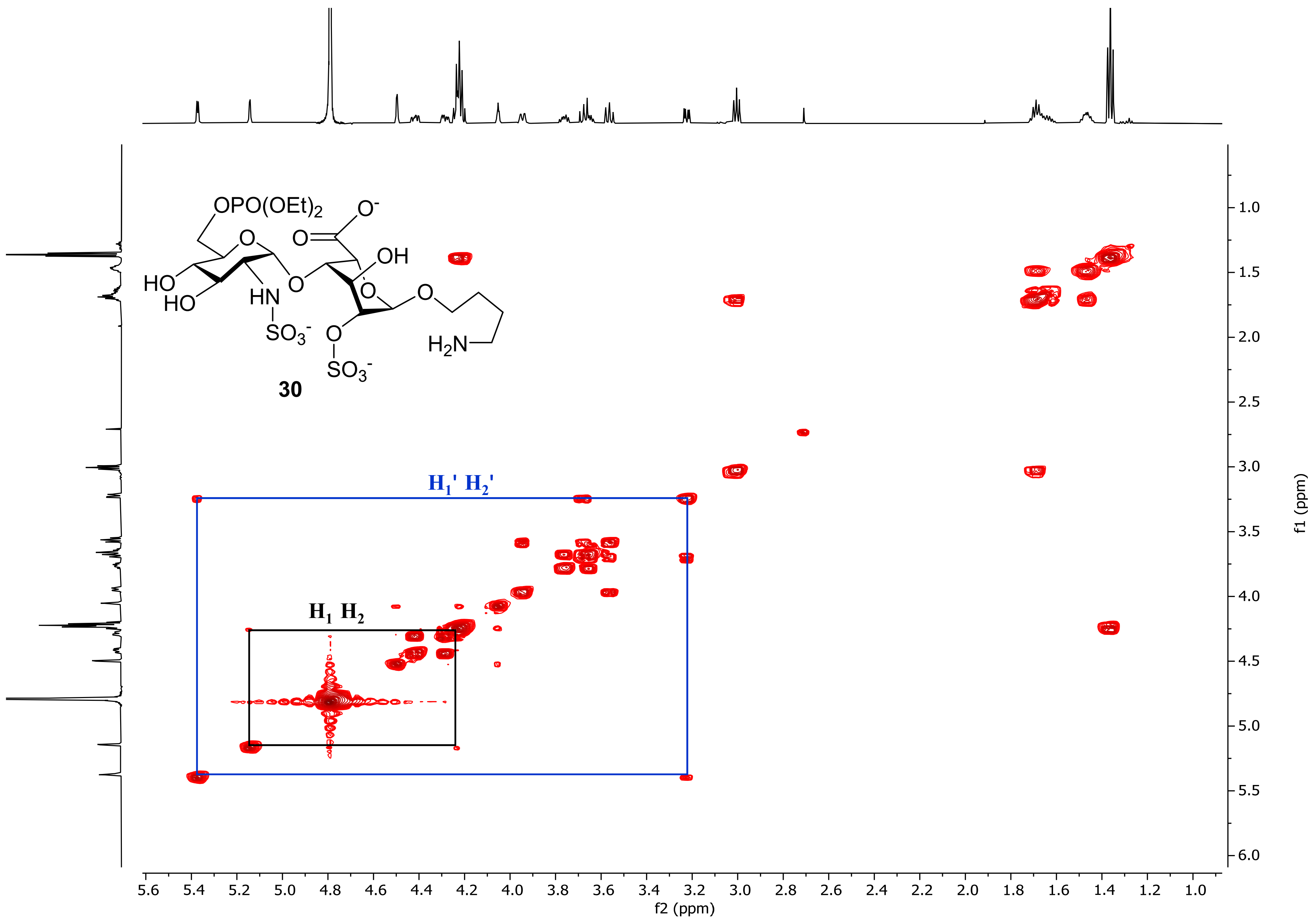
OPO(OEt)2
O-
O
O
OH
HO
HO
HN
O
O
O
SO3-
O
H2N
SO3-
30
H1' H2'
H1 H2
f2 (ppm)
f1 (ppm)

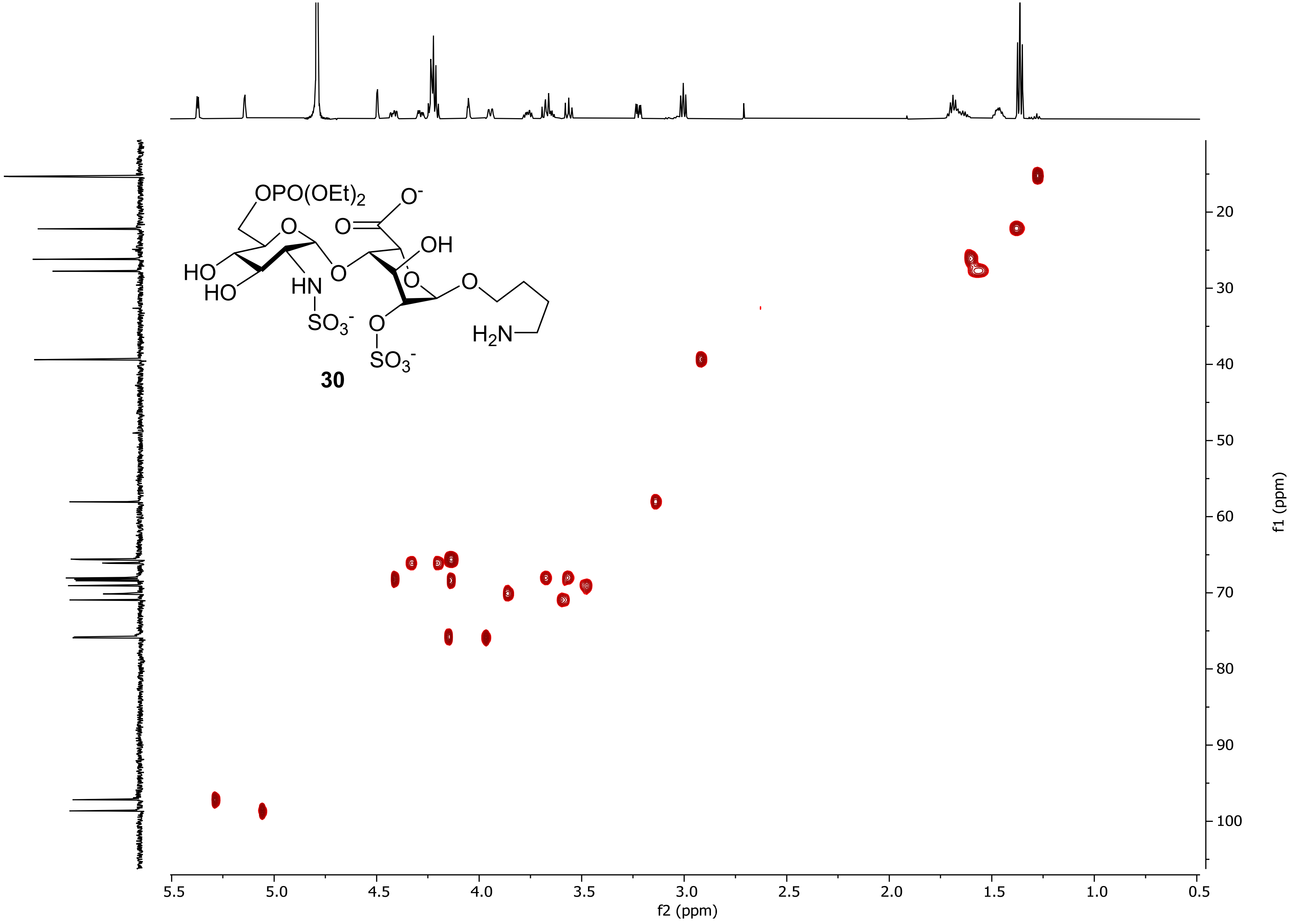
OPO(OEt)2
O-
O
O
OH
HO
HO
HN
O
O
O
SO3-
O
H2N
SO3-
30
20
30
40
50
60
70
80
90
100
f1 (ppm)
5.5
5.0
4.5
4.0
3.5
3.0
2.5
2.0
1.5
1.0
0.5
f2 (ppm)

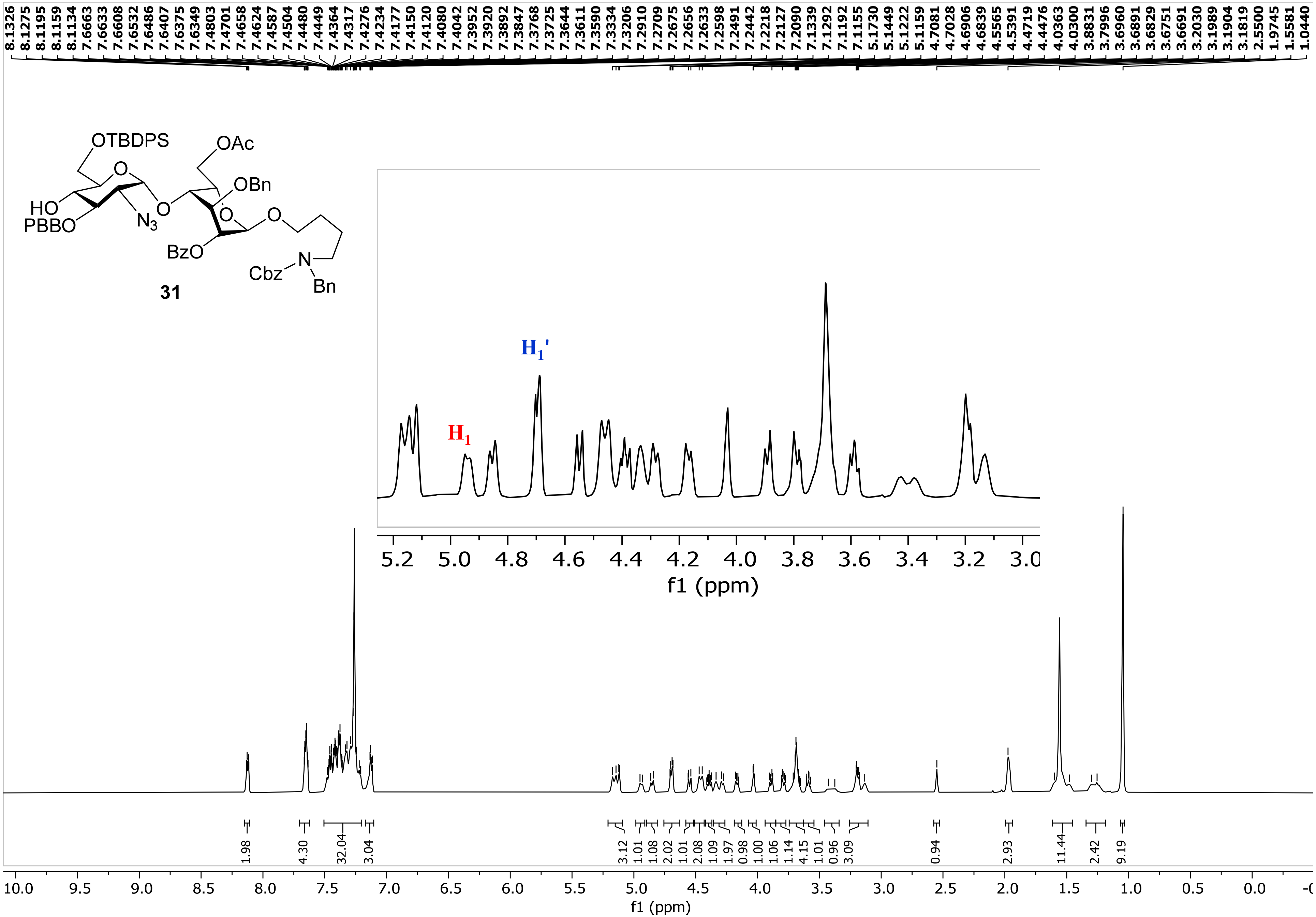
8.1326
8.1275
8.1195
8.1159
8.1134
7.6663
7.6633
7.6608
7.6532
7.6486
7.6407
7.6375
7.6349
7.4803
7.4701
7.4658
7.4624
7.4587
7.4504
7.4480
7.4449
7.4364
7.4317
7.4276
7.4234
7.4177
7.4150
7.4120
7.4080
7.4042
7.3952
7.3920
7.3892
7.3847
7.3768
7.3725
7.3644
7.3611
7.3590
7.3334
7.3206
7.2910
7.2709
7.2675
7.2656
7.2633
7.2598
7.2491
7.2442
7.2218
7.2127
7.2090
7.1339
7.1292
7.1192
7.1155
5.1730
5.1449
5.1222
5.1159
4.7081
4.7028
4.6906
4.6839
4.5565
4.5391
4.4719
4.4476
4.0363
4.0300
3.8831
3.7996
3.6960
3.6891
3.6829
3.6751
3.6691
3.2030
3.1989
3.1904
3.1819
2.5500
1.9745
1.5581
1.0440
OTBDPS
OAc
OBn
HO
PBBO
N3
O
BzO
Cbz
N
Bn
31
H1
H1'
5.2
5.0
4.8
4.6
4.4
4.2
4.0
3.8
3.6
3.4
3.2
3.0
f1 (ppm)
1.98
4.30
32.04
3.04
3.12
1.01
1.08
2.02
1.01
2.08
1.09
1.97
0.98
1.00
1.06
1.14
4.15
1.01
0.96
3.09
0.94
2.93
11.44
2.42
9.19
10.0
9.5
9.0
8.5
8.0
7.5
7.0
6.5
6.0
5.5
5.0
4.5
4.0
3.5
3.0
2.5
2.0
1.5
1.0
0.5
0.0
f1 (ppm)

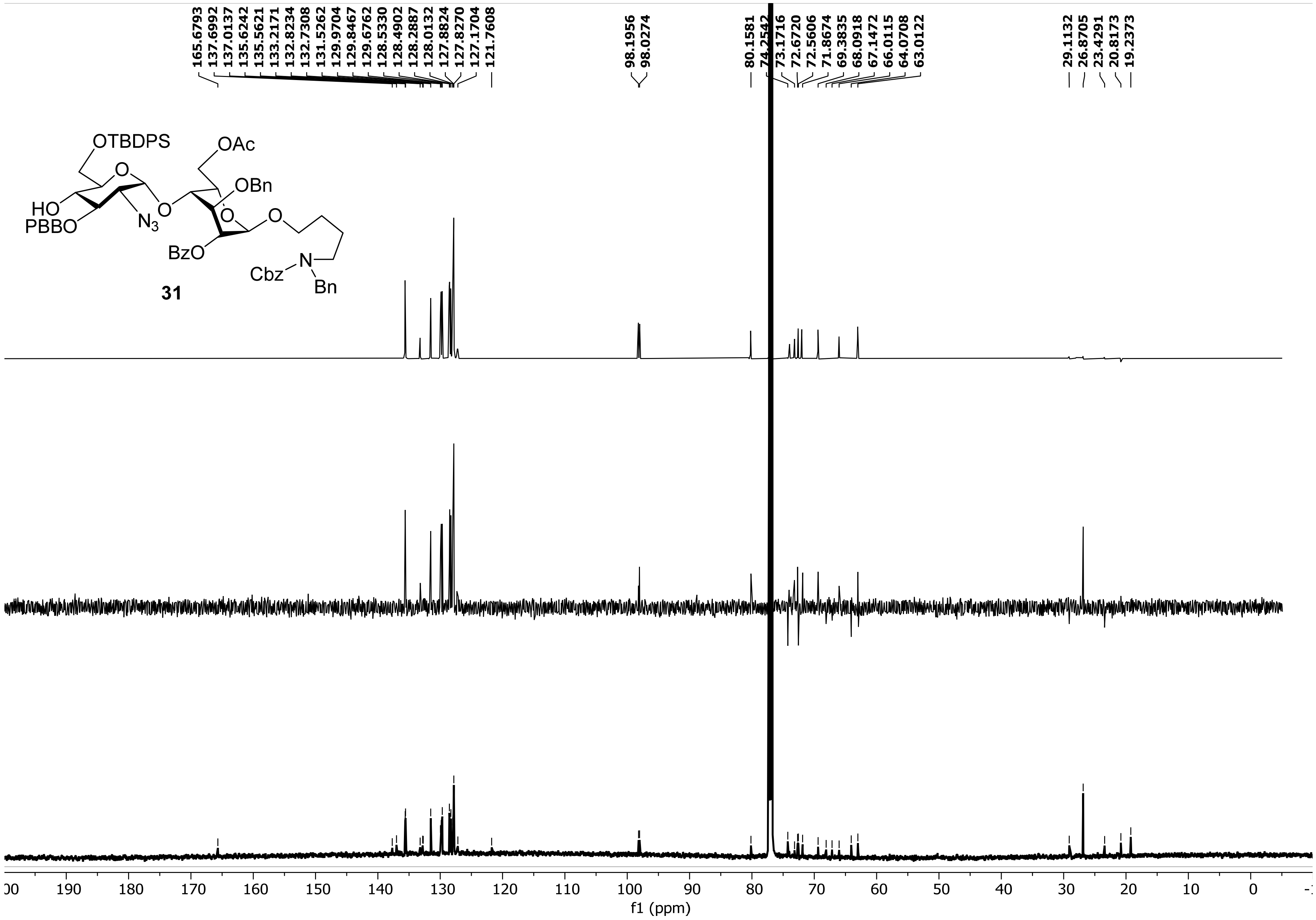

165.6793
137.6992
137.0137
135.6242
135.5621
133.2171
132.8234
132.7308
131.5262
129.9704
129.8467
129.6762
128.5330
128.4902
128.2887
128.0132
127.8824
127.8270
127.1704
121.7608
98.1956
98.0274
80.1581
74.2542
73.1716
72.6720
72.5606
71.8674
69.3835
68.0918
67.1472
66.0115
64.0708
63.0122
29.1132
26.8705
23.4291
20.8173
19.2373
OTBDPS
OAc
OBn
HO
PBBO
N3
BzO
Cbz
N
Bn
31
190
180
170
160
150
140
130
120
110
100
90
80
70
60
50
40
30
20
10
0
f1 (ppm)

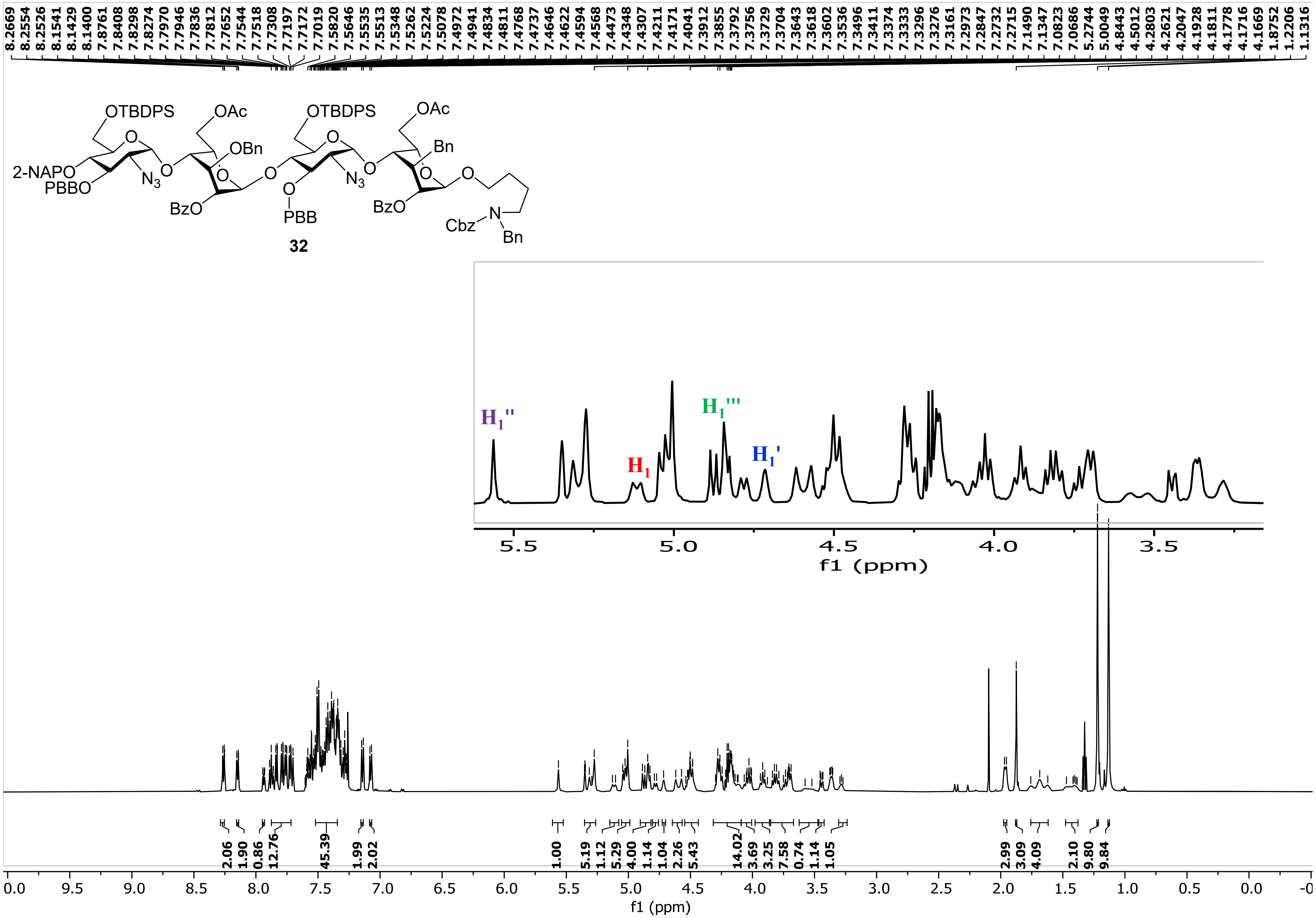
OTBDPS
OAc
OBn
OTBDPS
OAc
Bn
2-NAPO
PBBO
BzO
PBB
BzO
Cbz
N
Bn
32
H1''
H1
H1'''
H1'
f1 (ppm)
f1 (ppm)

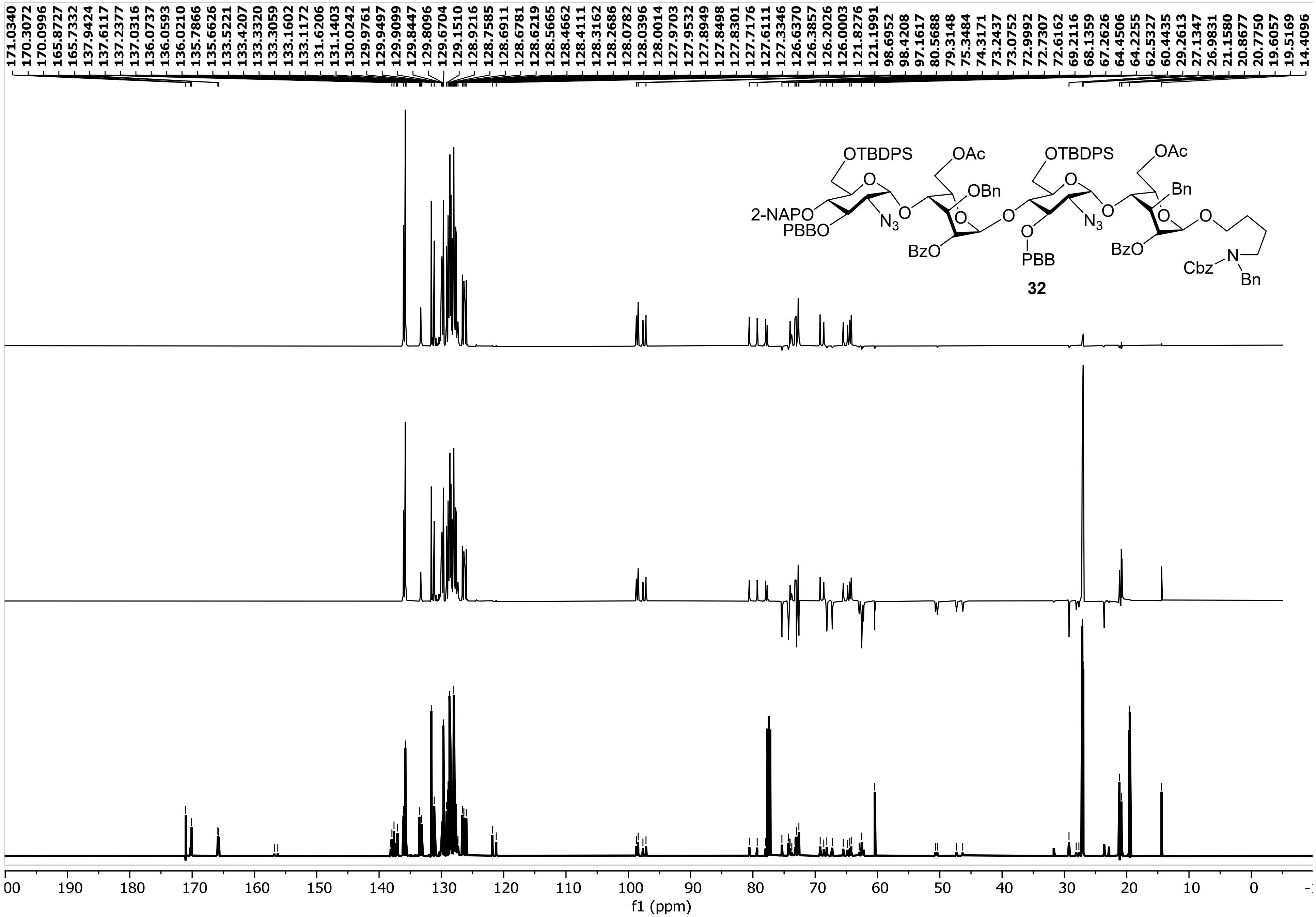
171.0340
170.3072
170.0996
165.8727
165.7332
137.9424
137.6117
137.2377
137.0316
136.0737
136.0593
136.0210
135.7866
135.6626
133.5221
133.4207
133.3320
133.3059
133.1602
133.1172
131.6206
131.1403
130.0242
129.9761
129.9497
129.9099
129.8447
129.8096
129.6704
129.1510
128.9216
128.7585
128.6911
128.6781
128.6219
128.5665
128.4662
128.4111
128.3162
128.2686
128.0782
128.0396
128.0014
127.9703
127.9532
127.8949
127.8498
127.8301
127.7176
127.6111
127.3346
126.6370
126.3857
126.2026
126.0003
121.8276
121.1991
98.6952
98.4208
97.1617
80.5688
79.3148
75.3484
74.3171
73.2437
73.0752
72.9992
72.7307
72.6162
69.2116
68.1359
67.2626
64.4506
64.2255
62.5327
60.4435
29.2613
27.1347
26.9831
21.1580
20.8677
20.7750
19.6057
19.5169
14.4096
OTBDPS
OAc
OTBDPS
OAc
O
OBn
O
Bn
2-NAPO
O
O
O
O
O
PBBO
N3
BzO
O
PBB
N3
BzO
Cbz
N
Bn
32
f1 (ppm)

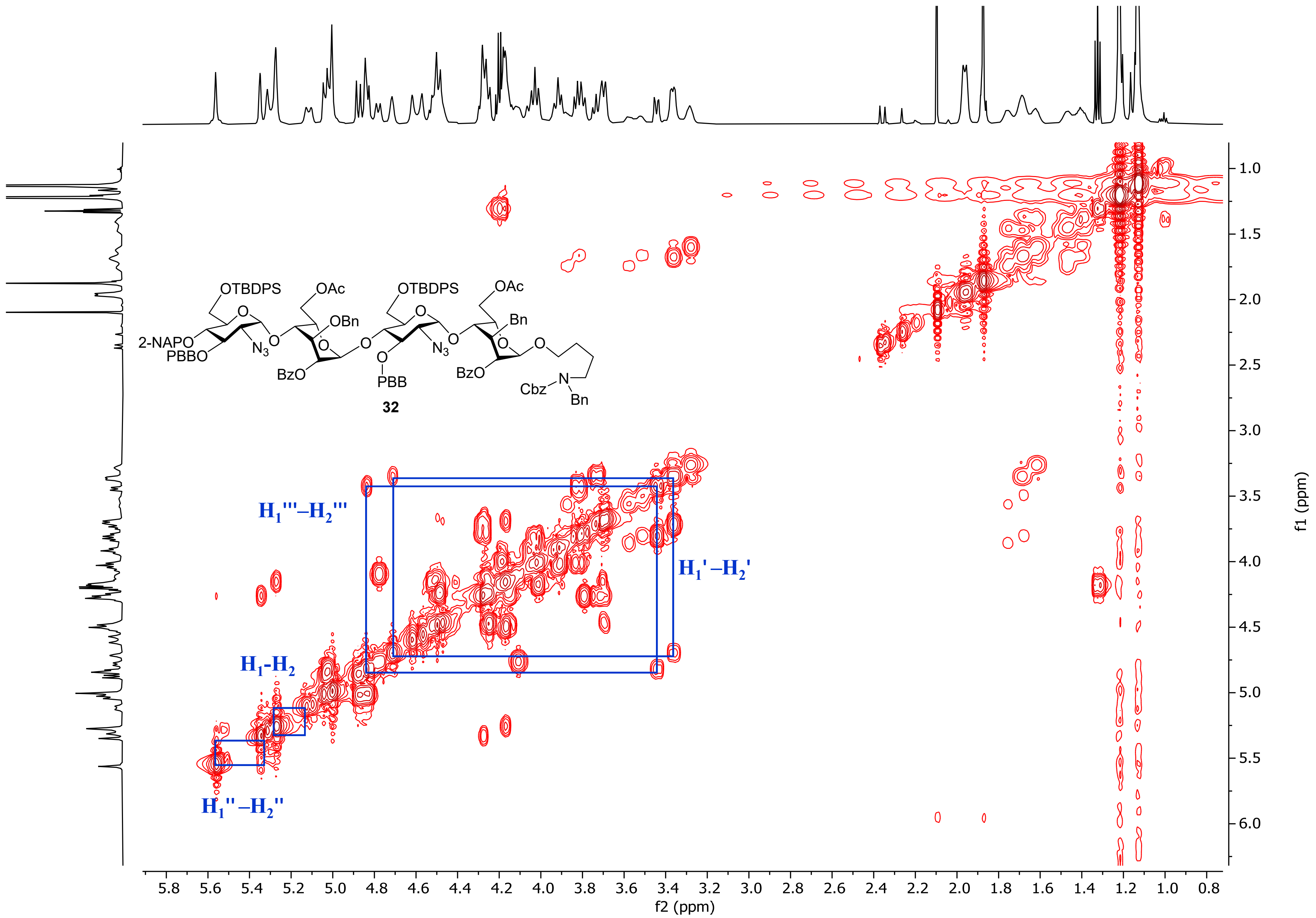

OTBDPS
OAc
OBn
OTBDPS
OAc
Bn
2-NAPO
PBBO
N_3
BzO
PBB
N_3
BzO
Cbz
N
Bn
32
H1'''–H2'''
H1'–H2'
H1-H2
H1''–H2''
f2 (ppm)
f1 (ppm)

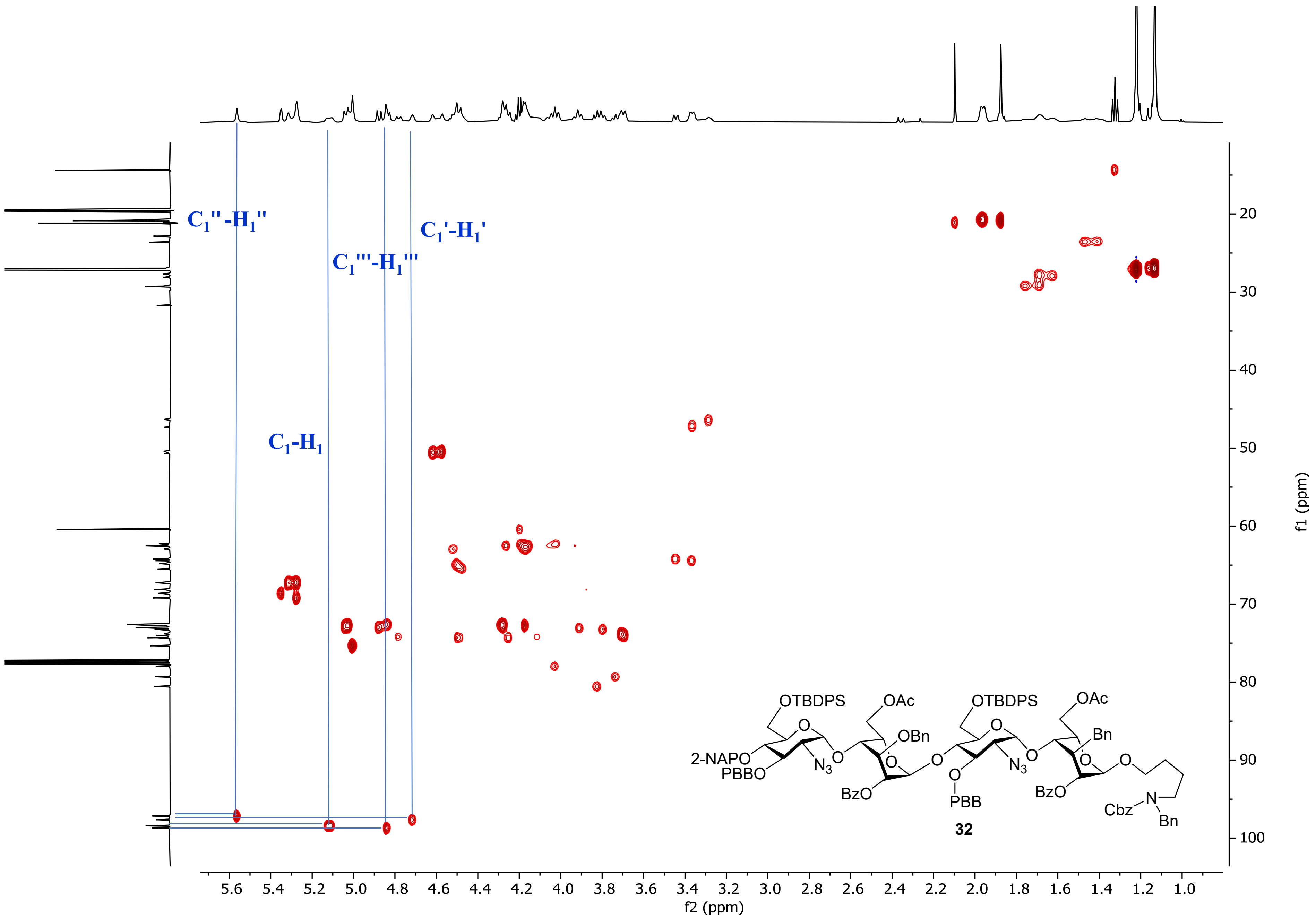
C1''-H1''
C1'''-H1'''
C1'-H1'
C1-H1
OTBDPS
OAc
OBn
2-NAPO
PBBO
N3
BzO
PBB
Bn
Cbz
32
f2 (ppm)
f1 (ppm)

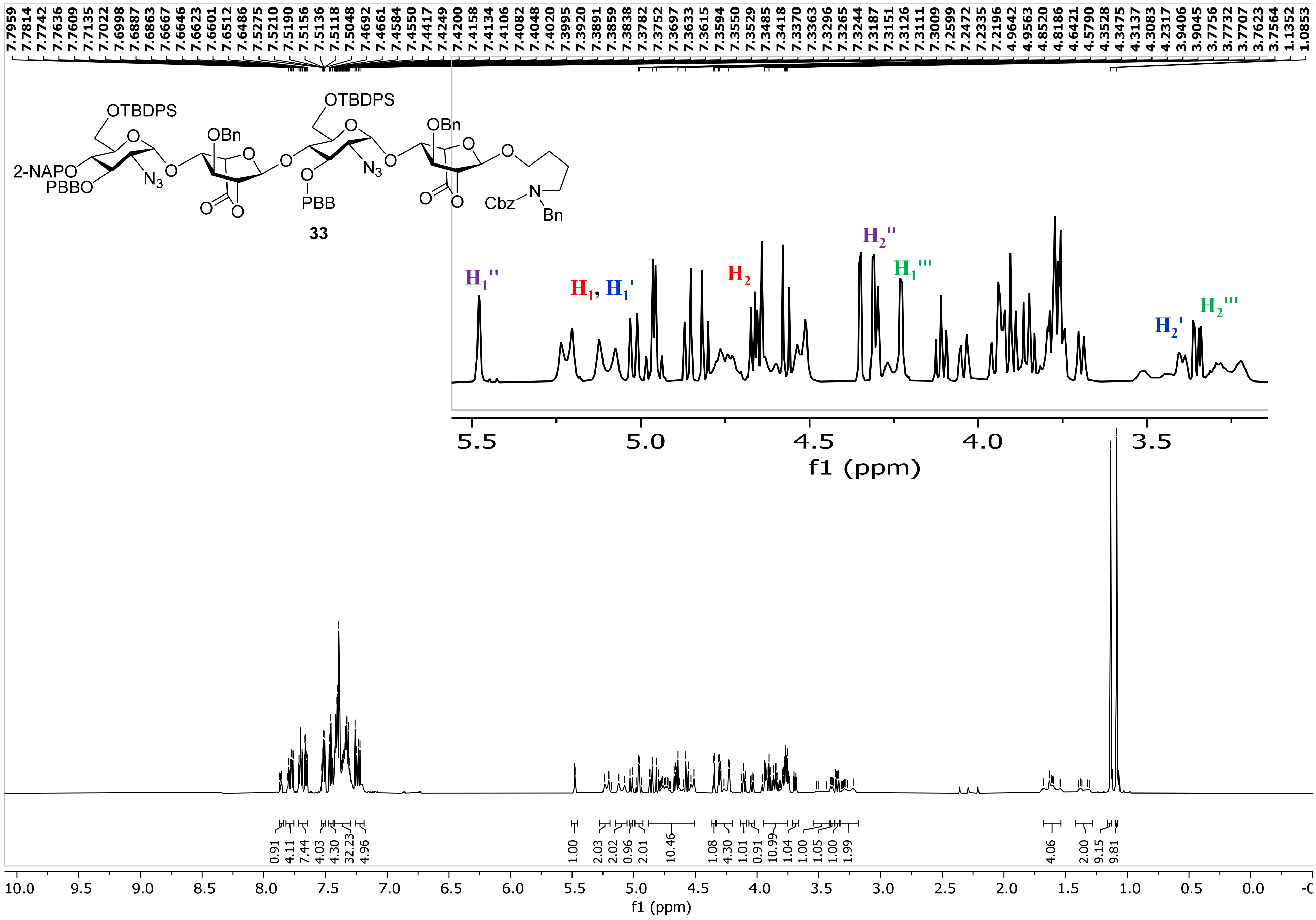
OTBDPS
2-NAPO
PBBO
N3
OBn
PBB
Cbz
Bn
33
H1''
H1, H1'
H2
H2''
H1'''
H2'
H2'''
f1 (ppm)

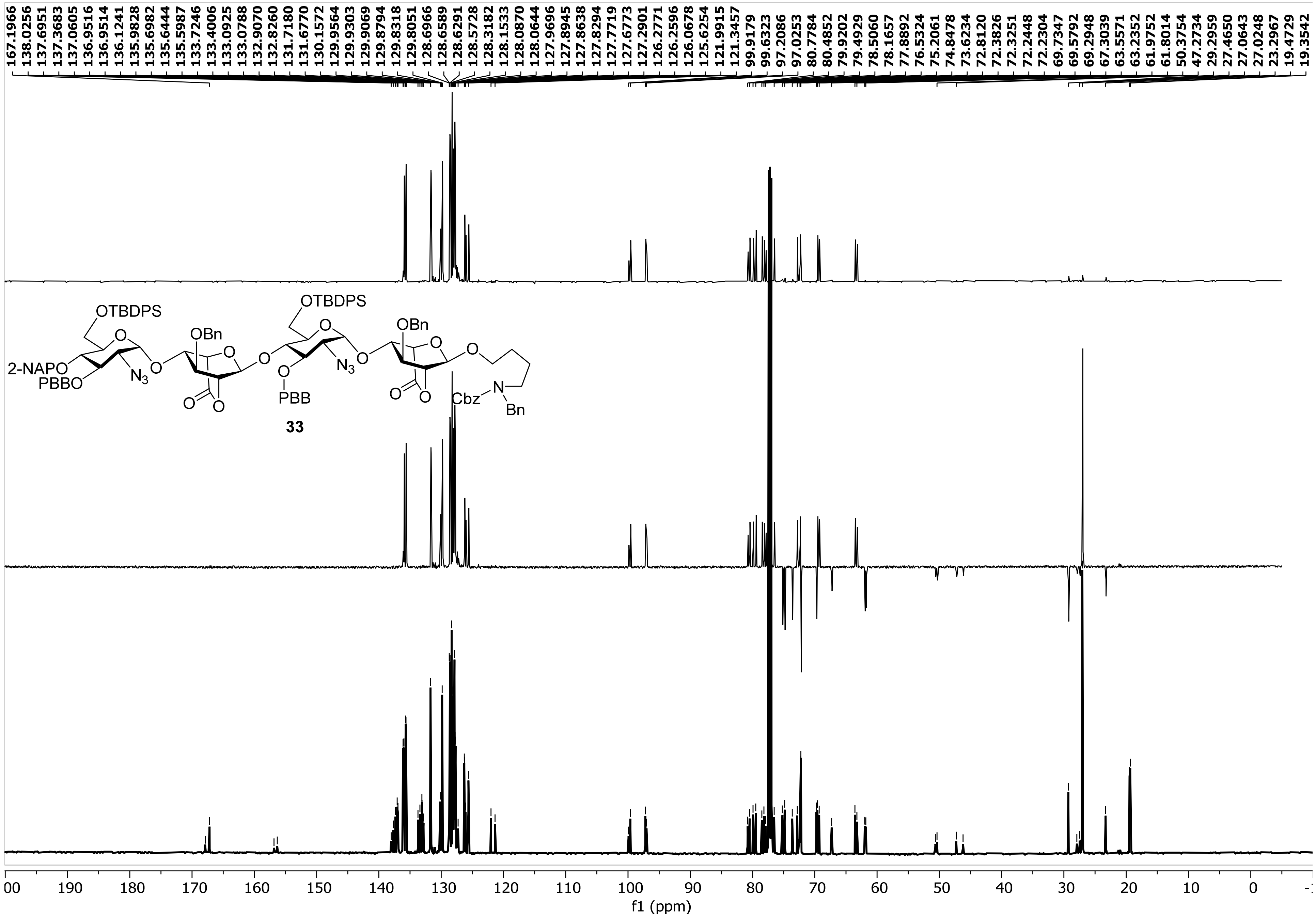
167.1966
138.0256
137.6951
137.3683
137.0605
136.9516
136.9514
136.1241
135.9828
135.6982
135.6444
135.5987
133.7246
133.4006
133.0925
133.0788
132.9070
132.8260
131.7180
131.6770
130.1572
129.9564
129.9303
129.9069
129.8794
129.8318
129.8051
128.6966
128.6589
128.6291
128.5728
128.3182
128.1533
128.0870
128.0644
127.9696
127.8945
127.8638
127.8294
127.7719
127.6773
127.2901
126.2771
126.2596
126.0678
125.6254
121.9915
121.3457
99.9179
99.6323
97.2086
97.0253
80.7784
80.4852
79.9202
79.4929
78.5060
78.1657
77.8892
76.5324
75.2061
74.8478
73.6234
72.8120
72.3826
72.3251
72.2448
72.2304
69.7347
69.5792
69.2948
67.3039
63.5571
63.2352
61.9752
61.8014
50.3754
47.2734
29.2959
27.4650
27.0643
27.0248
23.2967
19.4729
19.3542
OTBDPS
2-NAPO
PBBO
N3
OBn
O
PBB
33
Cbz
N
Bn
f1 (ppm)

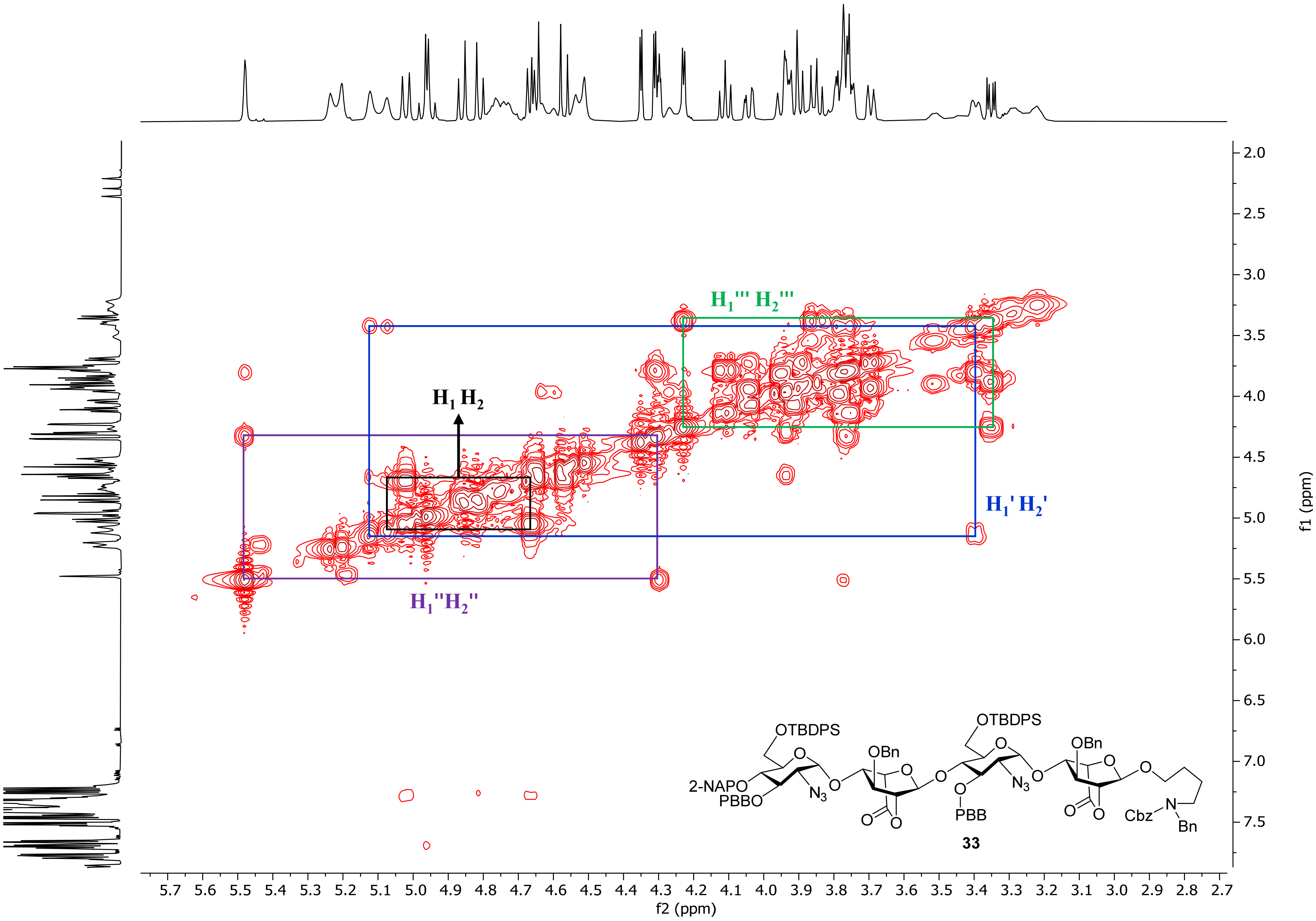
H1''' H2'''
H1 H2
H1' H2'
H1''H2''
OTBDPS
OBn
OTBDPS
OBn
2-NAPO
PBBO
N3
O
PBB
Cbz
N
Bn
33
f2 (ppm)
f1 (ppm)

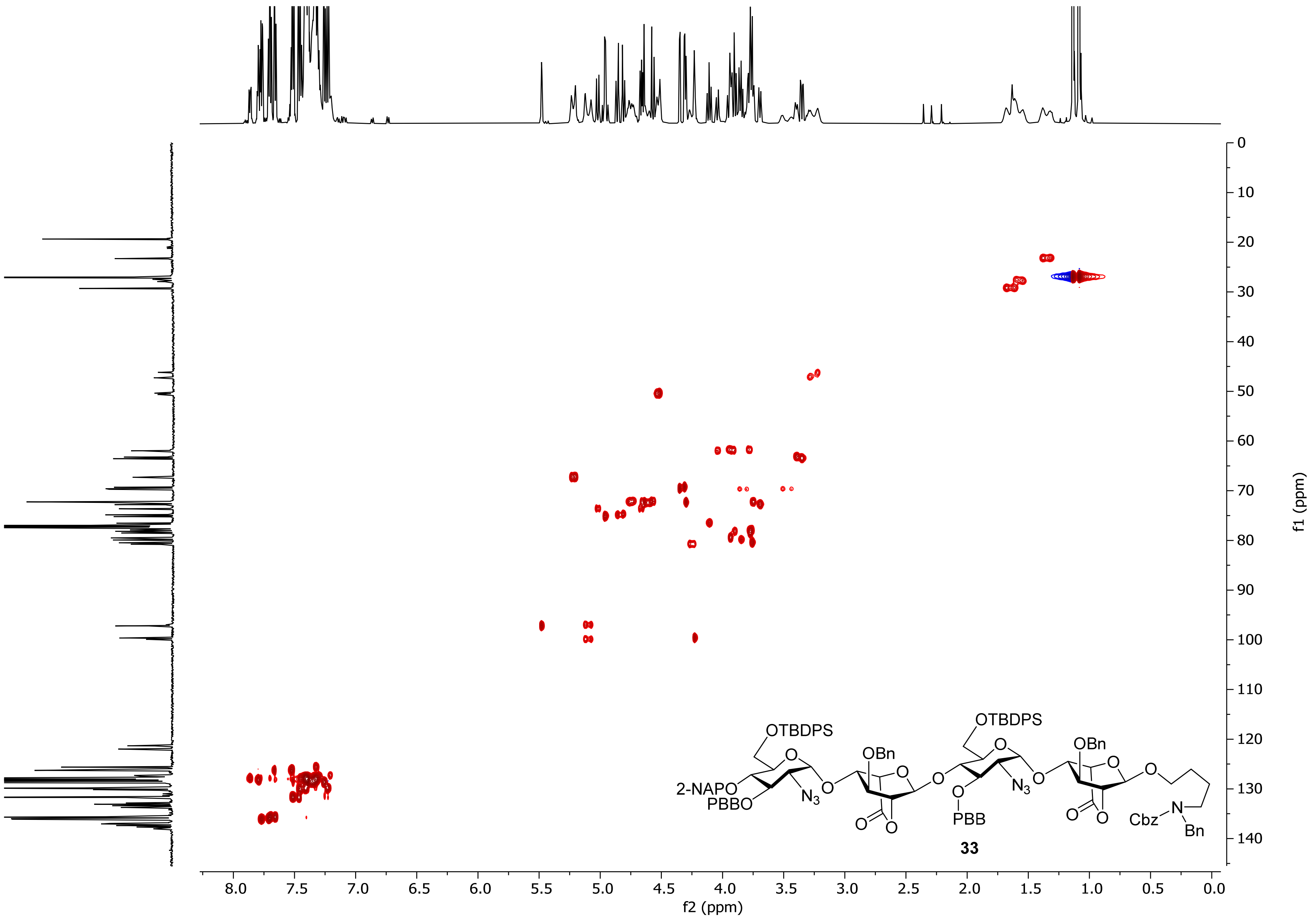

f2 (ppm)
f1 (ppm)
OTBDPS
OTBDPS
OBn
OBn
2-NAPO
PBBO
N3
N3
PBB
Cbz
N
Bn
33

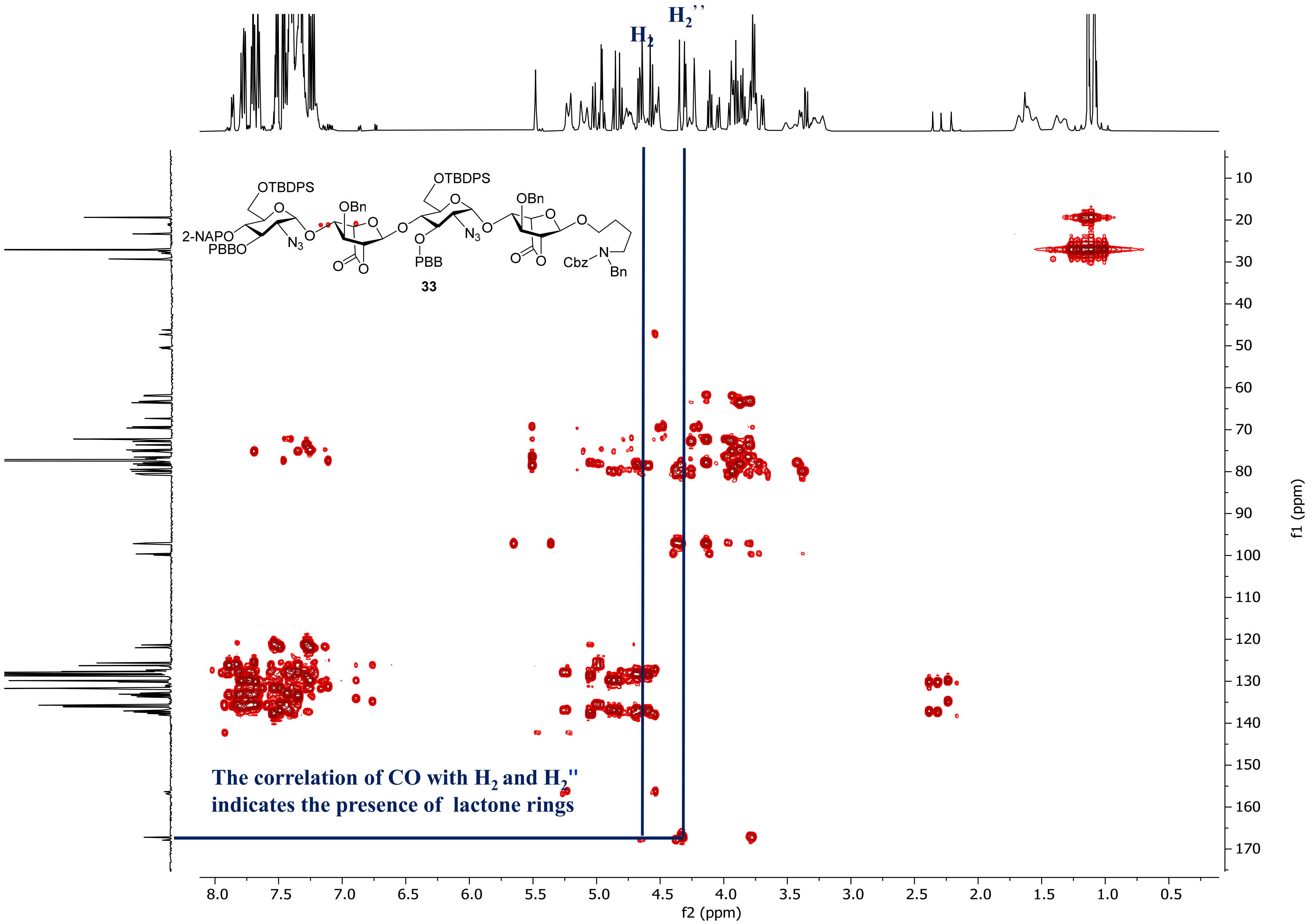

H2
H2''
OTBDPS
OBn
2-NAPO
PBBO
N3
PBB
Cbz
N
Bn
33
The correlation of CO with H2 and H2''
indicates the presence of lactone rings
f2 (ppm)
f1 (ppm)

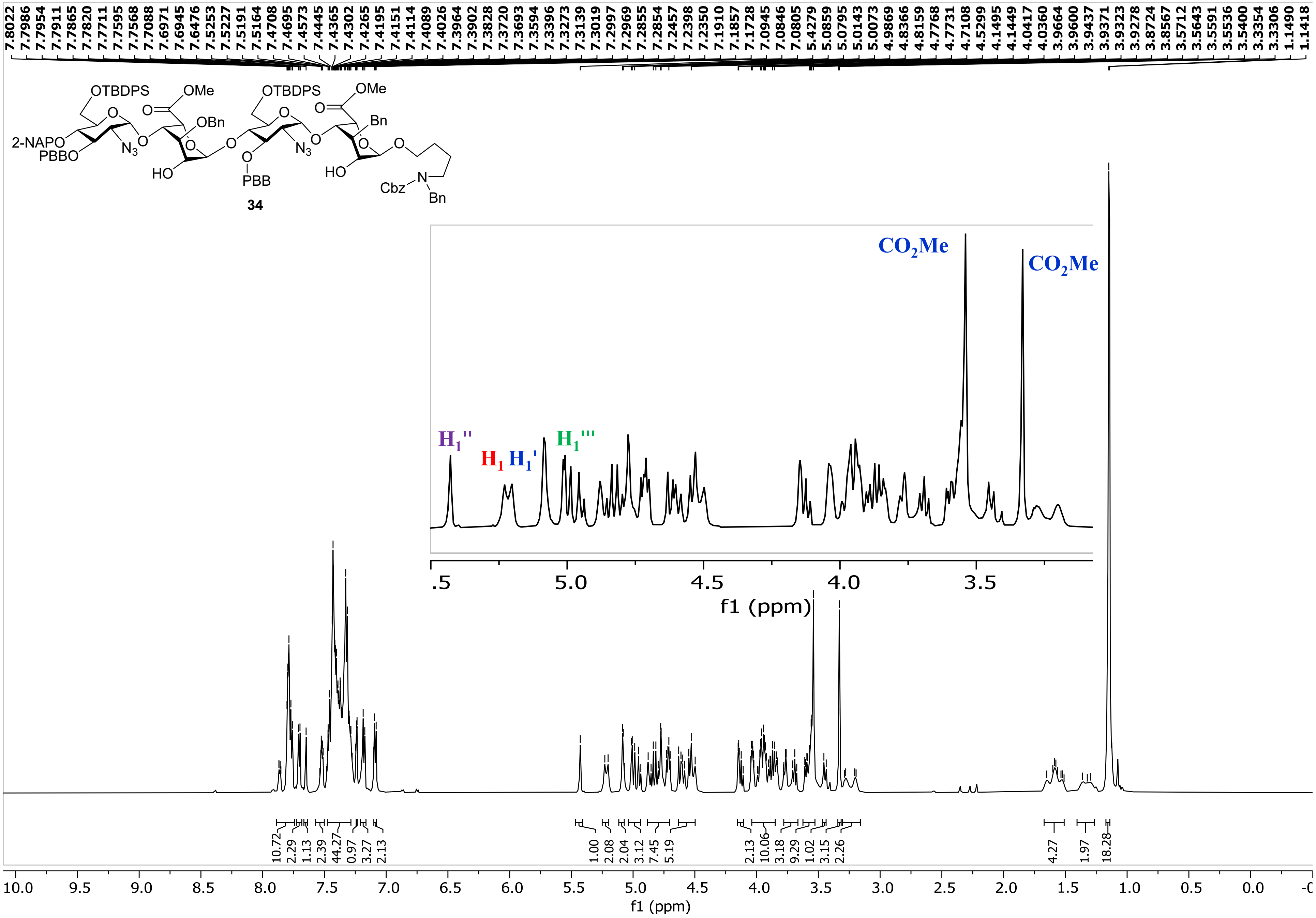
34
CO_2Me
CO_2Me
f1 (ppm)

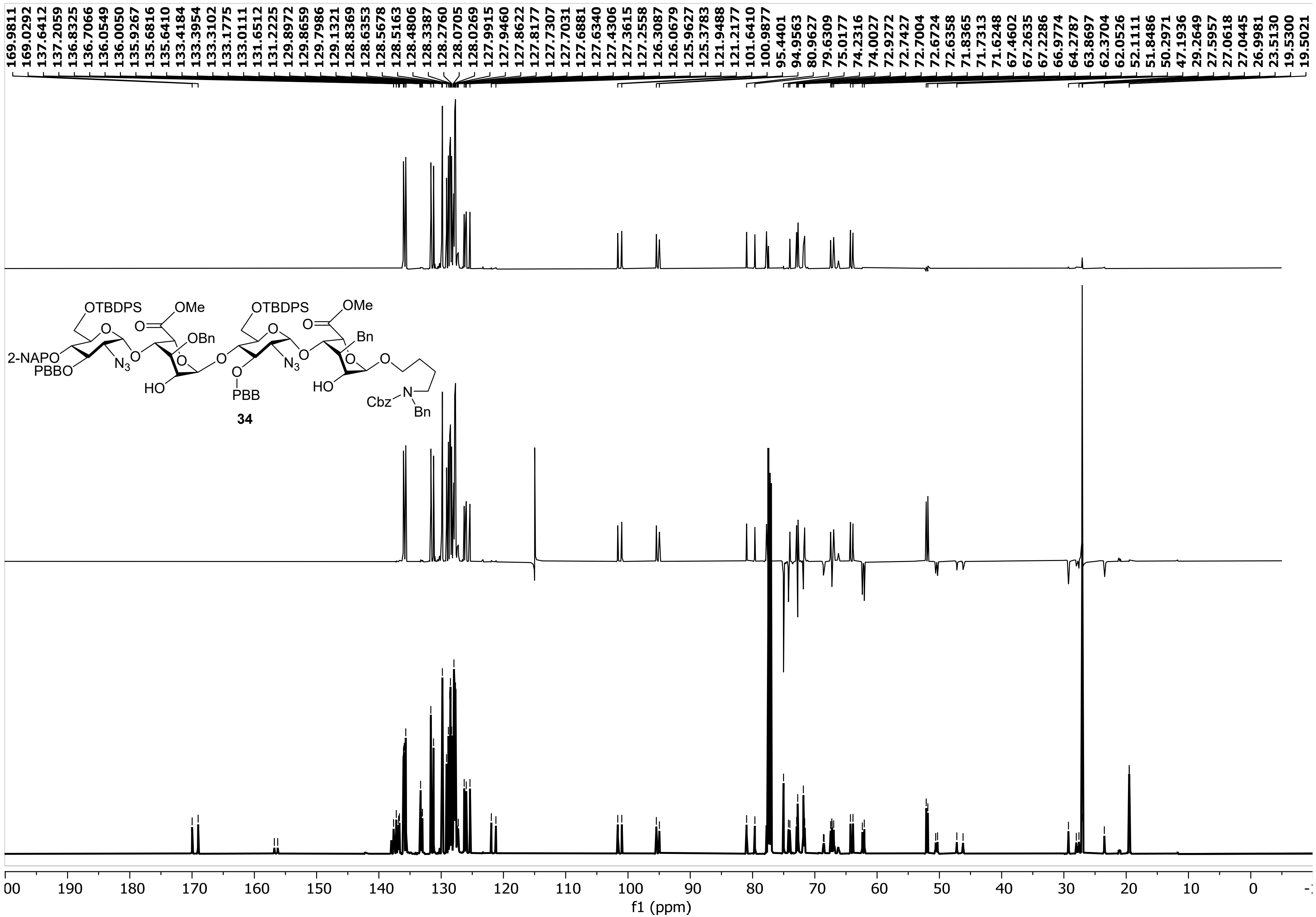
169.9811
169.0292
137.6412
137.2059
136.8325
136.7066
136.0549
136.0050
135.9267
135.6816
135.6410
133.4184
133.3954
133.3102
133.1775
133.0111
131.6512
131.2225
129.8972
129.8659
129.7986
129.1321
128.8369
128.6353
128.5678
128.5163
128.4806
128.3387
128.2760
128.0705
128.0269
127.9915
127.9460
127.8622
127.8177
127.7307
127.7031
127.6881
127.6340
127.4306
127.3615
127.2558
126.3087
126.0679
125.9627
125.3783
121.9488
121.2177
101.6410
100.9877
95.4401
94.9563
80.9627
79.6309
75.0177
74.2316
74.0027
72.9272
72.7427
72.7004
72.6724
72.6358
71.8365
71.7313
71.6248
67.4602
67.2635
67.2286
66.9774
64.2787
63.8697
62.3704
62.0526
52.1111
51.8486
50.2971
47.1936
29.2649
27.5957
27.0618
27.0445
26.9981
23.5130
19.5300
19.5021
OTBDPS
OMe
OBn
2-NAPO
PBBO
N_3
HO
O
PBB
Bn
Cbz
N
34
f1 (ppm)

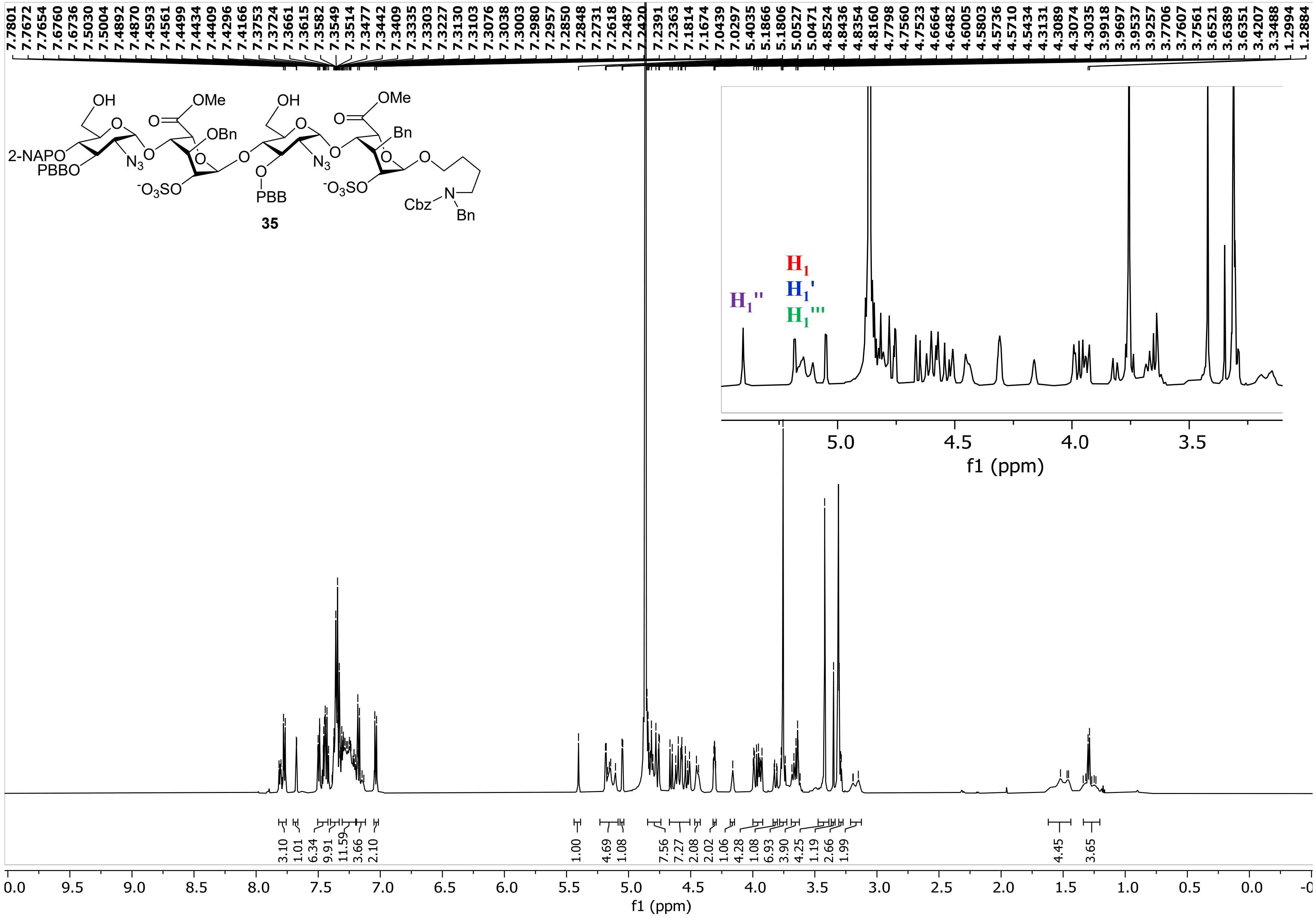
7.7801 7.7672 7.7654 7.6760 7.6736 7.5030 7.5004 7.4892 7.4870 7.4593 7.4561 7.4499 7.4434 7.4409 7.4296 7.4166 7.3753 7.3724 7.3661 7.3615 7.3582 7.3549 7.3514 7.3477 7.3442 7.3409 7.3335 7.3303 7.3227 7.3130 7.3103 7.3076 7.3038 7.3003 7.2980 7.2957 7.2850 7.2848 7.2731 7.2618 7.2487 7.2420 7.2391 7.2363 7.1814 7.1674 7.0439 7.0297 5.4035 5.1866 5.1806 5.0527 5.0471 4.8524 4.8436 4.8354 4.8160 4.7798 4.7560 4.7523 4.6664 4.6482 4.6005 4.5803 4.5736 4.5710 4.5434 4.3131 4.3089 4.3074 4.3035 3.9918 3.9697 3.9537 3.9257 3.7706 3.7607 3.7561 3.6521 3.6389 3.6351 3.4207 3.3488 1.2994 1.2884
OH
OMe
OBn
Bn
2-NAPO
PBBO
N3
-O3SO
PBB
Cbz
N
35
H1''
H1
H1'
H1'''
f1 (ppm)
3.10 1.01 6.34 9.91 11.59 3.66 2.10 1.00 4.69 1.08 7.56 7.27 2.08 2.02 1.06 4.28 1.08 6.93 3.90 4.25 1.19 2.66 1.99 4.45 3.65

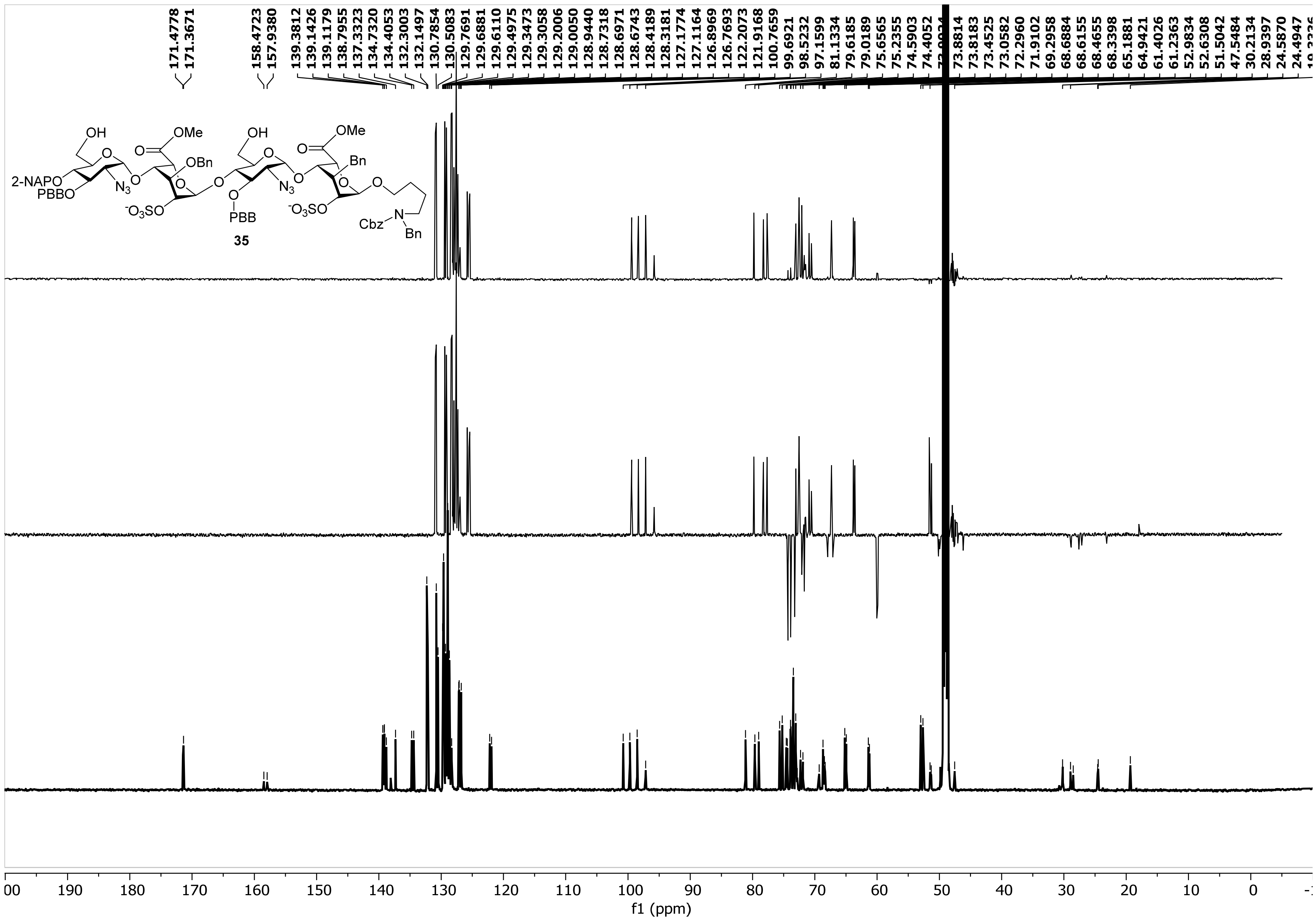
171.4778
171.3671
158.4723
157.9380
139.3812
139.1426
139.1179
138.7955
137.3323
134.7320
134.4053
132.3003
132.1497
130.7854
130.5083
129.7691
129.6881
129.6110
129.4975
129.3473
129.3058
129.2006
129.0050
128.9440
128.7318
128.6971
128.6743
128.4189
128.3181
127.1774
127.1164
126.8969
126.7693
122.2073
121.9168
100.7659
99.6921
98.5232
97.1599
81.1334
79.6185
79.0189
75.6565
75.2355
74.5903
74.4052
73.8814
73.8183
73.4525
73.0582
72.2960
71.9102
69.2958
68.6884
68.6155
68.4655
68.3398
65.1881
64.9421
61.4026
61.2363
52.9834
52.6308
51.5042
47.5484
30.2134
28.9397
24.5870
24.4947
OH
OMe
OBn
2-NAPO
PBBO
N3
-O3SO
O
PBB
Bn
Cbz
N
35
f1 (ppm)

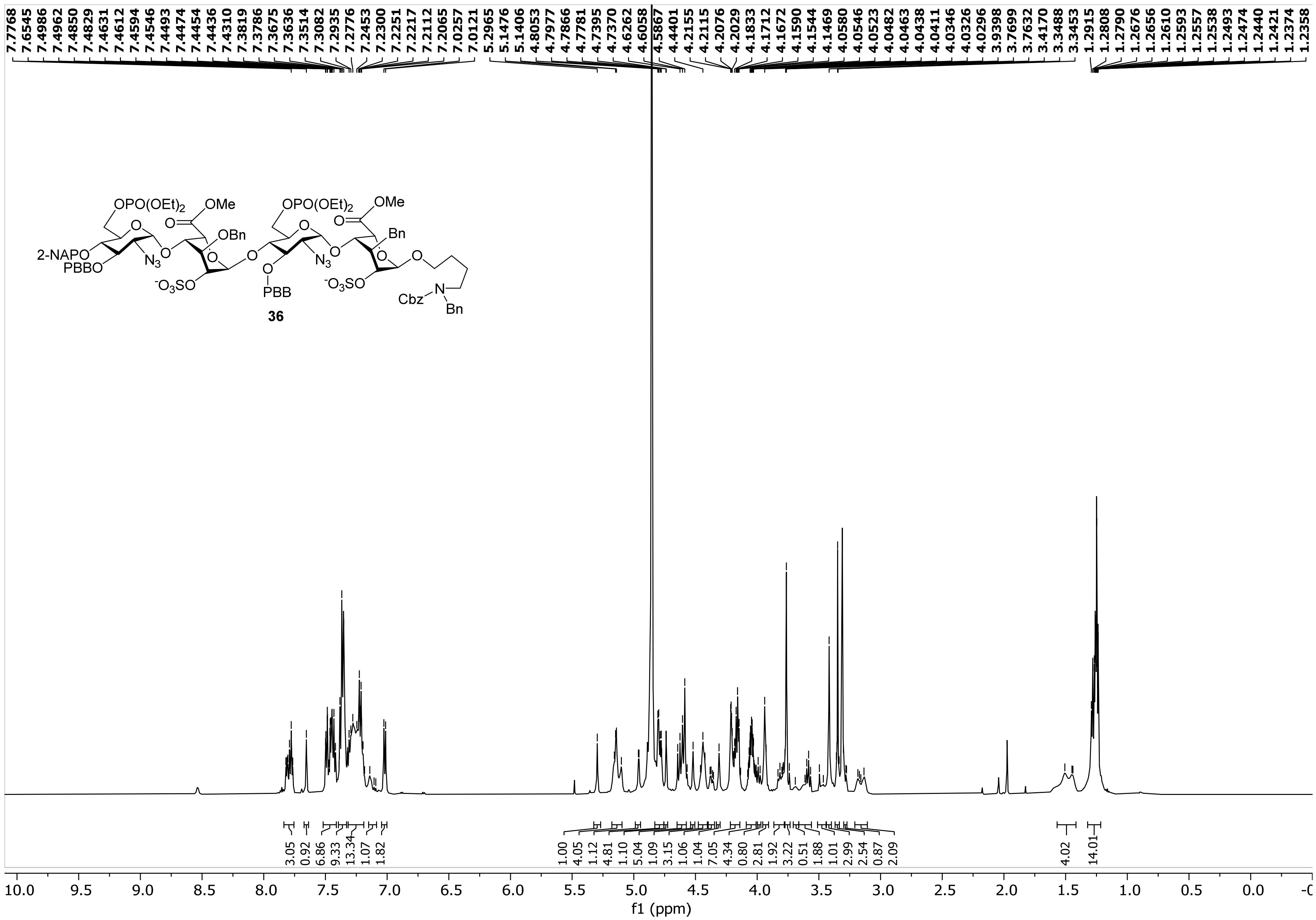
7.7768
7.6545
7.4986
7.4962
7.4850
7.4829
7.4631
7.4612
7.4594
7.4546
7.4493
7.4474
7.4454
7.4436
7.4310
7.3819
7.3786
7.3675
7.3636
7.3514
7.3082
7.2935
7.2776
7.2453
7.2300
7.2251
7.2217
7.2112
7.2065
7.0257
7.0121
5.2965
5.1476
5.1406
4.8053
4.7977
4.7866
4.7781
4.7395
4.7370
4.6262
4.6058
4.5867
4.4401
4.2155
4.2115
4.2076
4.2029
4.1833
4.1712
4.1672
4.1590
4.1544
4.1469
4.0580
4.0546
4.0523
4.0482
4.0463
4.0438
4.0411
4.0346
4.0326
4.0296
3.9398
3.7699
3.7632
3.4170
3.3488
3.3453
1.2915
1.2808
1.2790
1.2676
1.2656
1.2610
1.2593
1.2557
1.2538
1.2493
1.2474
1.2440
1.2421
1.2374
1.2358
OPO(OEt)2
OMe
OBn
Bn
2-NAPO
PBBO
N3
-O3SO
PBB
Cbz
N
36
3.05
0.92
6.86
9.33
13.34
1.07
1.82
1.00
4.05
1.12
4.81
1.10
5.04
1.09
3.15
1.06
1.04
7.05
4.34
0.80
2.81
1.92
3.22
0.51
1.88
1.01
2.99
2.54
0.87
2.09
4.02
14.01
10.0
9.5
9.0
8.5
8.0
7.5
7.0
6.5
6.0
5.5
5.0
4.5
4.0
3.5
3.0
2.5
2.0
1.5
1.0
0.5
0.0
f1 (ppm)

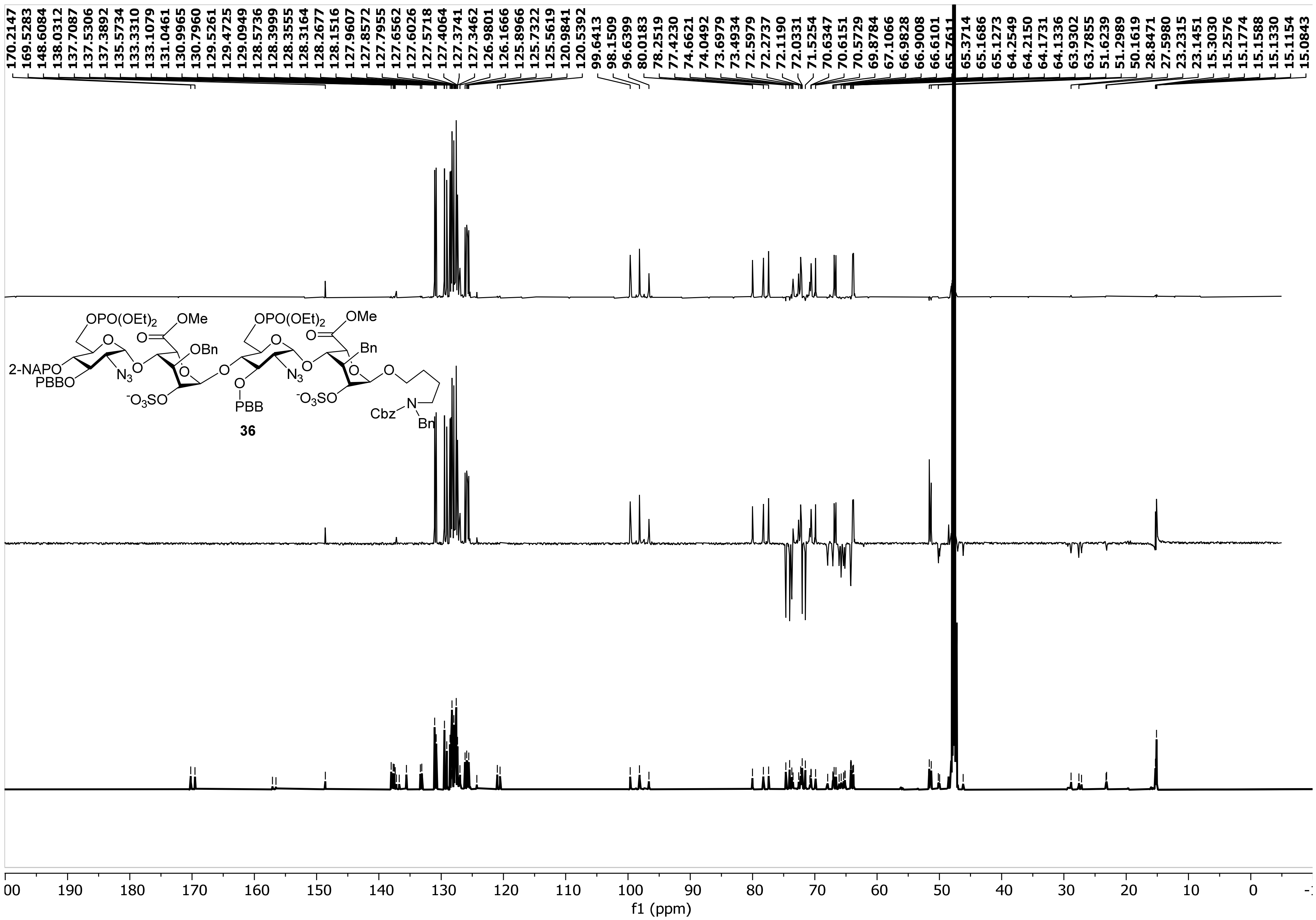
170.2147
169.5283
148.6084
138.0312
137.7087
137.5306
137.3892
135.5734
133.3310
133.1079
131.0461
130.9965
130.7960
129.5261
129.4725
129.0949
128.5736
128.3999
128.3555
128.3164
128.2677
128.1516
127.9607
127.8572
127.7955
127.6562
127.6026
127.5718
127.4064
127.3741
127.3462
126.9801
126.1666
125.8966
125.7322
125.5619
120.9841
120.5392
99.6413
98.1509
96.6399
80.0183
78.2519
77.4230
74.6621
74.0492
73.6979
73.4934
72.5979
72.2737
72.1190
72.0331
71.5254
70.6347
70.6151
70.5729
69.8784
67.1066
66.9828
66.9008
66.6101
65.7611
65.3714
65.1686
65.1273
64.2549
64.2150
64.1731
64.1336
63.9302
63.7855
51.6239
51.2989
50.1619
28.8471
27.5980
23.2315
23.1451
15.3030
15.2576
15.1774
15.1588
15.1330
15.1154
15.0843
OPO(OEt)2
OMe
2-NAPO
PBBO
N3
OBn
-O3SO
O
PBB
Bn
Cbz
N
36
00
190
180
170
160
150
140
130
120
110
100
90
80
70
60
50
40
30
20
10
0
f1 (ppm)

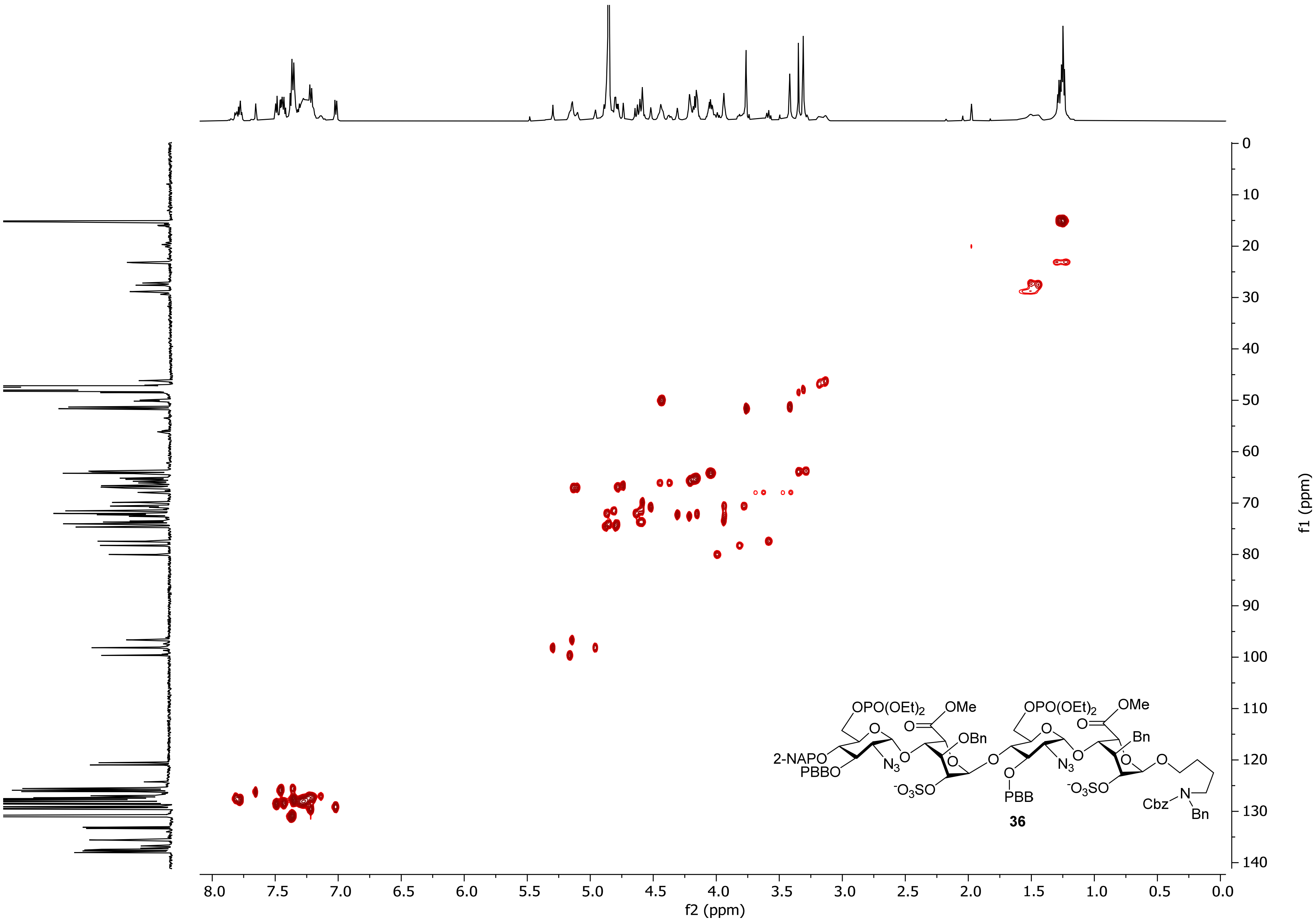

OPO(OEt)2
OMe
OBn
2-NAPO
PBBO
N3
-O3SO
O
PBB
Bn
Cbz
N
36
8.0
7.5
7.0
6.5
6.0
5.5
5.0
4.5
4.0
3.5
3.0
2.5
2.0
1.5
1.0
0.5
0.0
f2 (ppm)
0
10
20
30
40
50
60
70
80
90
100
110
120
130
140
f1 (ppm)

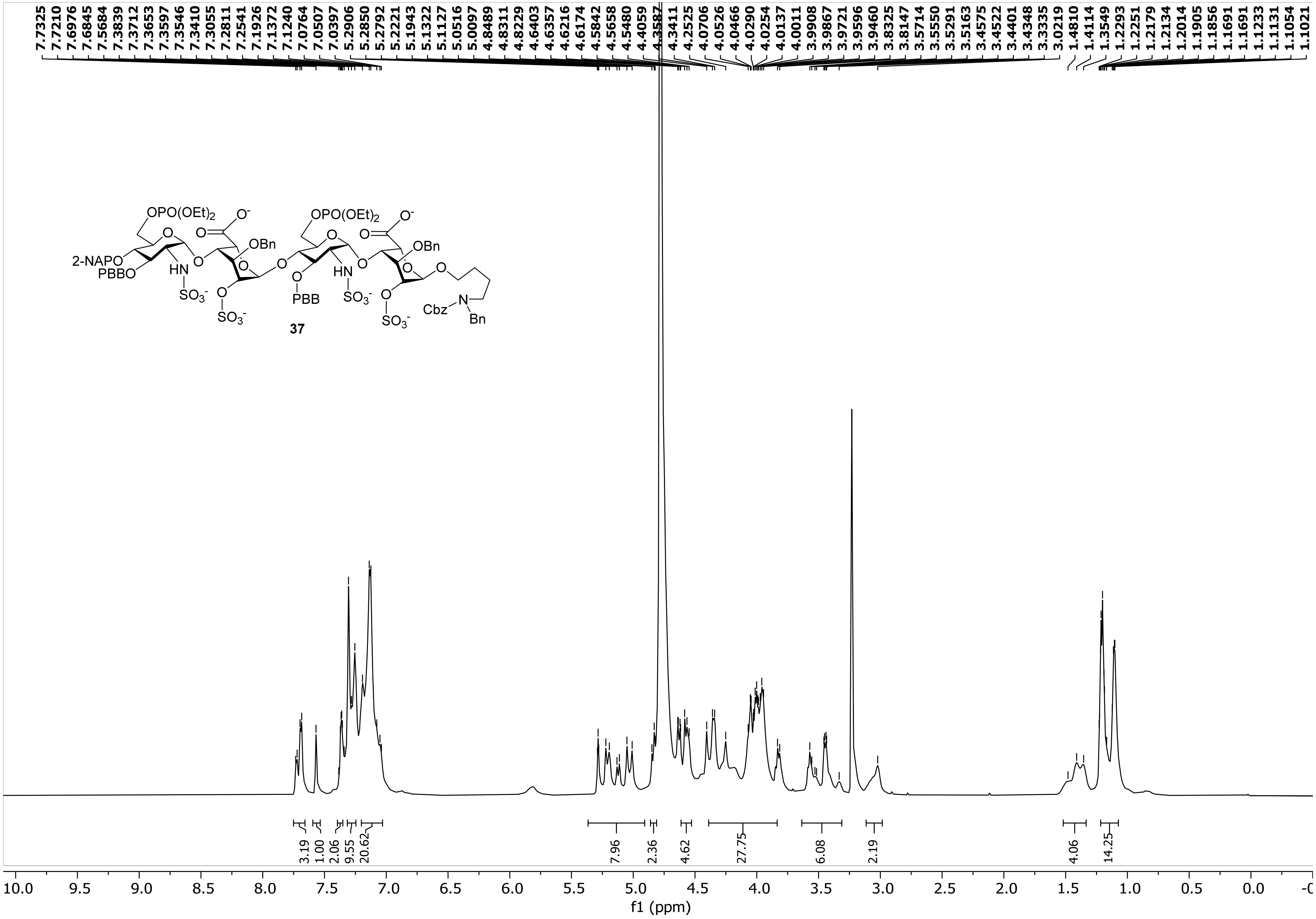
7.7325
7.7210
7.6976
7.6845
7.5684
7.3839
7.3712
7.3653
7.3597
7.3546
7.3410
7.3055
7.2811
7.2541
7.1926
7.1372
7.1240
7.0764
7.0507
7.0397
5.2906
5.2850
5.2792
5.2221
5.1943
5.1322
5.1127
5.0516
5.0097
4.8489
4.8311
4.8229
4.6403
4.6357
4.6216
4.6174
4.5842
4.5658
4.5480
4.4059
4.3587
4.3411
4.2525
4.0706
4.0526
4.0466
4.0290
4.0254
4.0137
4.0011
3.9908
3.9867
3.9721
3.9596
3.9460
3.8325
3.8147
3.5714
3.5550
3.5291
3.5163
3.4575
3.4522
3.4401
3.4348
3.3335
3.0219
1.4810
1.4114
1.3549
1.2293
1.2251
1.2179
1.2134
1.2014
1.1905
1.1856
1.1691
1.1691
1.1233
1.1131
1.1054
1.1021
OPO(OEt)2
O-
2-NAPO
PBBO
HN
SO3-
OBn
SO3-
PBB
37
Cbz
N
Bn
3.19
1.00
2.06
9.55
20.62
7.96
2.36
4.62
27.75
6.08
2.19
4.06
14.25
f1 (ppm)

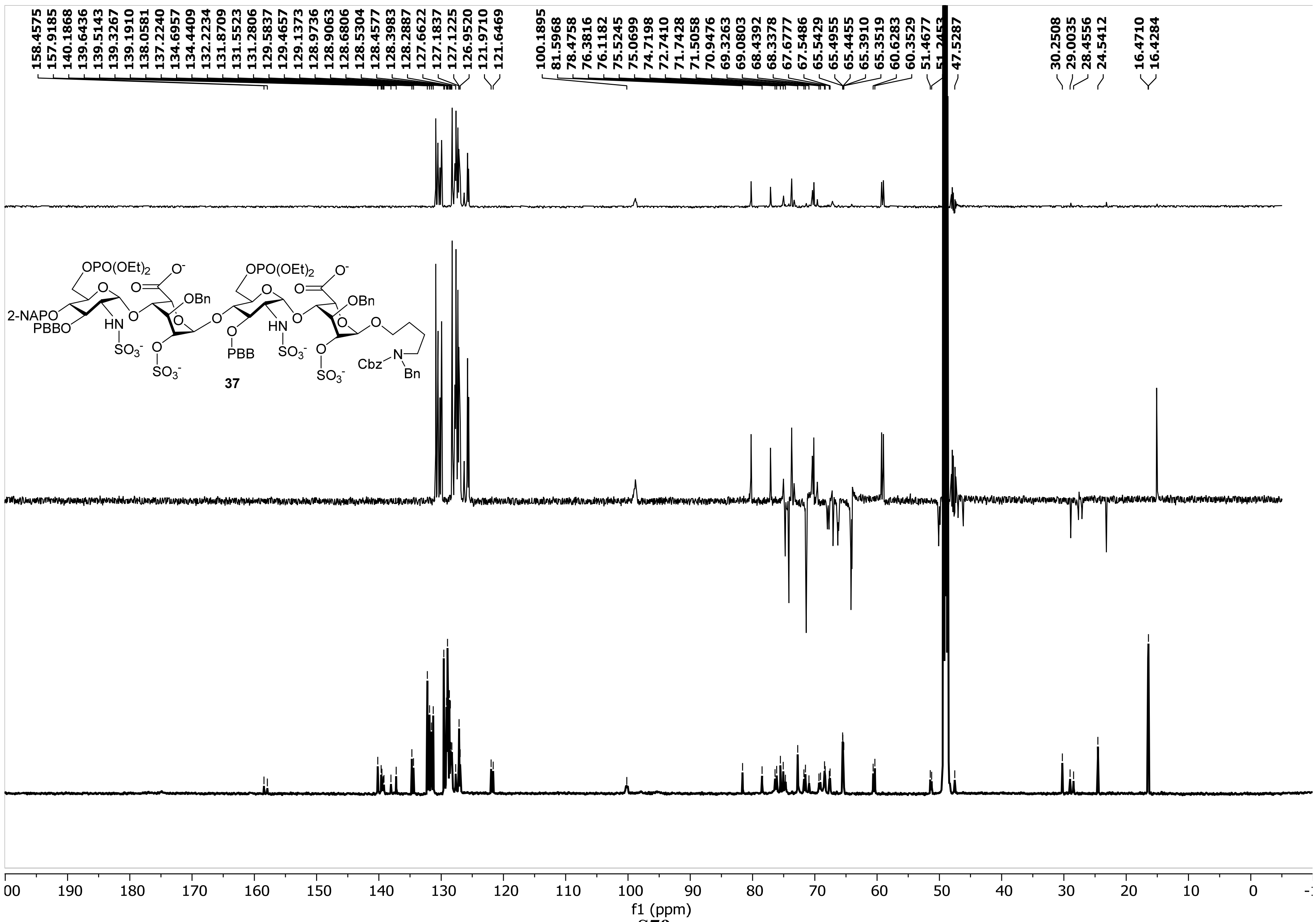
158.4575
157.9185
140.1868
139.6436
139.5143
139.3267
139.1910
138.0581
137.2240
134.6957
134.4409
132.2234
131.8709
131.5523
131.2806
129.5837
129.4657
129.1373
128.9736
128.9063
128.6806
128.5304
128.4577
128.3983
128.2887
127.6622
127.1837
127.1225
126.9520
121.9710
121.6469
100.1895
81.5968
78.4758
76.3816
76.1182
75.5245
75.0699
74.7198
72.7410
71.7428
71.5058
70.9476
69.3263
69.0803
68.4392
68.3378
67.6777
67.5486
65.5429
65.4955
65.4455
65.3910
65.3519
60.6283
60.3529
51.4677
51.2453
47.5287
30.2508
29.0035
28.4556
24.5412
16.4710
16.4284
OPO(OEt)2
O-
O
OBn
2-NAPO
PBBO
HN
SO3-
PBB
Cbz
N
Bn
37
f1 (ppm)

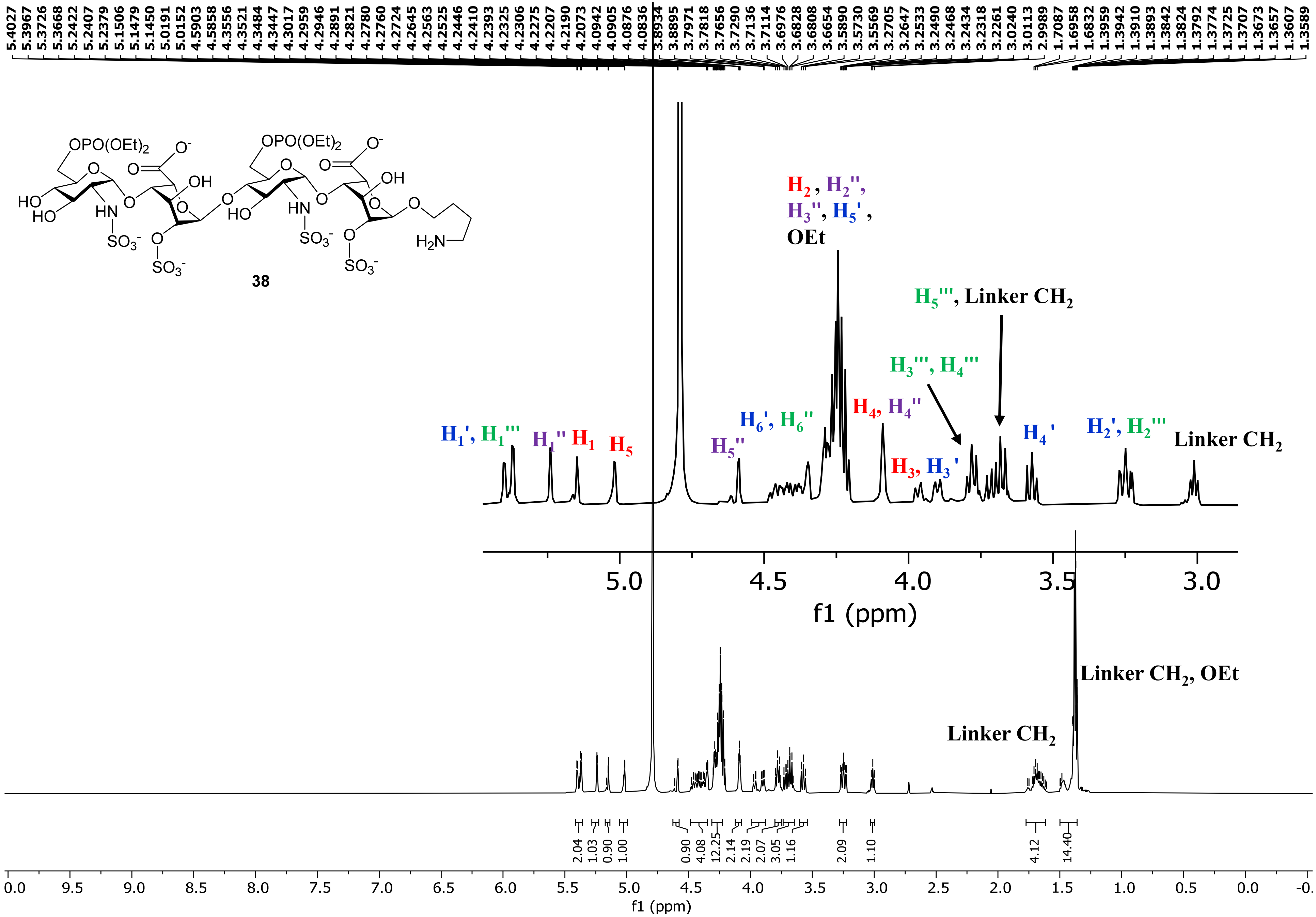
OPO(OEt)2
O-
OH
HO
HO
HN
SO3-
O
HN
SO3-
H2N

38

5.4027 5.3967 5.3726 5.3668 5.2422 5.2407 5.2379 5.1506 5.1479 5.1450 5.0191 5.0152 4.5903 4.5858 4.3556 4.3521 4.3484 4.3447 4.3017 4.2959 4.2946 4.2891 4.2821 4.2780 4.2760 4.2724 4.2645 4.2563 4.2525 4.2446 4.2410 4.2393 4.2325 4.2306 4.2275 4.2207 4.2190 4.2073 4.0942 4.0905 4.0876 4.0836 3.8934 3.8895 3.7971 3.7818 3.7656 3.7290 3.7136 3.7114 3.6976 3.6828 3.6808 3.6654 3.5890 3.5730 3.5569 3.2705 3.2647 3.2533 3.2490 3.2468 3.2434 3.2318 3.2261 3.0240 3.0113 2.9989 1.7087 1.6958 1.6832 1.3959 1.3942 1.3910 1.3893 1.3842 1.3824 1.3792 1.3774 1.3725 1.3707 1.3673 1.3657 1.3607 1.3589
H2, H2'', H3'', H5', OEt
H5''', Linker CH2
H3''', H4'''
H4, H4''
H6', H6''
H1', H1'''
H1'' H1
H5
H5''
H3, H3'
H4'
H2', H2'''
Linker CH2
5.0 4.5 4.0 3.5 3.0
f1 (ppm)
Linker CH2, OEt
Linker CH2
2.04 1.03 0.90 1.00 0.90 4.08 12.25 2.14 2.19 2.07 3.05 1.16 2.09 1.10 4.12 14.40
10.0 9.5 9.0 8.5 8.0 7.5 7.0 6.5 6.0 5.5 5.0 4.5 4.0 3.5 3.0 2.5 2.0 1.5 1.0 0.5 0.0 -0.
f1 (ppm)

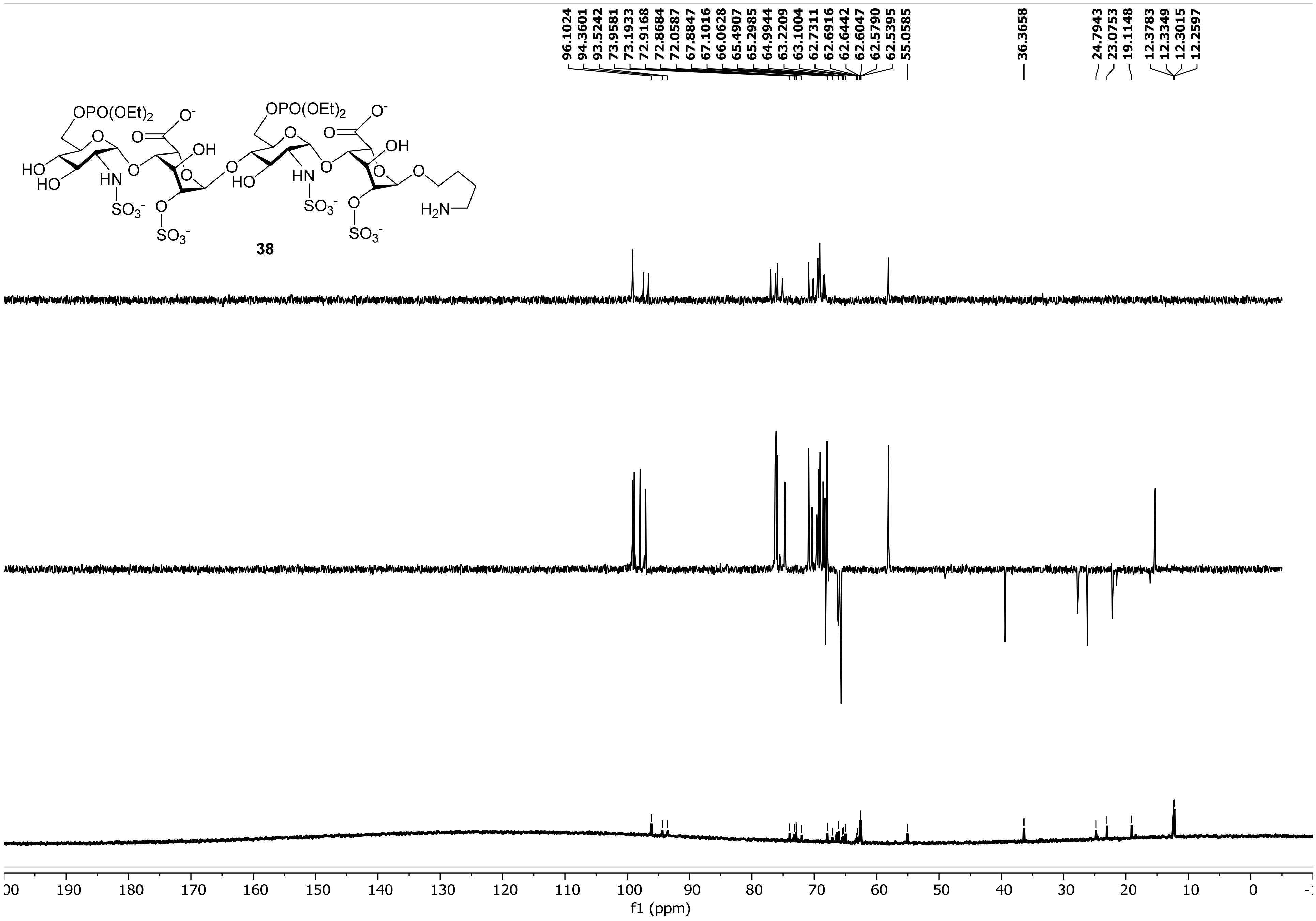
96.1024
94.3601
93.5242
73.9581
73.1933
72.9168
72.8684
72.0587
67.8847
67.1016
66.0628
65.4907
65.2985
64.9944
63.2209
63.1004
62.7311
62.6916
62.6442
62.6047
62.5790
62.5395
55.0585
36.3658
24.7943
23.0753
19.1148
12.3783
12.3349
12.3015
12.2597
OPO(OEt)2
O-
HO
HO
HN
SO3-
OH
SO3-
HO
H2N
38
190
180
170
160
150
140
130
120
110
100
90
80
70
60
50
40
30
20
10
0
f1 (ppm)

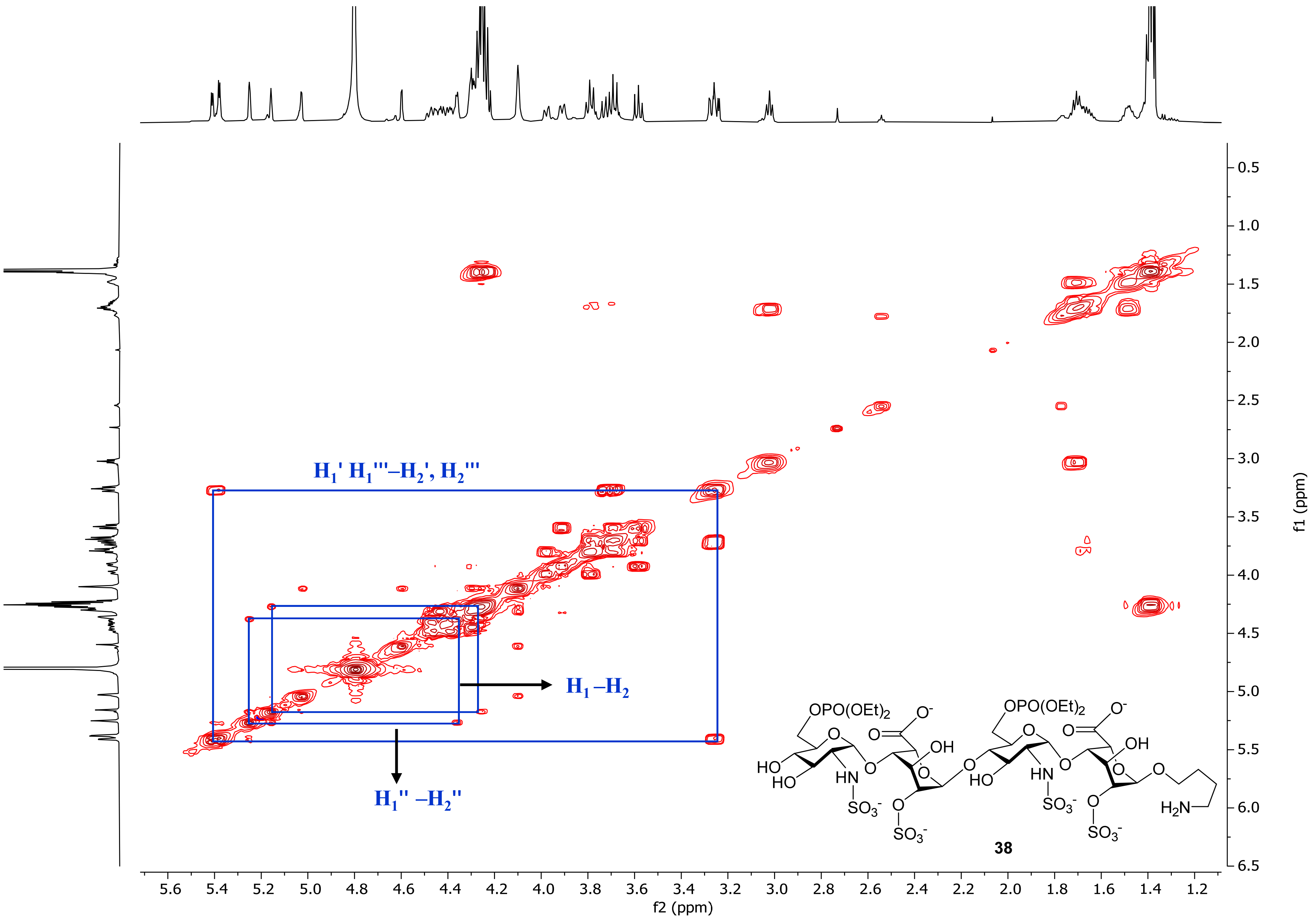

H1' H1'''–H2', H2'''
H1 –H2
H1'' –H2''
OPO(OEt)2
HO
HO
HN
SO3-
O
OH
H2N
38
f2 (ppm)
f1 (ppm)

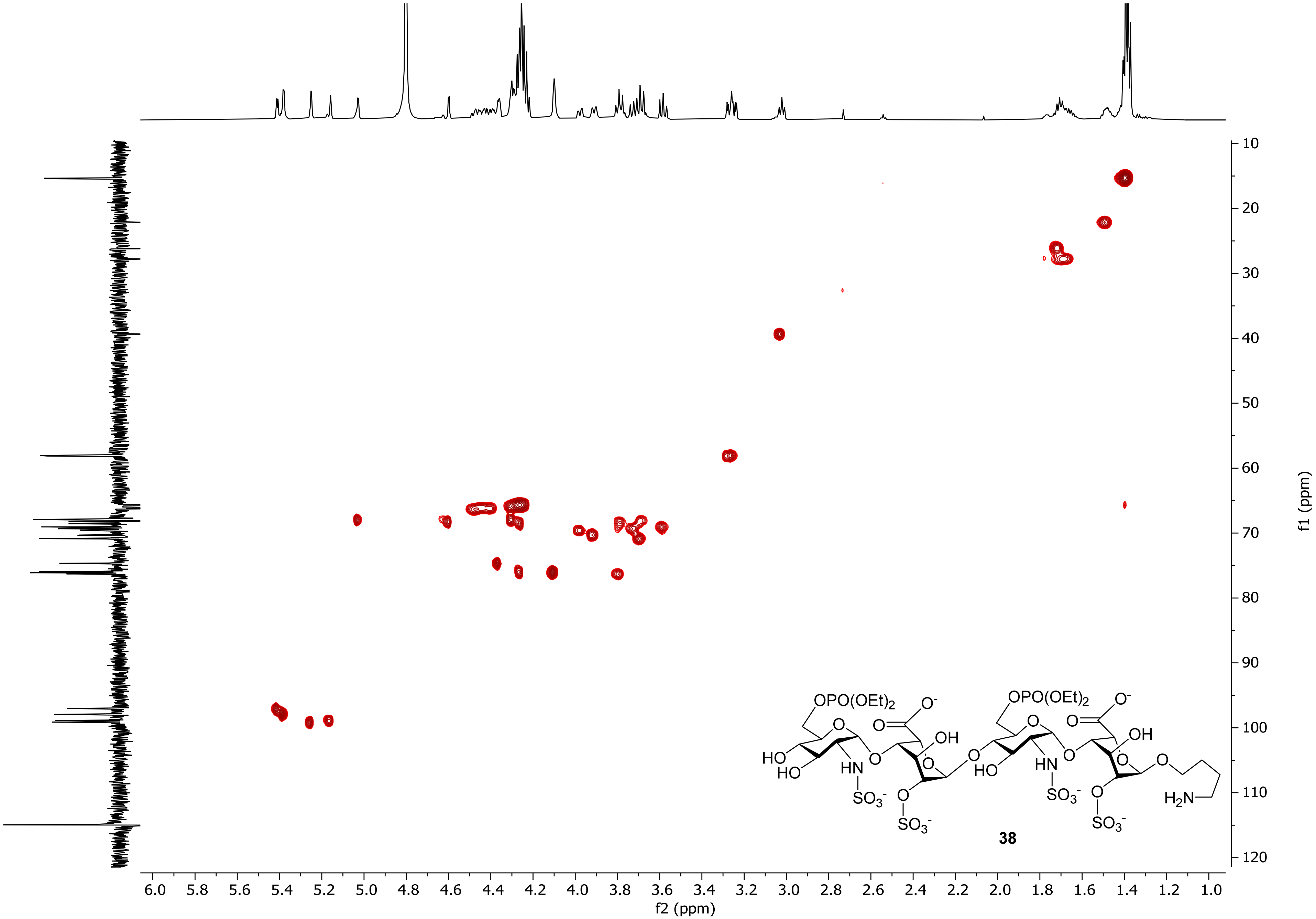
OPO(OEt)2
O-
O
OH
HO
HO
HN
SO3-
HO
H2N
38
f2 (ppm)
f1 (ppm)
6.0 5.8 5.6 5.4 5.2 5.0 4.8 4.6 4.4 4.2 4.0 3.8 3.6 3.4 3.2 3.0 2.8 2.6 2.4 2.2 2.0 1.8 1.6 1.4 1.2 1.0
10 20 30 40 50 60 70 80 90 100 110 120

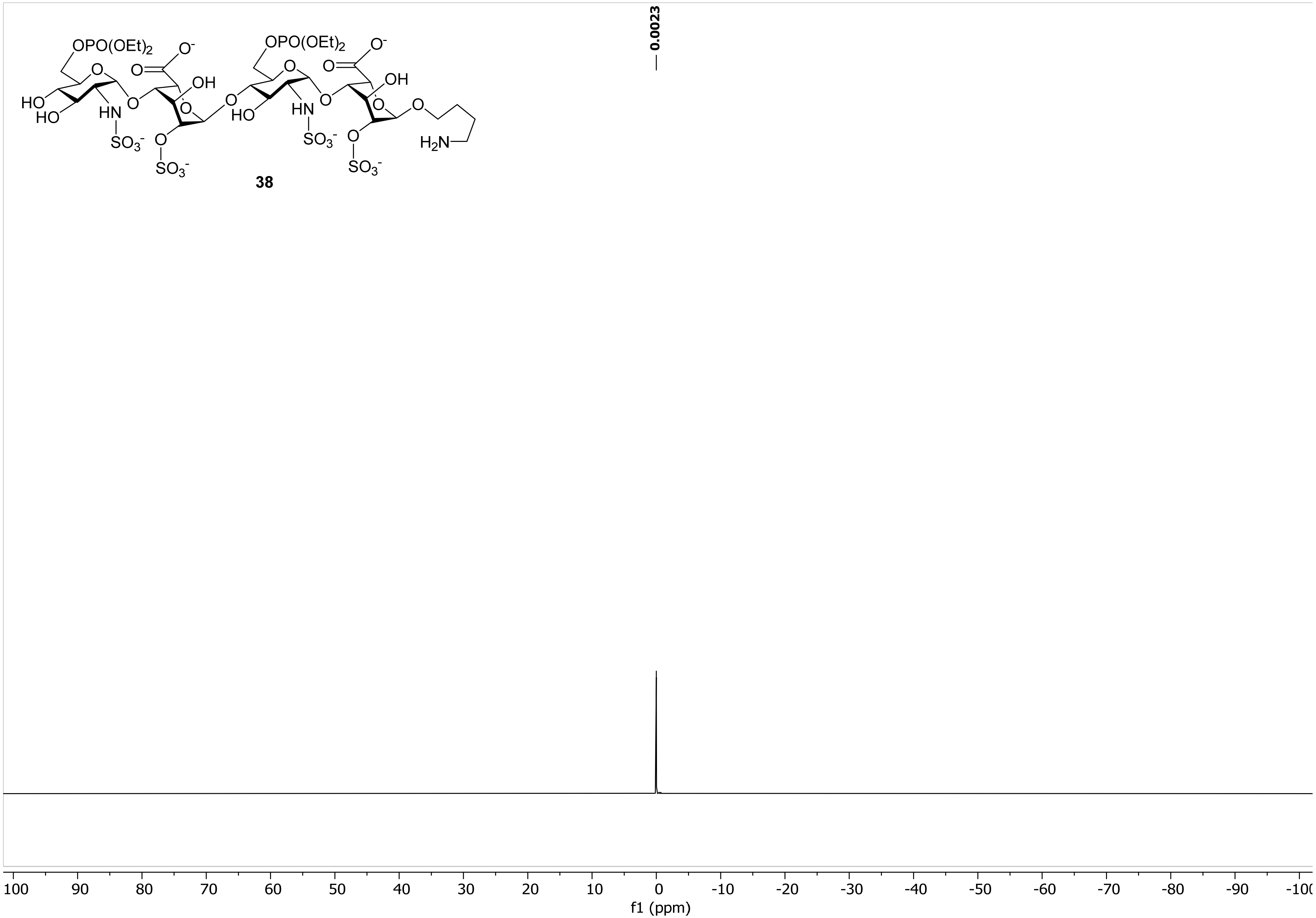
OPO(OEt)2
O-
OH
HO
HO
HN
SO3-
O
HN
SO3-
H2N
38
0.0023
100 90 80 70 60 50 40 30 20 10 0 -10 -20 -30 -40 -50 -60 -70 -80 -90
f1 (ppm)
